# Plant and insect herbivore community variation across the Paleocene–Eocene boundary in the Hanna Basin, southeastern Wyoming

**DOI:** 10.7717/peerj.7798

**Published:** 2019-10-15

**Authors:** Lauren E. Azevedo Schmidt, Regan E. Dunn, Jason Mercer, Marieke Dechesne, Ellen D. Currano

**Affiliations:** 1 Botany, University of Wyoming, Laramie, WY, USA; 2 Natural History Museums of Los Angeles County, La Brea Tar Pits, Los Angeles, CA, USA; 3 U.S. Geological Survey, Geosciences and Environmental Change Science Center, Denver, CO, USA; 4 Geology and Geophysics, University of Wyoming, Laramie, WY, USA

**Keywords:** Paleobotany, Paleoecology, Plant and insect interactions, Climate change, PETM, Paleontology, Insect damage, Fossil record, Hanna Basin

## Abstract

Ecosystem function and stability are highly affected by internal and external stressors. Utilizing paleobotanical data gives insight into the evolutionary processes an ecosystem undergoes across long periods of time, allowing for a more complete understanding of how plant and insect herbivore communities are affected by ecosystem imbalance. To study how plant and insect herbivore communities change during times of disturbance, we quantified community turnover across the Paleocene­–Eocene boundary in the Hanna Basin, southeastern Wyoming. This particular location is unlike other nearby Laramide basins because it has an abundance of late Paleocene and Eocene coal and carbonaceous shales and paucity of well-developed paleosols, suggesting perpetually high water availability. We sampled approximately 800 semi-intact dicot leaves from five stratigraphic levels, one of which occurs late in the Paleocene–Eocene thermal maximum (PETM). Field collections were supplemented with specimens at the Denver Museum of Nature & Science. Fossil leaves were classified into morphospecies and herbivore damage was documented for each leaf. We tested for changes in plant and insect herbivore damage diversity using rarefaction and community composition using non-metric multidimensional scaling ordinations. We also documented changes in depositional environment at each stratigraphic level to better contextualize the environment of the basin. Plant diversity was highest during the mid-late Paleocene and decreased into the Eocene, whereas damage diversity was highest at the sites with low plant diversity. Plant communities significantly changed during the late PETM and do not return to pre-PETM composition. Insect herbivore communities also changed during the PETM, but, unlike plant communities, rebound to their pre-PETM structure. These results suggest that insect herbivore communities responded more strongly to plant community composition than to the diversity of species present.

## Introduction

Many modern ecological studies focus on how ecosystems will adapt over the next 100 years as anthropogenic climate change continues to alter terrestrial ecosystems, oftentimes faster than plants and insects can adapt (Parmesan, 2006; Anderson, Panetta & Mitchell-Olds, 2012; Midgley & Bond, 2015). What is to come after the hundred-year mark? Paleobotanical research allows ecological questions to extend the century-long time scale by several orders of magnitude, providing insight into the evolutionary processes of ecosystems as they respond to environmental changes. Plant and insect herbivore food webs are ideal for studying ecosystem function as they are highly influenced by internal and external factors such as changes in the diversity and composition of primary producers and abiotic environmental conditions (Coviella & Trumble, 1999). Food web dynamics of plants and insects are preserved within the rock record as biological markers of ecosystem function, allowing for a deep-time understanding of how these communities responded to gradual and abrupt environmental changes as well as potentially giving clues to what is the driving force behind the change (Labandeira & Currano, 2013). By better understanding how ancient ecosystems record both stability and instability within plant and insect food webs, it may be possible to better interpret changes within modern ecosystems and improve predictions on how future anthropogenic climate change will alter terrestrial trophic dynamics.

Previous paleoecological studies have detailed how local communities were affected by abrupt, global disruptions such as the Late Cretaceous bolide impact (66 million years ago, or Ma) and the Paleocene–Eocene thermal maximum (PETM, 56 Ma). Modern ecological studies have shown that plant and insect diversity are often correlated; however, this is not the case after the Cretaceous-Paleogene extinction event (K-Pg). Following the Cretaceous bolide impact, ecosystem function was highly variable among regions, as terrestrial plant and insect communities experienced rebound (Vajda, Raine & Hollis, 2001; Ellis, Johnson & Dunn, 2003; Wilf et al., 2006; Iglesias et al., 2007; Wappler et al., 2009; Donovan et al., 2016). This instability continued for the duration of the Paleocene in western North America, as evidenced by inconsistencies in plant vs. insect herbivore diversity (Wilf et al., 2006). As ecosystems in the Western Interior, U.S.A. rebounded, they also experienced changing climate, from the relatively cooler Paleocene to the “hothouse” Eocene (Zachos, Dickens & Zeebe, 2008). This gradual warming was punctuated by the PETM, a geologically abrupt perturbation to the global carbon cycle and ultimately the energy budget of the planet (McInerney & Wing, 2011). This event, indicated in the rock record by a negative carbon isotope excursion resulting from a massive release of greenhouse gases, caused global temperatures to increase ~5–9 °C and caused varied changes in precipitation regimes (McInerney & Wing, 2011). The PETM transformed terrestrial ecosystems in many ways, including the alteration and intensification of hydrologic cycles (Schmitz & Pujalte, 2007; Handley et al., 2008; Kraus et al., 2013), an increase in sedimentary flow rates and change in depositional systems (Foreman, Heller & Clementz, 2012), turnover in abundant plant species (Smith, Wing & Freeman, 2007; Wing & Currano, 2013; Garel et al., 2014), and increased insect herbivory (Currano et al., 2008, 2016).

**Figure 1:**
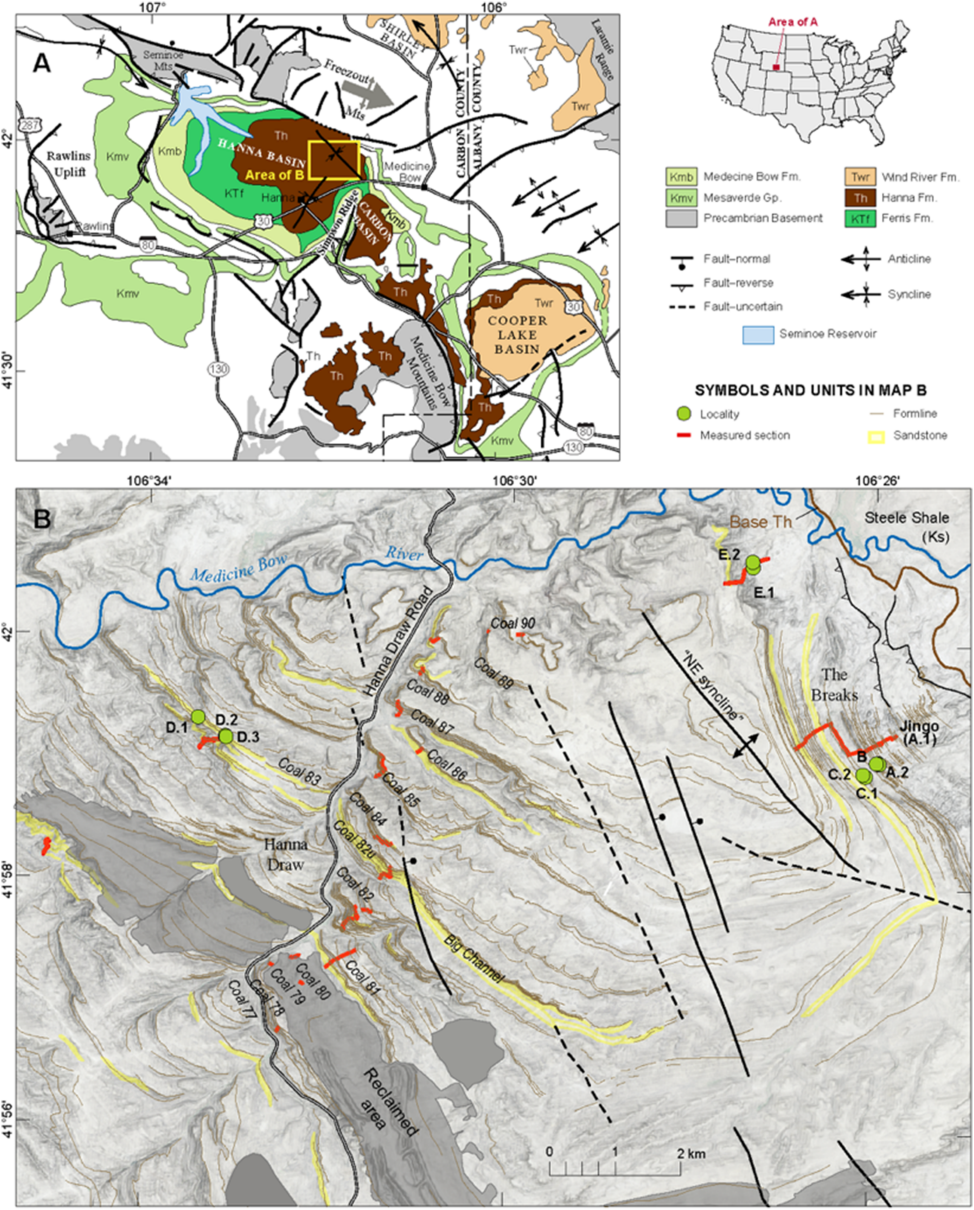
Basin Map. (A) Geologic map of Hanna Basin, WY. Inset of US map shows location of the Hanna Basin within Wyoming. (B) Base map of Hanna Basin showing the location of each quarry site, modified from M. Dechesne, 2019, personal communication.

These studies encompass a limited geographic area; however due to the importance of the time interval, new localities capturing the terrestrial ecosystem are needed. The Hanna Basin, southeastern Wyoming (Fig. 1) is ideal for research because of its thick and uninterrupted sequence of Paleocene-Eocene sediments (Smith & Bowen, 1918), newly described PETM sections (M. Dechesne, 2019, personal communication), and rock types that suggest a different microclimate and paleoenvironment than existed in other well-studied Rocky Mountain basins. In contrast to the well-studied Eocene Willwood Formation in the Bighorn Basin, which contains highly oxidized red beds (Kraus, 2001), the Hanna Formation contains abundant coal beds, carbonaceous shales, and drab sandstones and siltstones that were deposited from the Paleocene through the early Eocene (Dobbin, Bowen & Hoots, 1929; Lillegraven & Snoke, 1996; M. Dechesne, 2019, personal communication). These strata represent primarily fluvial, lacustrine and back swamp deposition with organic-rich poorly drained soils (i.e., histosols) that indicate high water availability throughout the interval. If Paleogene plant and insect communities had the same basic needs as modern ones, Hanna Basin plant and insect communities would not have been limited by water. Also, with elevated atmospheric CO_2_ in the Paleogene (Freeman & Hayes, 1992), carbon would not have been limiting for plant growth either.

The goal of this research is to track changes in plant and insect communities across the Paleocene–Eocene boundary within the Hanna Basin and to better understand how changes in the global energy budget are reflected at the regional level. Our dataset spans the late Paleocene–early Eocene and integrates information from new field collections, museum specimens and a literature review. Paleoclimate reconstructions were made for our study site to understand how and if regional climate reflected changes in the global energy budget and to contextualize changes in local plant and insect herbivore communities. Additionally, by investigating plant and insect dynamics as global climate change occurred and depositional environment varied within the basin, we can better apply our findings to future predictions of terrestrial ecosystem function through a turbulent time.

## Geologic setting

The western region of North America underwent substantial structural alterations during the Late Cretaceous through the Eocene, starting with the closing of the Western Interior seaway. Additionally, the Laramide orogeny, a low angle flat-slab subduction, transformed the landscape gradually and altered the greater foreland basin into the Eocene (Dickinson et al., 1988). This alteration resulted in smaller basins isolated by mountain ranges and uplifted arches (Ryan, 1977; Dickinson et al., 1988) that extend from Canada (~54°N) to New Mexico (~34°N) and captured thick, continuous sections of early Paleogene sediments. The Hanna Basin, an asymmetrical fault-bounded basin located in southeastern Wyoming, is one such basin. Although relatively small (~2,600 km^2^), it is a deep basin with thick, continuous sections of Late Cretaceous through Eocene sedimentary rock (Blackstone, 1993).

The formation of interest in this study is the Hanna Formation which is approximately 2,100 m in thickness and composed of alternating coal, shale, and sandstone (Smith & Bowen, 1918; Dobbin, Bowen & Hoots, 1929). This formation has been interpreted as being fluvial, paludal and lacustrine in deposition, with gradual shifts occurring between depositional environments (M. Dechesne, 2019, personal communication). Fluvial and swamp deposits are more prevalent in the central and western parts of the basin, whereas paludal and lacustrine sediments dominate to the east. The basin is dominated by paludal to lacustrine facies during the mid to late Paleocene (post ~59 Ma) and transitions to a fluvial dominated environment during the PETM. The PETM interval was determined by Dechesne et al. (2017) using plant biostratigraphy (pollen and macrofossils) and δ^13^C bulk organic carbon analyses. It includes coarse grained, laterally extensive channel deposits referred to by Dechesne and colleagues as “Big Channel.” The depositional environment changed following the PETM as basin subsidence and infill continued, resulting in a dominance of paludal to gradually more lacustrine strata (Dechesne et al., 2017).

Early and middle Paleocene floras from the Hanna Fm. and underlying Ferris Fm. were described by Regan Dunn in an unpublished master’s thesis (Dunn, 2003); Dunn’s work includes analyses of plant species composition, richness and abundance as well as paleobotanical reconstructions of mean annual temperature (MAT) and precipitation. Her study found that changes in Paleocene floral richness were not linked to changes in reconstructed mean annual precipitation (MAP) or MAT, but rather richness increased gradually through time following the K–Pg extinction (Dunn, 2003). The two youngest sites collected by Dunn, Jingo (DMNH l.2725) and Wing Ding (DMNH l.2630), are in the upper Hanna Fm. and are included in this study. Here, we extend Dunn’s work by documenting latest Paleocene and early Eocene paleofloral composition within the Hanna Fm., reconstructing MAT and MAP for these floras, and analyzing insect herbivore damage on the fossil floras.

## Materials and Methods

### Field sites

**Figure 2:**
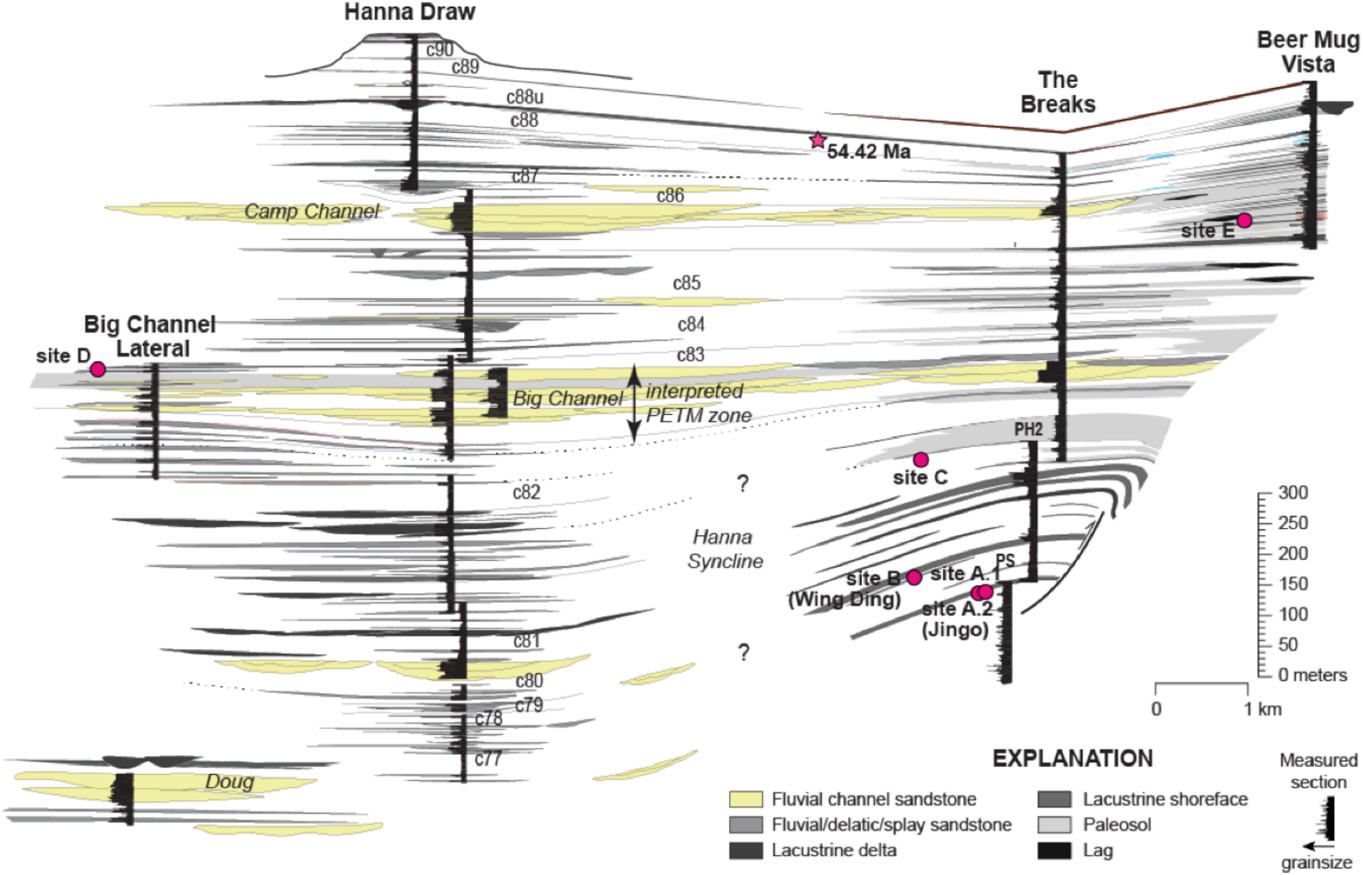
Stratigraphic framework for the Hanna Basin, late Paleocene–Early Eocene. Stratigraphic placement for localities described in this paper within the Hanna Formation, along with correlations within the Hanna Basin. C numbers indicate coalbeds labeled after Dobbin, Bowen & Hoots (1929), star indicates zircon date; a U/Pb concordia age of 54.42 ± 0.27 Ma was reported by M. Dechesne, 2019, personal communication but this date is likely a maximum estimate because the majority of zircons measured were detrital and the youngest dates were obtained from euhedral crystals.

Field collections took place on private land with the authorization of Burt and Kay Lynn Palm of the Palms Ranch, and Tad Anderson of Q Creek Ranch. We analyzed 807 semi-intact fossil dicot leaves from eight sites at five distinct stratigraphic levels (referred to here as Levels A–E, from lowest to highest) within the Hanna Basin (Table 1). These strata span the late Paleocene, PETM and early Eocene. Sites occur in two areas of the Hanna Basin, The Breaks and Hanna Draw (Fig. 1), which are located approximately eight km apart. Correlations between study areas were mapped in the field and via aerial imagery (Google Earth). The stratigraphic levels studied in The Breaks were structurally deformed dipping ~43° to the N-NW, whereas the strata within the Hanna Draw section dips more shallowly (~14° to the N-NE). Stratigraphic placement of the floral sites follows the coal bed nomenclature of Dobbin, Bowen & Hoots (1929), in which coal beds and carbonaceous shales were sequentially numbered from oldest to youngest. All sites were placed within a detailed, high-resolution stratigraphic framework, which includes approximately 1,250 m of section at Hanna Draw and approximately 1,100 m within The Breaks (Dechesne et al., 2017; Fig. 2). The age of the top of the section is constrained by a ^238^U/^206^Pb zircon date of 54.42 ± 0.27 Ma obtained using four zircon crystals in a possible tonstein located above our highest stratigraphic level (Dechesne et al., 2017; Fig. 2). However, most zircons analyzed in this sample were Proterozoic or Paleozoic in age, suggesting that the zircons are detrital, and 54.42 Ma represents a maximum estimate for deposition of this layer (M. Dechesne, 2019, personal communication). Ages for the oldest floral localities studied here were estimated using linear sedimentation rates constrained by the last occurrence of Tiffanian-3 mammals in The Breaks (estimated to be ~59.5 Ma (see (Secord et al., 2006) for age control, and (Higgins, 2003) for mammal biostratigraphy)) and the age of the CIE (56 Ma, Westerhold et al., 2009).

**Table 1:** Locality summary table.

	LATE PALEOCENE					PETM			EARLY EOCENE	
	A.1	A.2	B	C.1	C.2	D.1	D.2	D.3	E.1	E.2
Sample size (*n*)	224*	92	452*	229	75	5	40	11	61	265
Species richness (quarry)	20*	13	41*	12	10	3	10	6	12	16
Species richness (strat. level)	30		41*	13		13			17	
LMAT (°C)	18.48 ± 2.6		18.8* ± 2.6	22.3 ± 3.4		22.3 ± 3.9			19.1 ± 3.7	
MAP (cm/year)	145 + 62.6 −43.7		184* + 79.3 −55.4	99 + 42.7 −29.82		104 + 45.0 −31.4			142 + 61.2 −42.8	
Lithology and depositional environment	Fluvial/lacustrine. Thinly bedded siltstone and fine-grained sandstones with plant debris and ripples			Lacustrine. Thinly bedded sand and siltstone with alternating ripple beds		Oxbow pond. Laminated bedding and fine to very fine-grained sandstone.	Fluvial. Massive sandstone body with fine-grained sediment. Big Channel complex.		Fluvial. Large sandstone body with coarse (almost gravel) to fine-grained sand. Yellow in color.	

**Note:**

Equation (1)–(3) were used for climate reconstruction. All data collected, and analyses made by Dunn (2003) are denoted with a superscript of (*). Climate estimates for Level A were made using a pooled dataset of Dunn’s collections and the 2017 collections.

**Figure 3:**
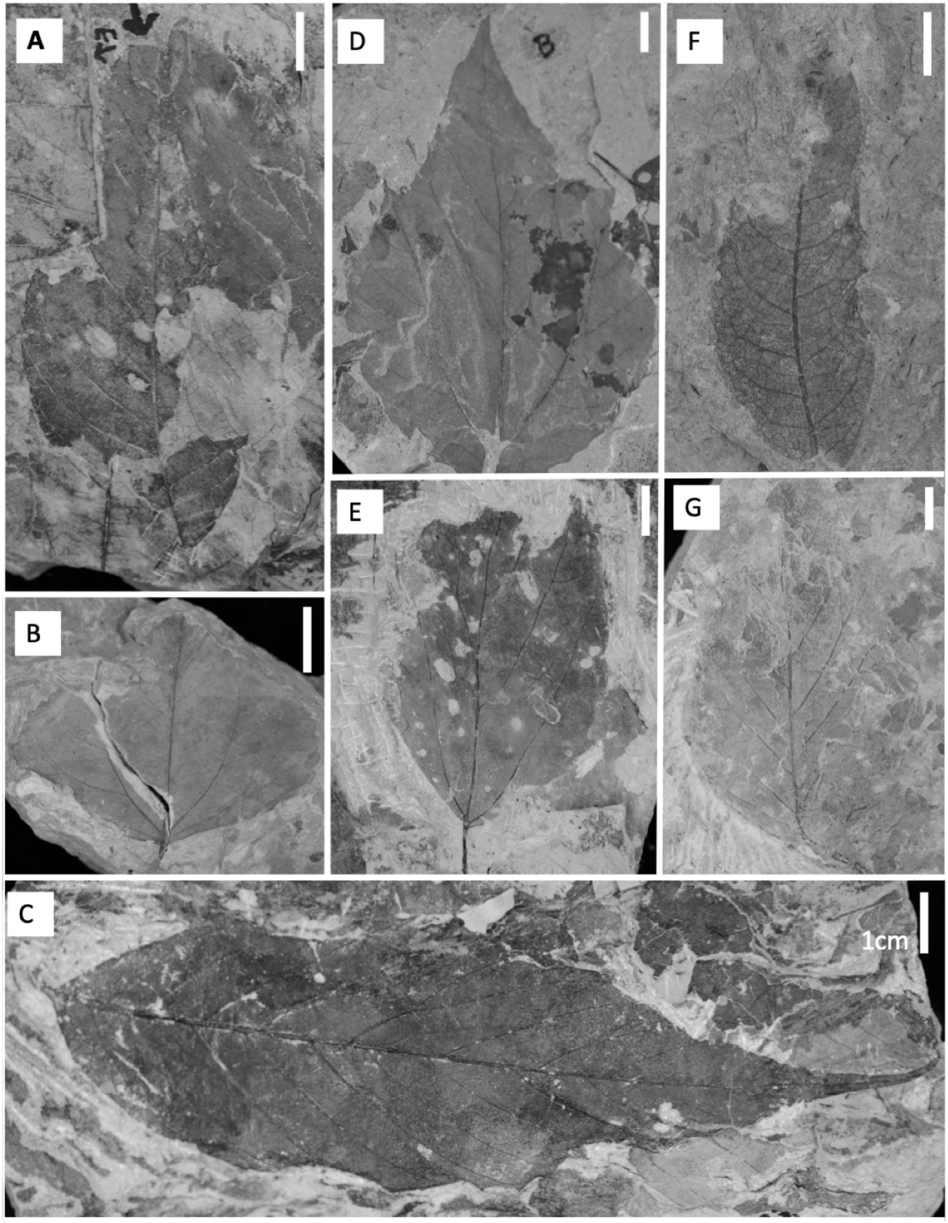
Common dicot leaf types from the late Paleocene and early Eocene in the Hanna Basin. (A) Unnamed dicot morphospecies HB174 (locality D, specimen #PB928), (B) *Trochodendroides genetrix* (locality A, specimen #PB956), (C) unnamed dicot morphospecies HB176 (locality E, specimen #PB978), (D) Palmate leaflet of *Platanites raynoldsii* (locality A, specimen #PB968), (E) unnamed dicot morphospecies HB173 (locality D, specimen PB966), (F) *Averrhoites affinis* (locality C, specimen #PB913), (G) unnamed dicot morphospecies HB180 (locality A, specimen PB1063). Scale bars are one cm.

For all paleobotanical sites in this study, field counts of known plant morphospecies with no herbivory damage were tallied on the outcrop and discarded and voucher specimens were collected. All other fossils collected were labeled, wrapped, and transported back to the University of Wyoming for further analysis (Fig. 3). Vouchered specimens from the new collections are housed at the University of Wyoming Geological Museum.

The lowest stratigraphic level of this study (Level A), located approximately between Coal 77 and Coal 78 in The Breaks, includes Jingo quarry (referred to here as Site A.1), previously sampled by Dunn (2003). Jingo is approximately 2,090 m (Fig. 2) above the local base of the Hanna Fm. in The Breaks as measured by Lillegraven & Snoke (1996). As museum collections (Denver Museum of Nature & Science) from this site were insufficient for herbivory analyses, additional leaf fossils were sampled from a new bench quarry (Site A.2) located 0.25 km to the southeast, but lateral to Dunn’s original quarry. The depositional environment at this level has been interpreted as fluvial/lacustrine due to the interbedded silt and very-fine grained dark grey sandstones with the occasional ripple bed. These leaf-bearing fluvial silts and sands were deposited in a small delta atop lacustrine shales. Level B, the Wing Ding locality collected by Dunn (2003), occurs approximately between Coal 78 and 79 in The Breaks, approximately 10 m above Level A. This is the youngest site collected by Dunn (2003), and like Jingo, Wing Ding is of similar fluvial/lacustrine deposition with laminated silt and sandstones. Due to adequate museum collections from this site, no further collection was done.

The next stratigraphic level studied within The Breaks (Level C; quarries C.1 and C.2) is located between Coal 79 and 80, 151 m above Level B and is also Paleocene in age. On the mm-scale, there are alternating beds of light and dark grey very-fine grained sandstone and siltstone. Additionally, there are layers of ripples preserved throughout; these ripple beds are more prevalent than at Level A. We interpret the depositional environment as lacustrine. Plant debris, sticks, wood, and well-preserved leaf compressions are fossilized above and below the ripple beds; however, no identifiable fossils are found in ripple beds. The lacustrine deposit is capped by an orange, laterally continuous, fine-to coarse-grained sandstone.

The earliest Eocene sites collected for this study (Level D; quarries D.1, D.2, and D.3) are located in the uppermost portion of the Big Channel complex, exposed at 183 m in a section called “big channel lateral (BCL)” which is located 2.5 km to the northeast the main Big Channel section at Hanna Draw (Fig. 2). These sites are believed to occur late in the PETM because of their stratigraphic location within Big Channel and carbon isotope curves (M. Dechesne, 2019, personal communication). Exposure of fossiliferous silty sandstone is limited, and rocks are highly fractured, making excavation of fossils here challenging. D.1 consists of matted layers of fossil leaves and organic debris within fine to very fine-grained sandstone. We interpret the deposit as being a small pond, possibly an oxbow pond, due to the lithology and the presence of abundant laminated bedding planes. D.2 and D.3 occur approximately 0.5 m above D.1 and are interpreted as being fluvial in deposition, possibly representing crevasse splays. The two localities are approximately 150 m apart and occur in white, massive, fine-grained sandstone. Preservation at D is poor due to the course sediment of the fluvial system. Due to the nature of the outcrop, bench quarries were not feasible and sample sizes are small.

The highest stratigraphic level excavated (Level E; quarries E.1 and E.2) is located within The Breaks (73 m in Beer Mug Vista section, Fig. 2), approximately four km north of the latest Paleocene localities in the Beer Mug Vista section (M. Dechesne, 2019, personal communication). E.1 and E.2 (~90 m apart) are comprised of fine-grained sandstone layers bounded by coarse-grained sandstone. Fossiliferous layers were preserved as matted organic rich layers with a sharp contact to poorly consolidated, coarse-very coarse sandstone, and we interpret this stratigraphic level as fluvial in deposition. E.1 was excavated via bench quarry while E.2 was heavily fractured, allowing for large blocks to be excavated, split and analyzed on the outcrop. E.2 has the best preservation of all stratigraphic levels, including the preservation of cuticle and fourth order venation.

### Laboratory analyses

Fossils were sorted into leaf morphospecies based on the shape, marginal features, and vein characteristics described in Ellis et al. (2009). When diagnostic features were covered by matrix, fossil leaves were prepared in the University of Wyoming Geological Museum using an air scribe. Fossils leaves collected for this study were then compared to the morphotypes described by Dunn (2003). Insect herbivore diversity and frequency were quantified on all identifiable leaves, and damage morphotypes (DTs) were assigned using Labandeira et al. (2007). Herbivore damage is distinguished from preservational damage, such as tearing or decomposition after the leaf abscission, by the presence of thickened tissue around the damage site (Labandeira, 2002). Herbivory was also analyzed for Dunn’s Jingo (A.1) and Wing Ding (B) collections at the Denver Museum of Nature & Science.

We used the relationship observed in extant plant communities between the proportion of dicot species with untoothed margins at a site (P) and the MAT of the site to estimate MAT of each stratigraphic level (Eq. (1)) (Wolfe, 1979; Wilf, 1997). Equation 2 was used to calculate standard deviation of MAT where *r* is the number of species in the sample (Wilf, 1997).

(1) $${\rm MAT } = {\rm }30.6P{\rm } + {\rm }1.14$$

(2) $$\sigma \left[ {{\rm{MAT}}} \right] = 30.6\sqrt {{{P\left( {1 - P} \right)} \over r}} $$

Paleoprecipitation estimates are based on the relationship between the size of the leaves and MAP (Wilf et al., 1998). Fossil leaf sizes were documented using size classes set by Raunkiaer (1934). Using the known relationship between leaf area and MAP of modern forests (Wilf et al., 1998), MAP was determined as

(3) $${\rm{MAP}} = \;{{\rm{e}}^{0.548\left( {\sum {{a_i}} {p_i}} \right){\kern 1pt} + {\kern 1pt} 0.768}}$$

where *p_i_* is the proportion of leaves in the size classifications and *a_i_* is the natural logarithm of leaf areas of the Raunkiaer size-classes (Wilf et al., 1998). The standard error for MAP was calculated using the standard error (SE = 0.359) from Wilf et al. (1998).

Rank abundance, floral diversity indices, species richness, evenness, and overall composition were analyzed for both the 2017 collections and Dunn’s thesis data (2003). Rank abundance curves were created for each stratigraphic level representing the proportion of abundant morphospecies. We used the Shannon-Wiener diversity index, which weights species according to their frequency, to compare diversity among sites (Routledge, 1979; Iknayan et al., 2014). Rarefaction curves were created to analyze sampling effort at all stratigraphic levels and to standardize plant species richness based on sample size for comparison. Floral evenness was quantified for all stratigraphic levels using Pielou’s J (Pielou, 1966a, 1966b). Lastly, to understand how plant community composition changed across time and stratigraphic levels, non-metric multidimensional scaling (NMDS) ordinations were constructed using a matrix of floral abundances at each stratigraphic level and the function metaMDS in the package Vegan (Oksanen et al., 2018). All NMDS analyses were run using the Euclidean distance metric on count data. All analyses were conducted using the R platform (R Core Team, 2018) version 3.5.1, and all R code is included in Appendix A3.

Herbivory was compared among stratigraphic levels by analyzing damage diversity, frequency of herbivory and damage composition. Damage diversity is the number of DTs observed, and it is heavily dependent on sample size; thus, we standardized by the number of leaves analyzed (Schachat, Labandeira & Maccracken, 2018). Rarefactions were calculated using Gunkel & Wappler’s (2015) methodology, which accounts for leaves with no damage and leaves with multiple DTs. Rarefaction curves were generated for the total number of DTs and specialized feeding DTs on the bulk flora at each stratigraphic level, as well as for the total number of DTs on individual plant hosts with at least 20 leaves within a stratigraphic level. Generalized feeding insect herbivores are non-selective of host plants, whereas specialized feeding insects selectively feed on specific plant taxa. Specialized herbivores have adapted or evolved to the plant defenses of their host taxon, illustrating coevolution between plant and insect communities (Krieger, Feeny & Wilkinson, 1971; Whittaker & Feeny, 1971). Specialized feeding is recognized in the fossil record when the morphology of damage is consistent with that of known specialized feeders (e.g., miners and gallers) (Labandeira, 2002). Damage frequency was analyzed as the percent of leaves in a sample with any, specialized or generalized damage. NMDS ordinations were made to evaluate changes in herbivore damage composition across stratigraphic levels utilizing a matrix of damage abundances and function metaMDS, Euclidean distances and count data.

## Results

A total of 65 distinct plant morphospecies were identified across the five stratigraphic levels. 21 morphospecies occur in the 2017 collections, and 44 morphospecies were part of Dunn’s MS thesis collections. Eight morphospecies are shared across the 2003 and 2017 collections. Descriptions of each morphospecies from the 2017 collections are included in Appendix A4, summary tables of floral occurrences for each locality are listed in Appendix A5 and the full census dataset is included in Appendix A6. Using the list of morphospecies and their characteristics, MAT and MAP reconstructions for each stratigraphic level were calculated (Table 1). MAT estimates range from 18.8 ± 2.6 °C (Level B) to 22.3 ± 3.9 °C (Level C). MAP (cm/year) estimates range from 108 + 46.9, −32.7 (Level D) to 184.0 + 79.3, −55.4 (Level B). Due to large error estimates and low species diversity, climate reconstructions are not meaningfully different across stratigraphic levels. Continued sampling and the discovery of new species are necessary to further refine climate reconstructions.

**Figure 4:**
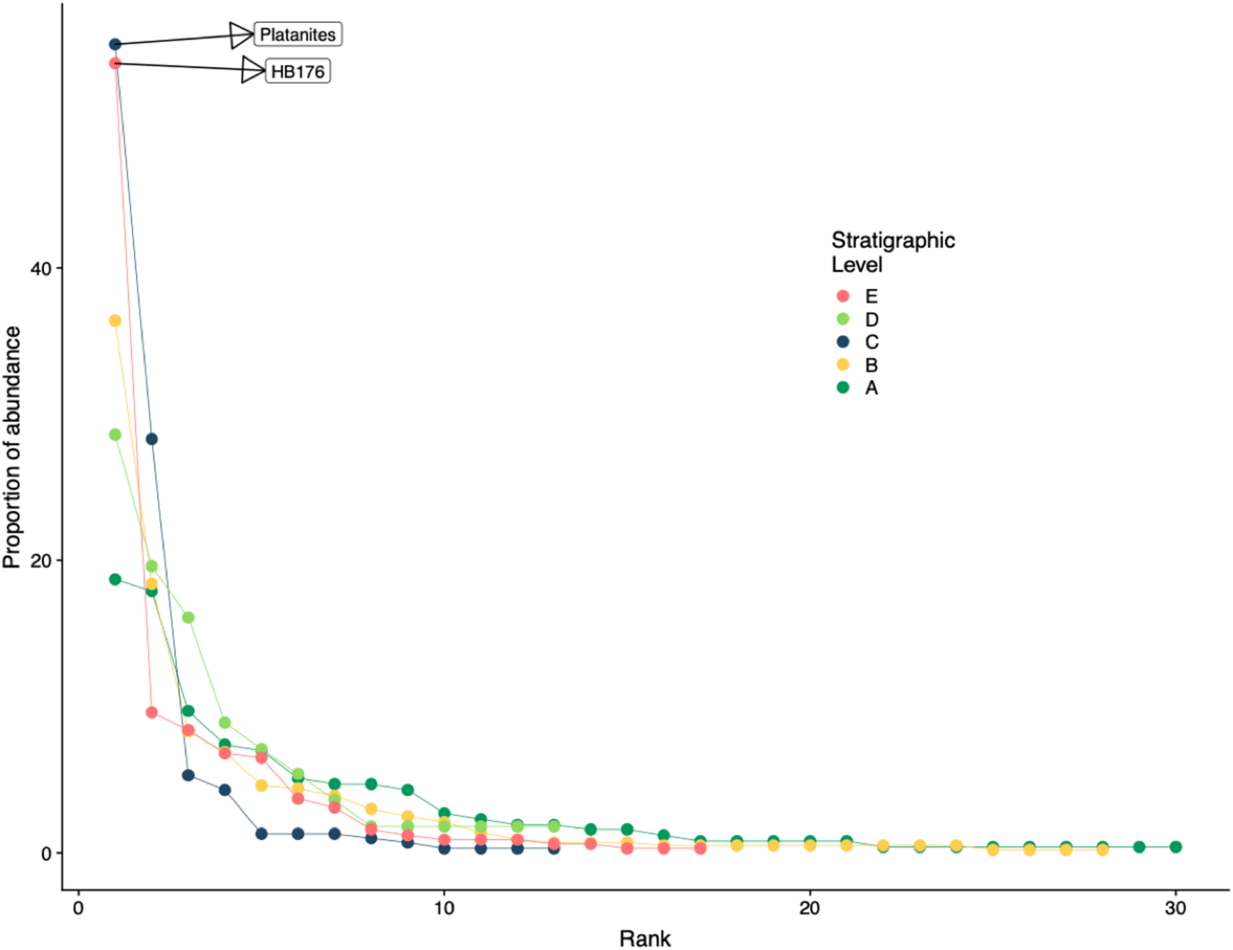
Rank abundance curve for all stratigraphic levels. Rank abundance curves for plant species at each stratigraphic level. Morphospecies with at least 45 specimens are labeled.

Floral rank abundance results are plotted for each stratigraphic level (Fig. 4) and show that Levels C and E are heavily dominated by one or two taxa. Site A.1 (Dunn, 2003) has 21 morphospecies, two of which make up ~40% of all specimens, HB077 (21.6%) and HB104 (20.7%). Site A.2 contained 14 morphospecies, and 20% of specimens are morphospecies HB176 (Fig. 3C). Level B has a total of 33 morphospecies, and 40.6% of specimens are morphospecies *Averrhoites affinis* (Fig. 3F). At stratigraphic level C, a total of 306 fossil leaves were analyzed and 14 morphospecies identified. C.1 is comprised of 12 different morphospecies, with *Platanites raynoldsii* (Fig. 3D) making up 59.1% of specimens. C.2 has 10 different morphospecies, and the two morphospecies with the highest relative abundance were *Trochodendroides genetrix* (Fig. 3B) at 43.4% and *P. raynoldsii* (Fig. 3D) at 42.1%. Stratigraphic level D has a total of 10 morphospecies. Morphospecies HB176 (Fig. 3C) is the most abundant leaf type at D.2 and D.3, whereas D.1 is dominated by HB180 (Fig. 3G) (27.5%) and HB176 (Fig. 3C) (22.5%). Stratigraphic level E has a total of 19 morphospecies. At both E.1 and E.2, morphospecies HB176 (Fig. 3C) had the highest relative abundance (30.8% and 57.9%, respectively).

**Figure 5:**
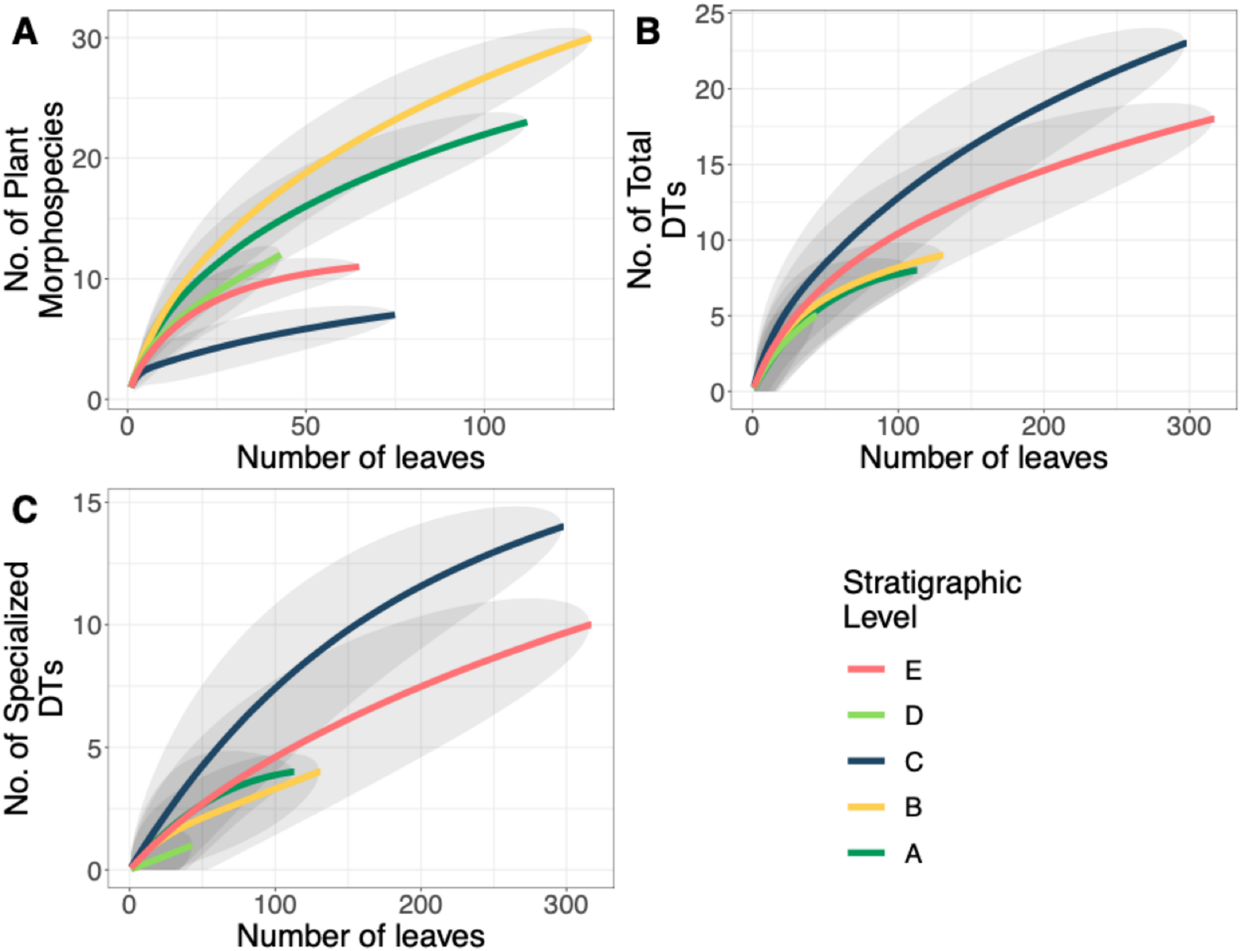
Plant and insect herbivore rarefaction curves. (A) Resampling curves of floral diversity, (B) total damage diversity on the bulk flora, and (C) specialized damage diversity on the bulk flora at each stratigraphic level. Gray ovals represent upper and lower confidence intervals (95%) for each stratigraphic level. DT = damage morphotype.

Floral diversity indices and evenness (*J*) values are reported in Table 2 for individual quarries and stratigraphic levels. Considering quarries with at least 40 specimens, E.1 has both the highest diversity and is the most even, compared to C.1 which has the lowest diversity and evenness. Various other quarries show a discrepancy between diversity and evenness which is likely due to sample size when dividing out each specific quarry. By binning individual quarries together and analyzing the stratigraphic levels for Shannon diversity (*H*; dominant and rare species weighted similarly), we see that Level A has the highest diversity (*H* = 2.7), and the lowest diversity is at Level C (*H* = 1.30) along with the lowest evenness (*J* = 0.51). The stratigraphic level with the highest evenness is Level D (*J* = 0.82). It is important to note that both C and E have low diversity and evenness values, while stratigraphic Levels A and D have similarly high diversity and evenness estimates. Floristic rarefaction curves for each stratigraphic level show that our sampling effort was insufficient in fully capturing floral richness at the sites as none of the curves reaches a sampling saturation plateau; additional collections are required to determine the true richness of the ecosystem (Fig. 5A). Levels A and B have the highest plant richness with Levels C and E having the lowest. Results from Level D should be interpreted with caution because of the low sample size; however, due to the location of Level D within the late PETM we report the results.

**Table 2:** Diversity indices table. Diversity index and evenness metric for each quarry and stratigraphic level. Values calculated by Dunn (2003) denoted with a superscript of (*). For stratigraphic level A, data was pooled from Dunn’s (2003) work and our 2017 collections.

	LATE PALEOCENE					PETM			EARLY EOCENE	
	A.1*	A.2	B*	C.1	C.2	D.1	D.2	D.3	E.1	E.2
Shannon diversity (quarry)	2.06*	2.03	2.24*	1.26	1.26	1.33	1.94	1.54	1.93	1.53
Shannon diversity (strat. level)	2.70		2.24*	1.30		2.08			1.72	
Pielou’s J (quarry)	0.73*	0.79	0.67*	0.51	0.55	0.96	.84	0.86	0.78	0.55
Pielou’s J (strat. level)	0.80		0.67*	0.51		0.82			0.61	

**Figure 6:**
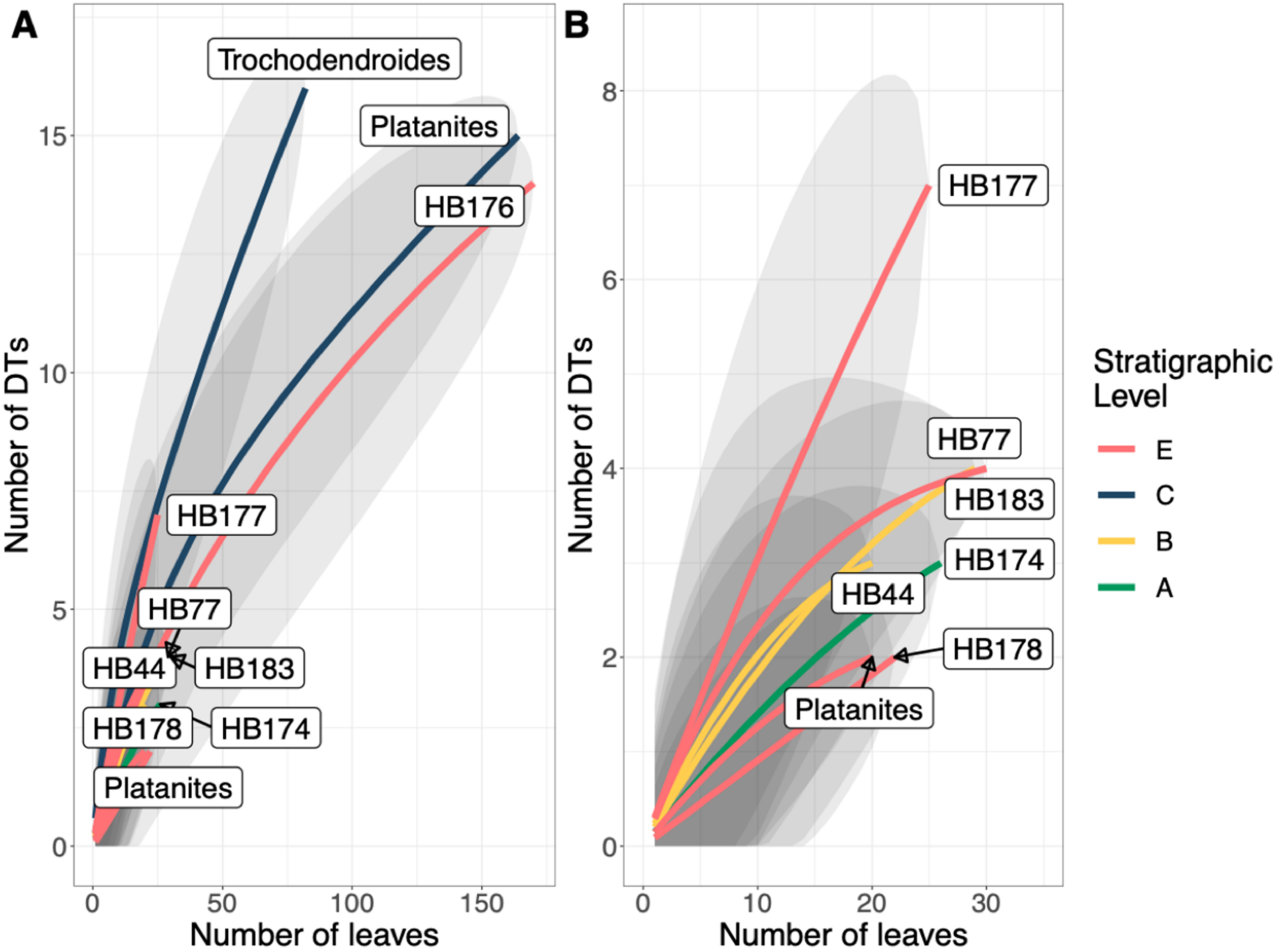
Resampling curves of damage type (DT) richness on individual plant hosts that have 20 or more leaves at a single stratigraphic level. (B) is an enlargement of the lower left corner of (A). *Platanites raynoldsii* is the only species that occurred in high enough abundance at two stratigraphic levels (Levels C and E) to be able to compare damage diversity on a host plant. Gray ovals represent upper and lower confidence intervals (95%) for each stratigraphic level.

A total of 34 DTs were recognized in this study. Herbivory rarefaction curves plot DT richness standardized by number of leaves analyzed (Figs. 5B and 5C). All plots show that additional sampling is needed to capture the diversity of herbivory in each ecosystem as well. Levels C and E have the highest richness for both total (Fig. 5B) and specialized damage (Fig. 5C), despite having low plant species richness. In contrast, Levels A, B, and D have lower total and specialized damage richness, although additional sampling at Level D is necessary to confirm this result. Damage richness for all morphospecies with at least 20 leaves in a stratigraphic level were plotted (Fig. 6). Only Levels C and E shared a plant morphospecies (*P. raynoldsii*) that occurred at high enough abundance to analyze, and damage richness in Level C is higher than in Level E (Fig. 6).

**Figure 7:**
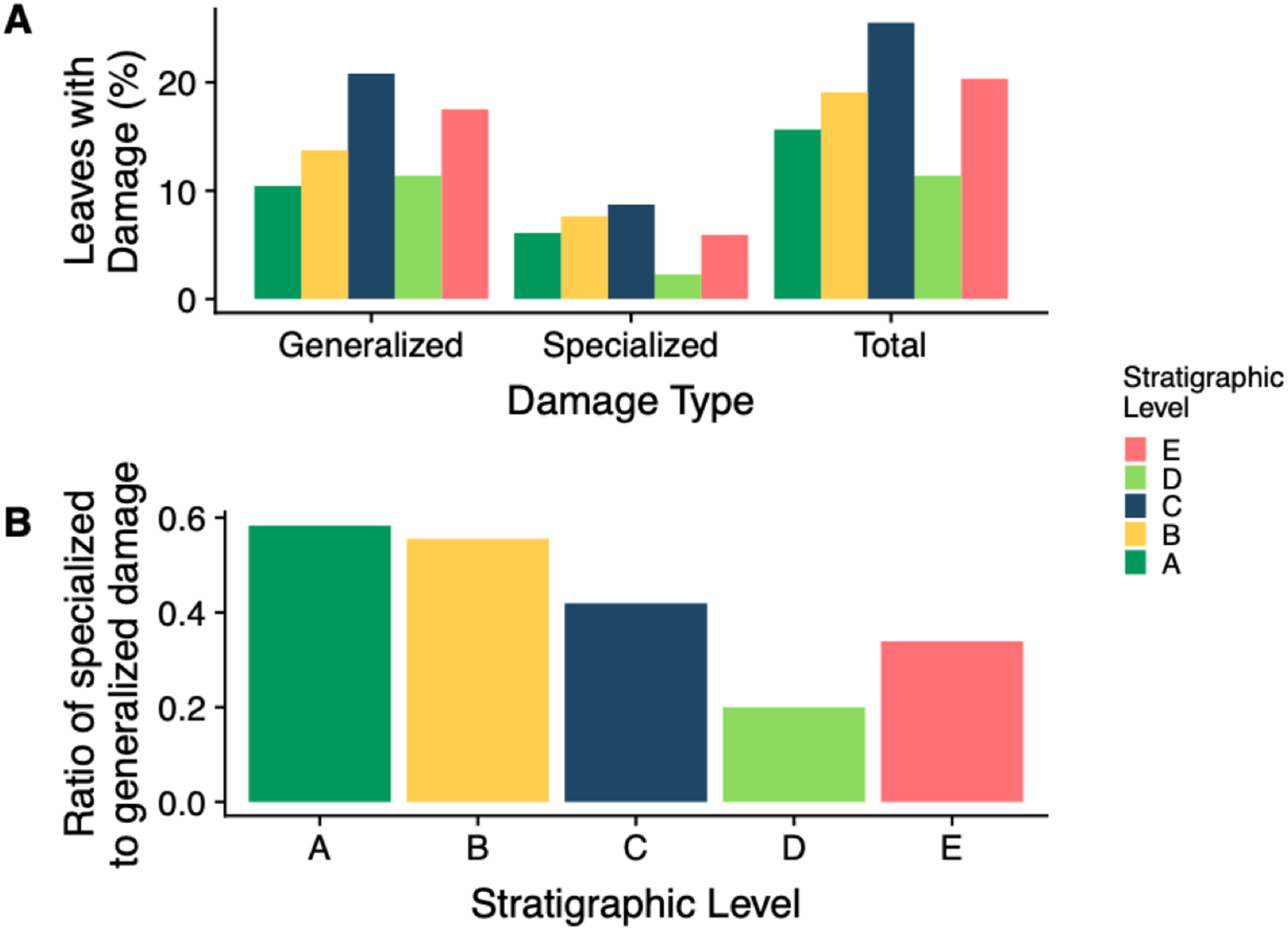
Insect damage frequency and ratio of specialized to general damage. (A) Percent of leaves at each stratigraphic level with generalized, specialized and total insect herbivore damage. (B) The ratio of specialized feeding to generalized feeding at each stratigraphic level

The stratigraphic level with the highest frequency of herbivory is Level C (25.5%) and the lowest is Level D (11.4%) (Fig. 7A). Across all stratigraphic levels, generalist feeding damage is more abundant than specialist feeding damage. Specialized feeding is the most frequent at Level C and least frequent at Level D, however increased sample size at Level D is needed to confirm this result. The ratio of specialized feeding to generalized feeding informs about the prevalence and relative importance of specialist feeders within the insect herbivore community. Levels A and B have the highest ratio of specialized to generalized damage across stratigraphic levels, and Level D has the lowest ratio (Fig. 7B).

**Figure 8:**
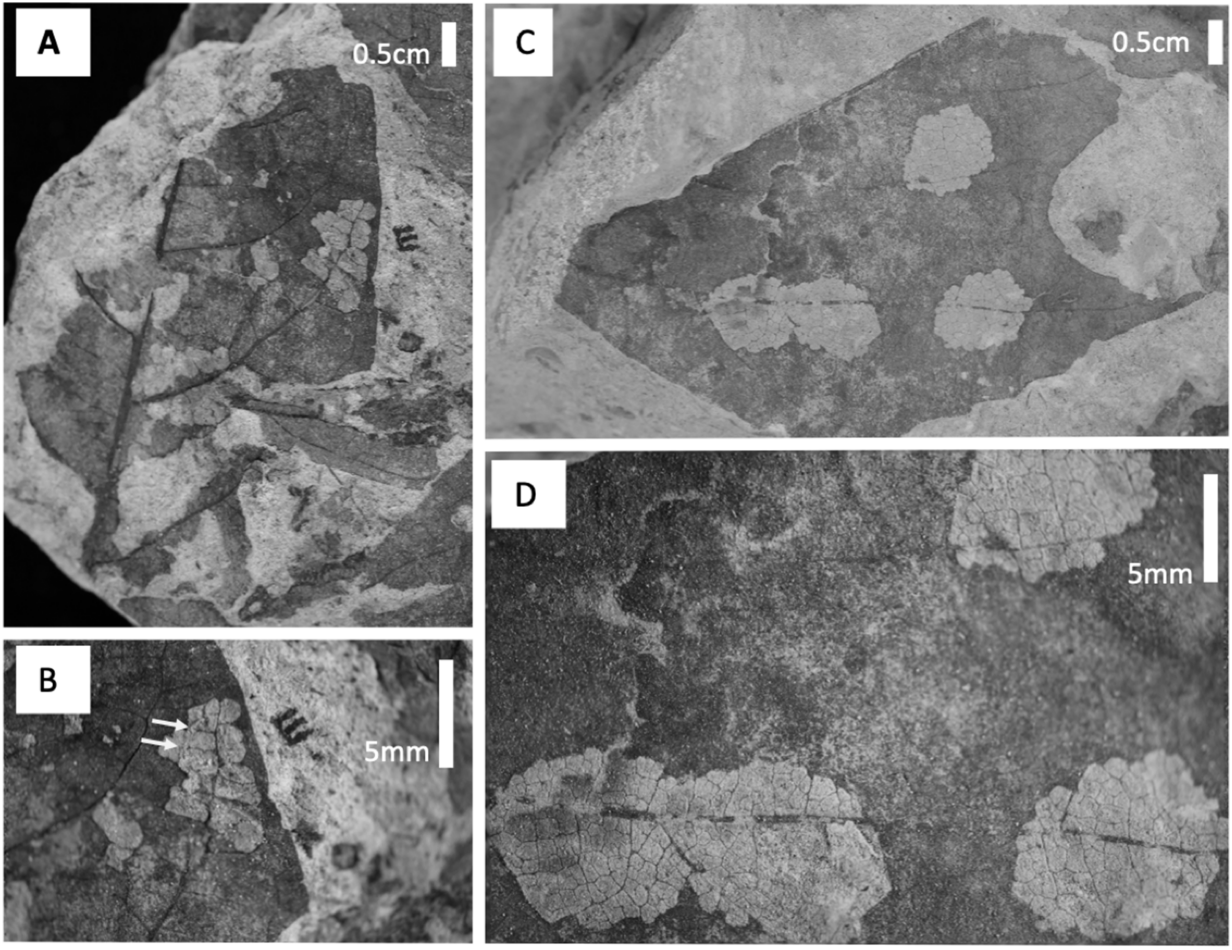
Damage type photos. (A) HB177 (entire leaf) with damage (locality E, specimen #PB976). (B) Close up image of A with damage believed to be caused by extant beetle family, Chrysomelid. (C) Damage on *Platanites raynoldsii* with DT38 (locality C, specimen #PB922). (D) Close up of DT38.

A particular type of mining, DT38 (Fig. 8), occurs at Level C on *P. raynoldsii* and is characterized by numerous circular areas that resemble skeletonization. Today, similar damage traces are made by members of the family *Incurvariidae* (Lepidoptera; CC Labandeira, 2017, personal communication). Another distinctive damage morphology, found on morphospecies HB177 at site E.2, is similar to damage by the extant Chrysomelid beetle *Odontota dorsalis*, known to feed on secondary veins (S. Shell, 2017, personal communication). This damage is a specific type of skeletonization, in which the secondary veins are thickened at the ends, suggesting that they were fed on (Fig. 8). Unlike other types of skeletonization, where only the intercostal areas are fed on, the veins on this fossil have also been eaten, a damage pattern that is rare for skeletonizing insects because chemical defense is distributed via venation (Caldwell, Read & Sanson, 2016).

**Figure 9:**
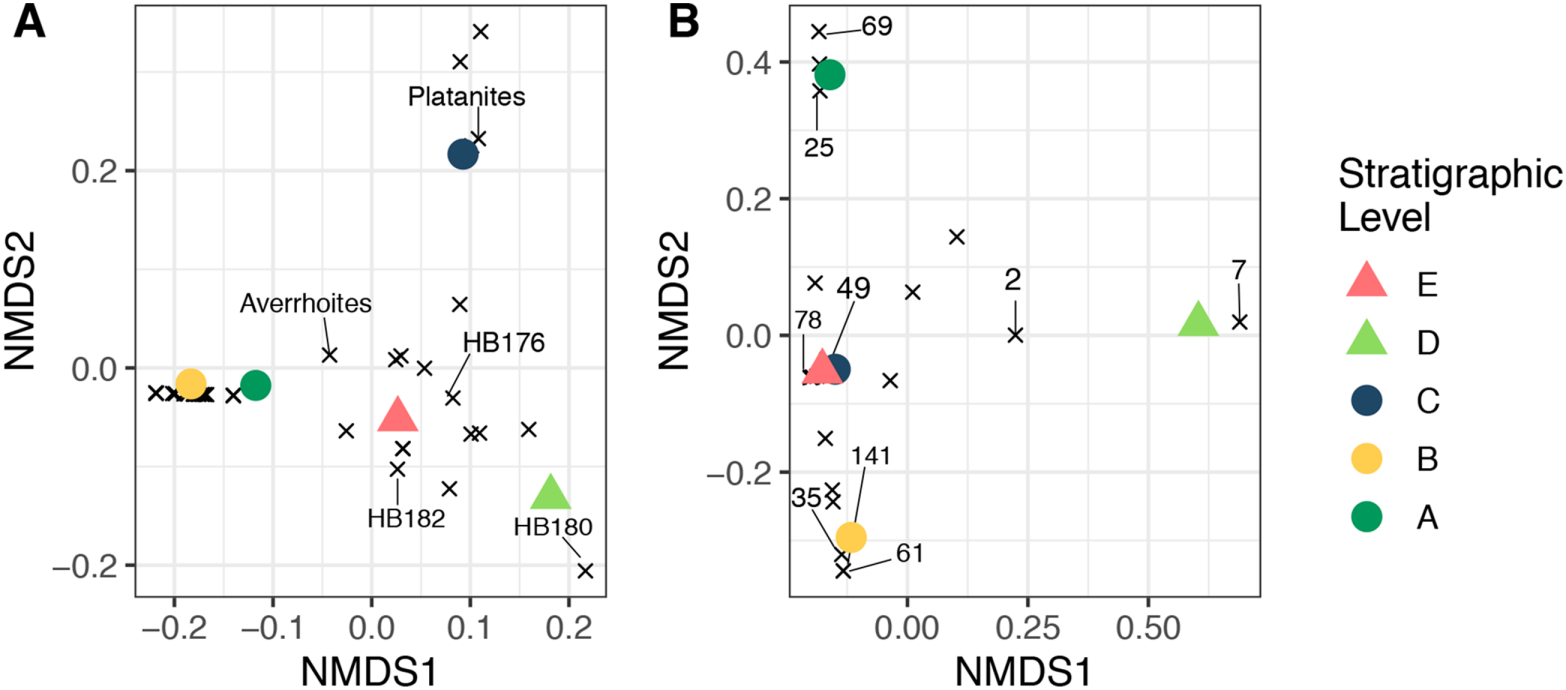
Nonmetric multidimensional scaling ordination. Nonmetric multidimensional scaling ordination of floral composition (A) and insect herbivory on the bulk floras (B) across all stratigraphic levels. The “×” symbols in A represent plant morphospecies and in B represent insect herbivore damage types (DTs), labeled using the numbers of Labandeira et al. (2007). These are plotted to show their impact on the placement of each stratigraphic level. The unlabeled “×” symbols affect the placement of the samples in the ordination, but for simplicity, only morphospecies or DTs that are abundant and explain separation of samples were labeled. Stress <0.01 for both plots.

Non-metric multidimensional scaling ordinations of plant and herbivore community structure are shown in Fig. 9. Considering plant community structure (Fig. 9A), Levels A and B plot near one another in the lower left corner, with low scores on both axis 1 and axis 2. Level C has a similar axis 1 score, but a high axis 2 score and plots at the top of the figure. This position is controlled by the abundance of *P. raynoldsii*. Level E has a similar axis 1 score as Level C, but scores lower on axis 2. This location is dictated by HB176, HB182, and *A. affinis*. Stratigraphic Level D has a high axis 1 score and is the only site that plots on the right side of the ordination. The location of Level D is controlled by the morphospecies HB180 (Fig. 3G). For herbivore community structure (Fig. 9B), Levels A and B plot in distinct areas of the ordination, while Levels E and C plot almost on top of one another. As in the plant community structure NMDS, Level D is alone on the right-hand side of the graph. Influential damage types are labeled in Fig. 9B to visualize which DTs most influence the placement of stratigraphic levels in the ordination. DT7 (hole feeding: curvilinear to rectilinear elongate perforations) drives the placement of stratigraphic Level D on the right of the ordination, whereas DT69 (mining: circular to ellipsoidal with coprolites), 25 (surface feeding: elongate, narrow surface feeding with constant width), 141 (mining: thin zigzag with frass), and 61 (skeletonization: elongate and adjoined that follows 1° and 2° venation) are responsible for the distance between Levels A and B. DT 49 (galling: circular, large fusanized core surrounded by distinct outer thickened rim) and 78 (hole feeding: removal of tissue in three or more intercostal sections) influence the proximity of C and E.

## Discussion

This study documents how Hanna Basin plant and insect communities changed across an approximate three-million-year interval that spans the Paleocene–Eocene boundary. Here, we examine potential abiotic and biotic factors driving the changes observed, including depositional environment, water availability, the PETM, and co-occurrence of plants and insect herbivores. It is important to note, as discussed in the Results section, that additional sampling is needed to capture the full diversity of the ecosystems and provide more robust reconstructions of paleoclimate and paleoecology.

The dominant depositional environment in our study system varies both temporally and spatially across the landscape, which may have affected the composition and dominance-diversity patterns in plant fossil assemblages. The ecosystems preserved within Levels A, B, and C are fluvio-lacustrine or lacustrine in nature while, Levels D and E were deposited in a strictly fluvial system. Plants are relatively immobile and unable to move their physical location once germination has occurred, and it is likely that small changes in depositional environment would impact a community. As depositional environment changes, plant communities are at risk of losing land for colonization, becoming isolated from resources and potentially becoming more vulnerable to disturbances (Nilsson & Svedmark, 2002). Changes in deposition can also alter water availability and soil moisture content, reshuffling niche space on the landscape (Jorge Soberón, 2007; Guisan et al., 2014). Lacustrine and fluvial environments differ in taphonomic biases through varying levels of transport; lacustrine facies experience less transport than fluvial environments which would impact the amount of autochthonous vs. allochthonous material (Ellis & Johnson, 2013). Fluvial environments (allochthonous) tend to be more diverse because they contain a mix of local and transported leaves and thus capture a greater source area; however, with increased transport large leaves are also less abundant which biases our record toward smaller leaves thus impacting precipitation reconstructions (Ellis & Johnson, 2013).

Lithology and sedimentary structures at Levels A and B indicate a very similar depositional environment, which we interpret as terminal splays draining into a paleo-lake. Plant composition at these sites are compositionally quite similar, as indicated by the NMDS ordination. This similarity may also be a result of a mixture of allochthonous and autochthonous material washed into the paleo-lake. The size and preservation of the leaves at both sites vary, as might be expected in mixed source material. Between Level B and Level C, lake size and depth increase, as indicated by changes in regional stratigraphy as well as local changes upward into a siltstone and less ripple bedded sandstones at our Level C quarries. Plant composition at Level C is significantly different, with low diversity and high dominance of *P. raynoldsii*. This could be due to less land surface in the basin for species to colonize (Odum, 1969). Alternatively, because Level C is likely a less dynamic environment than Levels A and B, the leaves preserved here reflect less transport and a more localized environment. Lastly, the low plant diversity could also be a reflection of preservational bias toward thicker, tougher leaves (e.g., *P. raynoldsii*).

Concomitant with a change from a shallow lacustrine system to a predominantly fluvial system is the PETM hyperthermal event as determined by carbon isotope stratigraphy (M. Dechesne, 2019, personal communication). Elsewhere, this climatic event coincides with increased rates of weathering and continental runoff (McInerney & Wing, 2011; Foreman, Heller & Clementz, 2012). It has been hypothesized that increased runoff could be due to (1) increased transpiration from plant communities (Betts et al., 2007), or (2) enhanced seasonality which may have caused opening of forest canopies (Foreman, Heller & Clementz, 2012). Either mechanism could explain the formation of the stacked sand bodies of the Big Channel complex during the PETM in the Hanna Basin. These hydrological changes and rise in global temperature may have impacted both plant and insect communities. Plant species and insect herbivory preserved within Level D, which is at the top of the Big Channel sequence, late in the PETM interval, shows a significant turnover compared to pre- and post-PETM communities (Fig. 9), a result consistent with the Bighorn Basin and other PETM floral records (Wing & Currano, 2013). The increase in sediment flow would have altered the landscape and impacted floral communities within.

In contrast to the Paleocene levels, the dominant depositional environment at Levels D and E is fluvial; however, they differ slightly in their lithology and plant and insect communities. The structures and stratigraphy of Level D suggest that the channel cut across the floodplain of a wetter environment; additionally, the irregular bases of the beds suggest the sediment load deformed the already wet bed that preceded it. Due to poor preservation, highly varied leaf sizes and lithology, the leaves preserved at this locality are likely a mix of autochthonous and allochthonous material. The larger leaves preserved at this site may be sourced locally whereas the smaller and more poorly preserved leaves may have traveled a longer distance. Additionally, the leaves preserved at this site are of poor quality because they are found within the sandstone of BCL. Unlike the crevasse splay deposits of Level D, Level E is interpreted as being proximal to the stream due to the sharp contacts between coarse-very course grained sand and organic rich layers. Sediment load was highly variable and would have rapidly fluctuated between higher and lower flow conditions (De Fátima Rossetti, 2001). Leaves preserved within Level E are large and exceptionally well-preserved, suggesting minimal transport. These two sites (Levels D & E) may vary in composition due to the PETM event, but there is also a vastly different energy regime at either site, impacting the preservation.

When comparing the lacustrine deposit of Level C and the fluvial deposit of Level E, we see that both are dominated by a single plant species (*P. raynoldsii* at Level C and HB176 at Level E) and have the highest diversity of insect herbivory. As lake levels rose, the available land for colonization decreased at Level C. This would have also been true for the location of Level E; with frequent flooding events, plants would have had limited area to colonize within this highly varied system. The decrease in evenness at these sites may also be coupled with the higher insect herbivory observed. Together, these two localities could give a more complete representation of the paleo ecosystem within the Hanna Basin as they represent two-end members of energy found within the system: the low energy of Level C with its finer grained sediment and less frequent ripple beds, and a high energy flood system of Level E with its sharp contact between course sediment and organic rich leaf layers.

The variability among all stratigraphic levels suggests that changes in depositional environment did impact the plant and insect herbivore communities. Although we do not see a clean signal of lacustrine vs. fluvial deposits, this is not surprising as all five localities are highly varied in the finer details of their deposition. It is possible that the species:area relationship coupled with depositional environment, as seen when comparing Levels C and E, may show similar patterns of plant occurrences and herbivory, even though the plant taxa are different.

Insect herbivores are highly influenced by changes in plant communities (Ali & Agrawal, 2012; Richards et al., 2015), plant nutrient content (Coley, Bryant & Chapin, 1985; Onoda et al., 2011), and temperature (Jamieson et al., 2012). Indirectly, insect communities are impacted by changes in atmospheric CO_2_ via changes in plant nutrients (Dury et al., 1998; Knepp et al., 2005; Dyer et al., 2013). Our analyses show that insect damage diversity does not track plant diversity (Fig. 5), contrary to expectations that as plant diversity increases, potential food sources might also be expected to increase. Levels A and B have high plant richness but low total and specialized damage diversity whereas Level C has low plant species richness but the highest diversity and frequency of total and specialized damage. This is somewhat surprising result is counter to modern studies linking high plant diversity to high insect herbivore diversity (Ebeling et al., 2018; Schuldt et al., 2019), but is consistent with what Currano, Labandeira & Wilf (2010) reported for the Paleogene in the Bighorn Basin. There are several reasons why plant and insect damage diversity are uncorrelated in our dataset. First, Level C has the lowest floral evenness and is dominated by *P. raynoldsii*, a species that is common across the western USA during the early Paleogene. According to Feeny’s (1976) apparency hypothesis, plant taxa that are regionally dominant are “bound to be found” by insect herbivores; they are apparent and, simply put, more likely to be fed on. Additionally, there is incentive for insect species to evolve means to overcome the defenses of apparent plant species and become specialist feeders on that particular host. *P. raynoldsii* is heavily fed upon at Level C (Fig. 6), where it is the most abundant plant host, and there are several examples of specialized feeding damage on this taxon (Fig. 8). Alternatively, canopy structure, light availability and disturbance regime have been shown to influence insect diversity, particularly in the Heteroptera and Coleoptera (Gobner, 2009; Currano et al., 2011; Schroeder, Buddle & Saint-germain, 2019). These environmental variables are difficult to reconstruct from fossils, and it is possible that variation among stratigraphic levels affected insect herbivore diversity. Our work provides further evidence that the relationship between plant diversity and insect herbivore diversity in both modern and ancient ecosystems is variable and may be highly contingent on specific habitats (Wright & Samways, 1998; Wilf et al., 2006; Proches et al., 2009; Schuldt et al., 2019).

The percent of leaves with generalized, specialized, and total feeding damage is varied across stratigraphic levels with Level C having the highest percentage of all three (Fig. 7A). Similarly, Level E has the second highest percentage of all three damage categories. This is reasonable because as we previously saw, Levels C and E had the highest total and specialized damage diversity; however, what is particularly interesting is the ratio of specialized feeding to generalized feeding is higher at Levels A and B (Fig. 7B). As levels of plant diversity increased at Levels A and B, we see a higher frequency of specialized to generalized feeding. This suggests that although overall plant and insect community structure do not track one another, functional feeding groups may. With more diverse plant species, there is an added benefit for insects to specialize in order to mitigate ever evolving plant defenses. These findings contradict Feeny’s apparency theory. Level D has the lowest frequency of total and specialized herbivory, a surprising result because damage frequency and percent leaf area damage increase in the Bighorn Basin during the PETM (Currano et al., 2008, 2016). New PETM sites are needed to confirm this result.

Similarly, changes in insect damage composition appear to be largely independent of changes in plant composition. Plant ordinations show that Levels A and B plot near one another while the same localities are dissimilar in ordination space when comparing herbivory (Fig. 9). Levels C and E are very similar in terms of herbivory but not floral composition. In contrast, both floral and herbivore composition at Level D are distinct when compared to sites before and after. Insects experienced a significant turnover during the PETM (Level D), mirroring plant community change, however they returned to a very similar community structure as seen prior to the carbon isotope excursion. Floral and insect herbivore turnover during and after the PETM has also been reported in the Bighorn Basin and was attributed to an increase in temperature, shift to seasonally dry conditions, and changes in plant nutritional quality (Currano, Labandeira & Wilf, 2010; Wing & Currano, 2013; Currano et al., 2016). Discovery of new paleobotanical localities in the Hanna Basin is necessary to increase our sample size and number plant species recovered, improve estimates of MAT and MAP change (or lack thereof) during the PETM and determine whether climatic forcing drove changes to plant and insect herbivore communities, as it did in the Bighorn Basin. Alternatively, the biota may be tracking changes in nutrient cycling or changes to another limiting factor not quantified here. Previous studies have shown that stressed plants increase nitrogen flow, resulting in higher levels of nitrogen within leaves (White, 1984). This relationship between nitrogen, water availability, and stress could explain why plant and herbivore communities track one another during times of disturbance, specifically Level D which occurs late in the PETM.

When comparing the Hanna Basin to the Bighorn Basin, one distinction between the two basins is the difference in water availability, indicated by the abundance of coal and carbonaceous shales in the Hanna Fm. Although carbonaceous shale deposits are known from the contemporaneous Willwood Fm. of the Bighorn Basin (Kraus & Gwinn, 1997; Davies-Vollum & Kraus, 2001), the magnitude of these deposits is significantly less than within the Hanna Basin. Additionally, the well-developed paleosols of the Bighorn Basin suggest drying during the PETM (Kraus, 1996; Kraus & Riggins, 2007). Based on lithology alone, it is reasonable to assume that the plant and animal communities within the Hanna Basin had more available water when compared to the Bighorn Basin. Modern wetland studies have shown how plant physiological processes respond to changes in water availability and the overall effects of drought tolerance (Touchette et al., 2007; Erwin, 2009; Nardini & Luglio, 2014). Plants experiencing drought are highly susceptible to predation, desiccation, and mortality (Breshears et al., 2009; McDowell, 2011; Meineke & Frank, 2018); thus it is reasonable to infer that plants with ample available water could more effectively mitigate insect herbivore attacks during abrupt climatic changes (Meineke & Frank, 2018). As leaf venation and morphologic traits are tied to water availability (Xu et al., 2009), it is logical to conclude that with high water availability the morphologic signal these species are preserving is not truly related to climate but rather the available water supply, dictated by the composition of soils. More research is needed to better understand plant responses to high water availably as atmospheric CO_2_ and temperature changes, and ultimately, how this impacts plant defenses against herbivory (Onoda et al., 2011; Richards et al., 2015).

## Conclusions

In order to systematically understand our future, we must document background changes in ecosystem function as anthropogenic climate change ramps up over the next century. The paleoenvironments preserved within the Hanna Basin allow for a comparison of changes to floral and insect herbivore communities during a time of highly variable global climate. In this study we found that plant and insect herbivore diversity, along with community composition, did not track one another; however, changes in both plant and insect community composition and structure coincide with the PETM event. Additionally, we found that generalized feeding was more abundant than specialized feeding, and we did not see an increase in damage frequency or diversity during the PETM interval. This may be due to taphonomic and preservational constraints, and the discovery of new PETM localities is needed to corroborate these findings. The plant and insect communities preserved within the Hanna Basin had a unique response to the PETM when compared to the Bighorn Basin WY. Much previous research has focused around understanding how temperature and CO_2_ impact flora and fauna on a landscape, but as suggested here, other abiotic factors such as water availability and depositional setting are also important factors to consider as we push forward in the Anthropocene.

## Supplemental Information

10.7717/peerj.7798/supp-1 Supplemental Information 1 Plant and insect data for each quarry. Raw data used to analyze plant and insect communities per quarry or site.

10.7717/peerj.7798/supp-2 Supplemental Information 2 Plant and insect data for each stratigraphic level. Raw data used to analyze plant and insect communities per each stratigraphic level.

10.7717/peerj.7798/supp-3 Supplemental Information 3 R code for plant and insect herbivore analysis. R code used to produce analyses.

10.7717/peerj.7798/supp-4 Supplemental Information 4 Morphotype sheet with corresponding photo. Each leaf morphotype collected for this study has been described and photographed.

10.7717/peerj.7798/supp-5 Supplemental Information 5 Summary data tables. Summary data tables for each quarry described in this study. Previously collected quarries, Dunn, 2003, are also summarized here. Table (*K*) represents the distribution of morphotypes across all stratigraphic levels.

10.7717/peerj.7798/supp-6 Supplemental Information 6 Leaf and insect damage dataset. Full dataset of all leaves collected for this study including: field number, UW collections number, site (quarry location), size classification, species (morphospecies), and presence/absence of DT.

10.7717/peerj.7798/supp-7 Supplemental Information 7 Denver Museum of Nature and Science collection information. Dataset with all specimen information for the Jingo, referred to in text as level A.1 and Wing Ding (WD), referred to in text as level B quarries.

## Additional Information and Declarations

### Field Study Permissions

The following information was supplied relating to field study approvals (i.e., approving body and any reference numbers): Field collections for levels A, B, C and E were completed on private land with authorization from Burt and Kay Lynn Palm. Field collections for level D was completed on private land with authorization of Tad Anderson from Q Creek Ranch.

### Data Availability

The following information was supplied regarding data availability: The raw data is available in Appendix A1 and A2. All data provided is count data. Appendix A1 reports each plant morphospecies per quarry while Appendix A2 bins data into significant stratigraphic levels. Full R code is available in Appendix A3. Full morphotype sheets with photos is available in Appendix A4. Summary data tables for each individual quarry can be found in Appendix A5 with table A11 representing the distribution of morphotypes across stratigraphic levels. Specimen information for 2017 collections can be found in Appendix A6 with University of Wyoming Geologic Museum accession numbers provided for specimens that have been cataloged. Denver Museum of Nature and Science specimens provided in Appendix A7 with specimens being housed at the collections facility in Denver, CO. The authors request to be notified if these datasets are to be used in any publication and also request to be recognized accordingly.
