# Supplementary material for: Plant and insect herbivore community variation across the Paleocene–Eocene boundary in the Hanna Basin, southeastern Wyoming
Source: PeerJ. 2019 Oct 15;7:e7798. doi: 10.7717/peerj.7798 (PMC6798869; doi:10.7717/peerj.7798)
Supplement: Supplemental Information 4 — Each leaf morphotype collected for this study has been described and photographed. [file peerj-07-7798-s004.pdf]

MORPHOTYPE		NAME	*Alnus sp.*

GENERAL

MAJOR GROUP DIC **INFERED FAMILY** Betulaceae **ORGAN TYPE** Leaf

EXEMPLAR LOC. HB-1704 **EXEMPLAR** EC1518A **OTHERS LOC.**

DIAGNOSTIC FEATURES OF MORPHOTYPE

The teeth distinguish Alnus sp. from other morphospecies. Unlike other toothed specimens found within the basin, Alnus sp. has two orders of teeth. Also, the spacing of the teeth is irregular with approximately 4 teeth/cm.

MQI:

LEAF ATTACHMENT, ORGANIZATION, SIZE, SHAPE AND PETIOLE FEATURES

**LEAF ATTACHMENT:**	Not Preserved	**LEAFLET ORGANIZATION:**	Not Preserved
**LEAF ARRANGEMENT:**	Not Preserved	**LEAFLET ATTACHMENT:**	np
**LEAF ORGANIZATION:**	Not Preserved	**PETIOLE ATTACHMENT.:**	Not Preserved
**BLADE SIZE:**	Microphyll	**PETIOLE BASE:**	Not Preserved
**BLADE SHAPE:**	Ovate	**PETIOLE GLANDS:.**	None
**BLADE RATIO L:W:**	2:1	**PETIOLE X-SECTION:**	Not Preserved
**LOBATION:**	Unlobed	**MARGIN TYPE**	Serrate
**BASE ASYMMETRY:**	Symmetrical	**MEDIAL SYMMETRY:**	Asymetrical

**BASE SHAPE:**	Not Preserved	**APEX SHAPE:**	Not preserved	**Special Margin Features:**	Not Preserved
**BASE ANGLE:**	Not Preserved	**APEX ANGLE:**	Not preserved	**Terminal apex features:**	Not Preserved

PRIMARY & SECONDARY VEIN FEATURES

NAKED BASAL VEINS: Not Preserved

**PRIMARY VENATION:**	Pinnate	**N° BASAL VEINS:**	1 **Interior 2°** Absent
**MAJOR 2° Framework:**	Craspedodromous	**MINOR-2° Course:**	
**MAJOR 2° Attachment:**	Decurrent	**MAJOR 2° VEIN ANGLE:**	Smoothly increasing toward base
**MAJOR 2° SPACING:**	Irregular	**AGROPHIC VEINS:**	Absent
**INTER-2° proximal course:**		**INTER-2° VEIN LENGTH:**	
**INTER-2° distal course:**		**INTER-2° FREQUENCY:**	

TERTIARY VEIN FEATURES

**INTERCOSTAL 3°:**	Opposite percurrent - straight	**PERIMARGINAL VEINS:**	None
**EPIMEDIAL 3°:**	Not preserved	**INTER-3° ANGLE TO 1°:**	Acute to midvein
**ADMEDIAL COURSE:**	Not preserved	**EXTERIOR 3° COURSE:**	Not preserved
**EXMEDIAL COURSE:**	not preserved	**INTERCOSTAL 3° VARIABILITY:**	Consistent

FOURTH & HIGHER ORDER VEIN FEATURES

**4° VEIN FABRIC:**	Opposite percurrent	**VEINLETS -F/E/V/s:**	Not Preserved
**5° VEIN FABRIC:**	Regular reticulate	**TYPE OF F.E.V. BRANCHING:**	n.p
**MARGINAL VENATION:**	Not Preserved	**F.E.V.s TERMINATIONS:**	Not Preserved
**AREOLATION:**	Well developed	**LEAF RANK:**	

TOOTH FEATURES

**ORDERS OF TEETH:**	2	**No TEETH/cm:**	4	**TEETH GLANDULARITY:**	Non-specific glandular
**TOOTH SHAPE:**	cv/st	**TOOTH SPACING:**	Irregular		
**PRINCIPAL VEIN:**	Present	**SINUS SHAPE:**	Rounded		
**ACCESSORY VEIN COURSE:**				**PRINCIPAL VEIN TERM.:**	Marginal, at apex

NOTES

This morphosheet was filled out using specimen: 1704_13-A and a previously collected specimen EC1518A

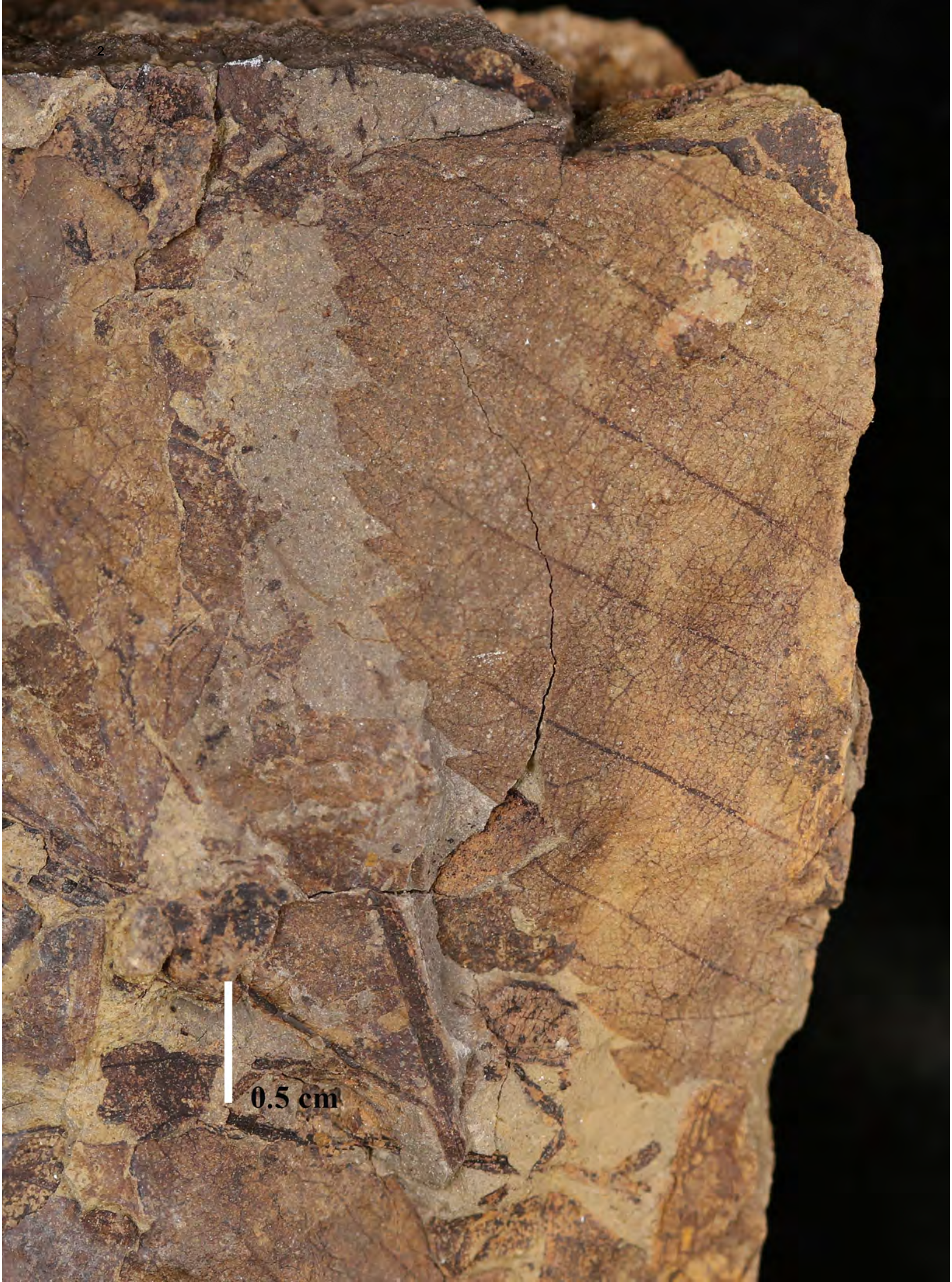
0.5 cm

**MORPHOTYPE**		**NAME**	*Averrhoites affinis*

GENERAL

MAJOR GROUP: DIC
INFERED FAMILY:
ORGAN TYPE:
EXEMPLAR LOC.: HB
EXEMPLAR: 1701-79, 1706-39
OTHERS LOC.:

DIAGNOSTIC FEATURES OF MORPHOTYPE:
Base of midvein thick and decreases in thickness as you move towards the apex.
Midvein at base is 2x thicker than at the apex. The base is also asymetrical intersecondary veins and the preservation is lacking.

MQI:

LEAF ATTACHMENT, ORGANIZATION, SIZE, SHAPE AND PETIOLE FEATURES

LEAF ATTACHMENT:	Petiolate	LEAFLET ORGANIZATION:	Not Preserved
LEAF ARRANGEMENT:	Not Preserved	LEAFLET ATTACHMENT:	np
LEAF ORGANIZATION:	Not Preserved	PETIOLE ATTACHMENT.:	Not Preserved
BLADE SIZE:	Microphyll-Mesophyll	PETIOLE BASE:	Not Preserved
BLADE SHAPE:	Elliptic	PETIOLE GLANDS:.	NP
BLADE RATIO L:W:	2:1	PETIOLE X-SECTION:	NP
LOBATION:	Unlobed	MARGIN TYPE	Untoothed
BASE ASYMMETRY:	Basal width asymmetrical	MEDIAL SYMMETRY:	Asymetrical

BASE SHAPE:	Convex	APEX SHAPE:	Rounded	Special Margin Features:	Not Preserved
BASE ANGLE:	Acute	APEX ANGLE:	Obtuse	Terminal apex features:	Not Preserved

PRIMARY & SECONDARY VEIN FEATURES

		NAKED BASAL VEINS:	Absent
PRIMARY VENATION:	Pinnate	N° BASAL VEINS:	1 (Interior 2°s: Absent)
MAJOR 2° Framework:	Simple brochidodromous	MINOR-2° Course:	Semicraspedodromous
MAJOR 2° Attachment:	Decurrent	MAJOR 2° VEIN ANGLE:	Abruptly increasing toward base
MAJOR 2° SPACING:	Irregular	AGROPHIC VEINS:	Absent
INTER-2° proximal course:		INTER-2° VEIN LENGTH:	
INTER-2° distal course:		INTER-2° FREQUENCY:	

TERTIARY VEIN FEATURES

INTERCOSTAL 3°:	Alternate percurrent	PERIMARGINAL VEINS:	None
EPIMEDIAL 3°:	Not preserved	INTER-3° ANGLE TO 1°:	Not Preserved
ADMEDIAL COURSE:	Not preserved	EXTERIOR 3° COURSE:	Not Preserved
EXMEDIAL COURSE:	Not Preserved	INTERCOSTAL 3° VARIABILITY:	Not Preserved

FOURTH & HIGHER ORDER VEIN FEATURES

4° VEIN FABRIC:	Not Preserved	VEINLETS -F/E/V/s:	Not Preserved
5° VEIN FABRIC:	Not Preserved	TYPE OF F.E.V. BRANCHING:	n.p
MARGINAL VENATION:	Not Preserved	F.E.V.s TERMINATIONS:	Not Preserved
AREOLATION:	Not Preserved	LEAF RANK:	

TOOTH FEATURES

ORDERS OF TEETH:		No TEETH/cm:		TEETH GLANDULARITY:	
TOOTH SHAPE:		TOOTH SPACING:			
PRINCIPAL VEIN:		SINUS SHAPE:			
ACCESSORY VEIN COURSE:		PRINCIPAL VEIN TERM.:			

NOTES

Specimens used to make sheet: 1701-79 and 1706-39

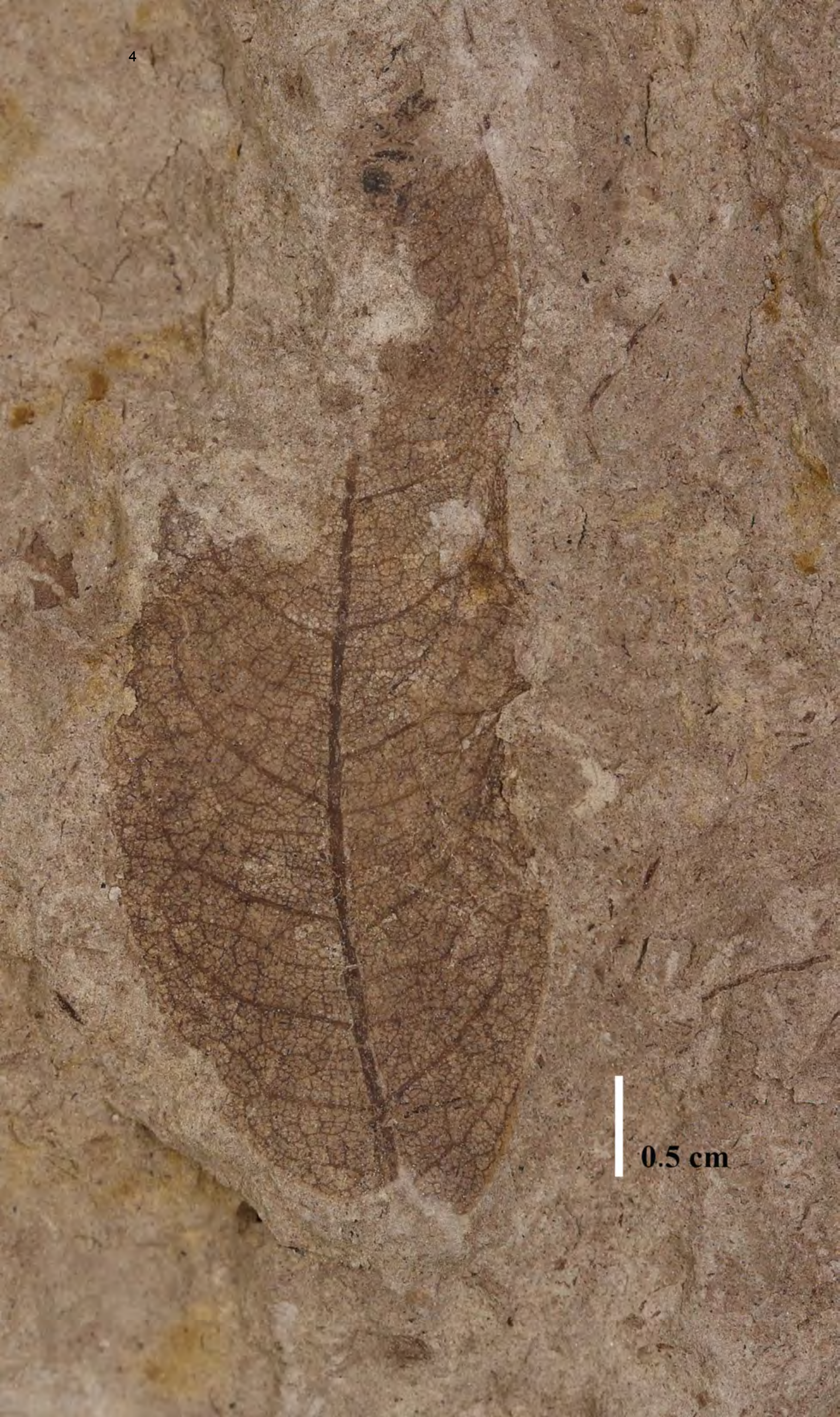
0.5 cm

MORPHOTYPE		NAME	*Fagopsiphyllum groenlandicum*

GENERAL

MAJOR GROUP: DIC — INFERED FAMILY: — ORGAN TYPE:

EXEMPLAR LOC.: Hanna Basin, WY — EXEMPLAR: 1710-20 — OTHERS LOC.:

DIAGNOSTIC FEATURES OF MORPHOTYPE:
Large crenate teeth with thick 2° vein that terminates in the apex of the tooth. Only one order of teeth and 1 tooth/cm. This distinguishes it apart from other crenate toothed leaves from this basin.

MQI:

LEAF ATTACHMENT, ORGANIZATION, SIZE, SHAPE AND PETIOLE FEATURES

LEAF ATTACHMENT:	Not Preserved	LEAFLET ORGANIZATION:	Not Preserved
LEAF ARRANGEMENT:	Not Preserved	LEAFLET ATTACHMENT:	np
LEAF ORGANIZATION:	Not Preserved	PETIOLE ATTACHMENT.:	Not Preserved
BLADE SIZE:	Microphyll-Notophyllm	PETIOLE BASE:	Not Preserved
BLADE SHAPE:	Ovate	PETIOLE GLANDS:.	Not Preserved
BLADE RATIO L:W:	1.5:1	PETIOLE X-SECTION:	Not Preserved
LOBATION:	Unlobed	MARGIN TYPE	Crenate
BASE ASYMMETRY:	Not Preserved	MEDIAL SYMMETRY:	Not Preserved

BASE SHAPE:	Not Preserved	APEX SHAPE:	Not preserved	Special Margin Features:	Not Preserved
BASE ANGLE:	Not Preserved	APEX ANGLE:	Not preserved	Terminal apex features:	Not Preserved

PRIMARY & SECONDARY VEIN FEATURES

		NAKED BASAL VEINS:	Not Preserved
PRIMARY VENATION:	Pinnate	N° BASAL VEINS:	1 — Interior 2°: Absent
MAJOR 2° Framework:	Craspedodromous	MINOR-2° Course:	
MAJOR 2° Attachment:	Decurrent	MAJOR 2° VEIN ANGLE:	Smoothly increasing toward base
MAJOR 2° SPACING:	Regular	AGROPHIC VEINS:	Absent
INTER-2° proximal course:		INTER-2° VEIN LENGTH:	
INTER-2° distal course:		INTER-2° FREQUENCY:	

TERTIARY VEIN FEATURES

INTERCOSTAL 3°:	Mixed opp/alt percurrent	PERIMARGINAL VEINS:	None
EPIMEDIAL 3°:	Not preserved	INTER-3° ANGLE TO 1°:	Acute to midvein
ADMEDIAL COURSE:	Not Preserved	EXTERIOR 3° COURSE:	Not Preserved
EXMEDIAL COURSE:	Not Preserved	INTERCOSTAL 3° VARIABILITY:	Not Preserved

FOURTH & HIGHER ORDER VEIN FEATURES

4° VEIN FABRIC:	Not Preserved	VEINLETS -F/E/V/s:	Not Preserved
5° VEIN FABRIC:	Not Preserved	TYPE OF F.E.V. BRANCHING:	n.p
MARGINAL VENATION:	Not Preserved	F.E.V.s TERMINATIONS:	Not Preserved
AREOLATION:	Not Preserved	LEAF RANK:	

TOOTH FEATURES

ORDERS OF TEETH:	1	No TEETH/cm:	1	TEETH GLANDULARITY:	Not Preserved
TOOTH SHAPE:	rt/rt	TOOTH SPACING:	Regular		
PRINCIPAL VEIN:	Present	SINUS SHAPE:	Rounded		
ACCESSORY VEIN COURSE:	Not Preserved	PRINCIPAL VEIN TERM.:	Marginal, at apex		

NOTES

Morphotype sheet completed with specimens: 1710-20 snd 1703-7

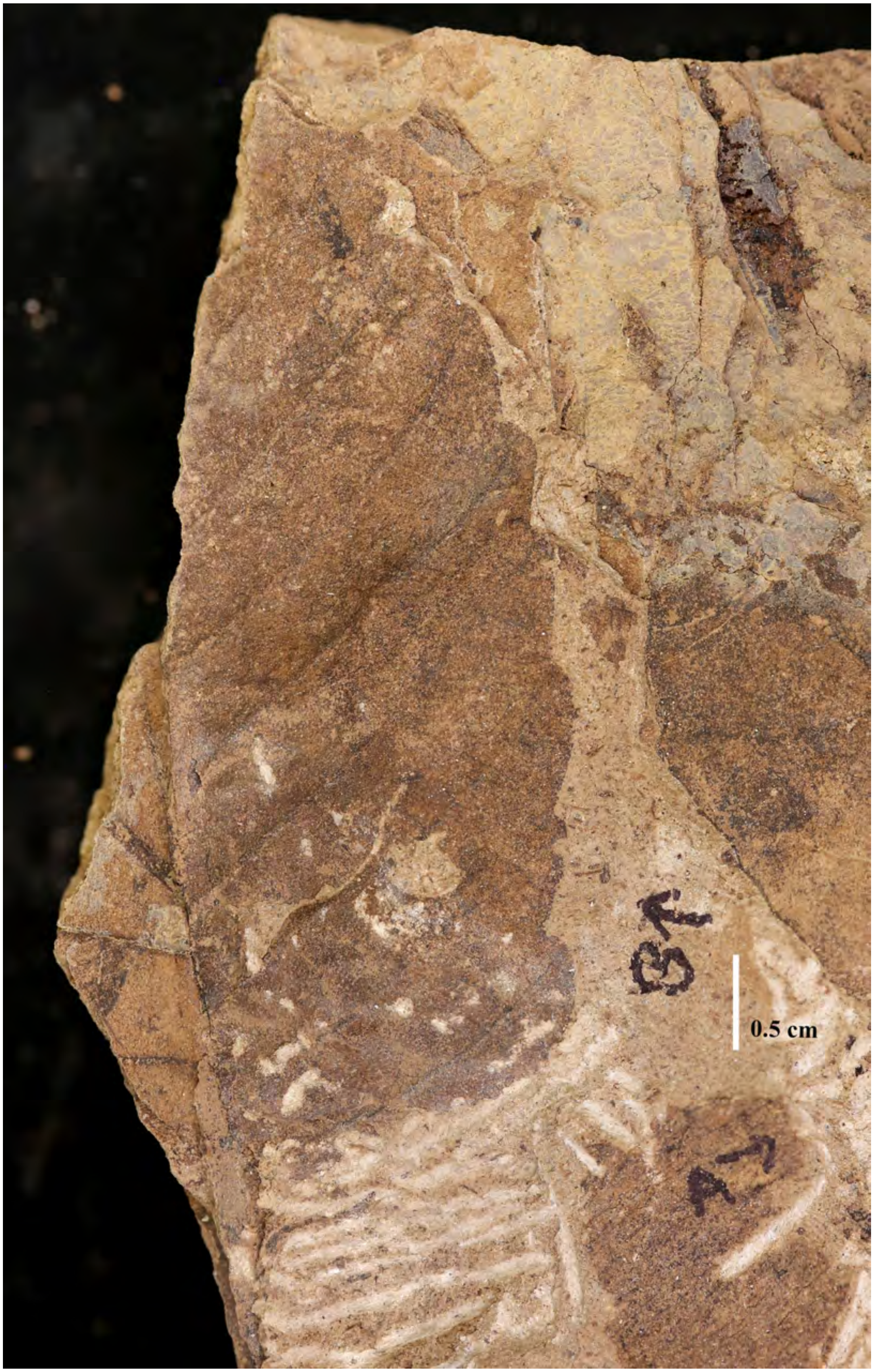
B
A
0.5 cm

MORPHOTYPE	HB171	NAME	

GENERAL

MAJOR GROUP: DIC | INFERED FAMILY: | ORGAN TYPE: Leaf

EXEMPLAR LOC.: Hanna Basin 1706 | EXEMPLAR: 1706-42 | OTHERS LOC.:

DIAGNOSTIC FEATURES OF MORPHOTYPE:

Thickened margin but not enough preserved to claim a perimarginal vein.
Also the base is wide obtuse and concave, and the intercostal 3 are chevroned.

MQI:

LEAF ATTACHMENT, ORGANIZATION, SIZE, SHAPE AND PETIOLE FEATURES

LEAF ATTACHMENT:	Not Preserved	LEAFLET ORGANIZATION:	Not Preserved
LEAF ARRANGEMENT:	Not Preserved	LEAFLET ATTACHMENT:	np
LEAF ORGANIZATION:	Not Preserved	PETIOLE ATTACHMENT.:	Not Preserved
BLADE SIZE:	Notophyll	PETIOLE BASE:	Not Preserved
BLADE SHAPE:	Ovate	PETIOLE GLANDS:.	Not Preserved
BLADE RATIO L:W:	1:1	PETIOLE X-SECTION:	Not Preserved
LOBATION:	Not Preserved	MARGIN TYPE	Untoothed
BASE ASYMMETRY:	Symmetrical	MEDIAL SYMMETRY:	Symmetrical

BASE SHAPE:	Concave	APEX SHAPE:	Not preserved	Special Margin Features:	Not Preserved
BASE ANGLE:	Wide obtuse	APEX ANGLE:	Not preserved	Terminal apex features:	Not Preserved

PRIMARY & SECONDARY VEIN FEATURES

		NAKED BASAL VEINS:	Absent
PRIMARY VENATION:	Pinnate	N° BASAL VEINS:	1 — Interior 2°: Absent
MAJOR 2° Framework:	Simple brochidodromous	MINOR-2° Course:	Not Preserved
MAJOR 2° Attachment:	Decurrent	MAJOR 2° VEIN ANGLE:	Smoothly increasing toward base
MAJOR 2° SPACING:	Irregular	AGROPHIC VEINS:	Absent
INTER-2° proximal course:		INTER-2° VEIN LENGTH:	
INTER-2° distal course:		INTER-2° FREQUENCY:	

TERTIARY VEIN FEATURES

INTERCOSTAL 3°:	Opposite percurrent - chevroned	PERIMARGINAL VEINS:	Not Preserved
EPIMEDIAL 3°:	Not preserved	INTER-3° ANGLE TO 1°:	Not Preserved
ADMEDIAL COURSE:	Not Preserved	EXTERIOR 3° COURSE:	Not Preserved
EXMEDIAL COURSE:	Not Preserved	INTERCOSTAL 3° VARIABILITY:	Not Preserved

FOURTH & HIGHER ORDER VEIN FEATURES

4° VEIN FABRIC:	Not Preserved	VEINLETS -F/E/V/s:	Not Preserved
5° VEIN FABRIC:	Not Preserved	TYPE OF F.E.V. BRANCHING:	n.p
MARGINAL VENATION:	Not Preserved	F.E.V.s TERMINATIONS:	Not Preserved
AREOLATION:	Not Preserved	LEAF RANK:	

TOOTH FEATURES

ORDERS OF TEETH:		No TEETH/cm:		TEETH GLANDULARITY:	
TOOTH SHAPE:		TOOTH SPACING:			
PRINCIPAL VEIN:		SINUS SHAPE:			
ACCESSORY VEIN COURSE:		PRINCIPAL VEIN TERM.:			

NOTES

Specimen 1706_42 was used to make this morphosheet

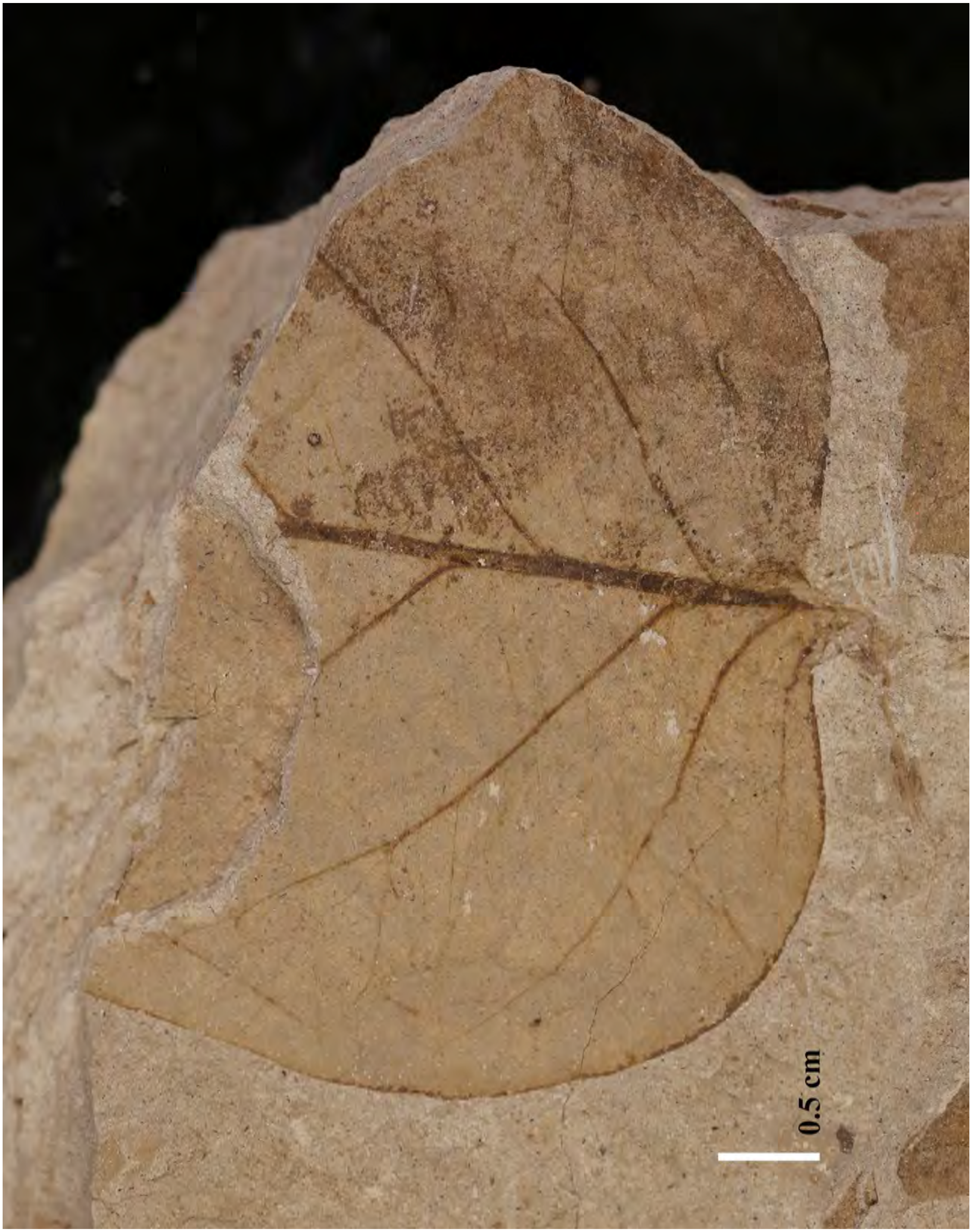
0.5 cm

MORPHOTYPE	HB172	NAME	

GENERAL

MAJOR GROUP DIC **INFERED FAMILY** **ORGAN TYPE** Leaf

EXEMPLAR LOC. Hanna Basin WY **EXEMPLAR** 1701-1 **OTHERS LOC.**

DIAGNOSTIC FEATURES OF MORPHOTYPE Cordate base.

Thickened midvein at base and decreases towards apex. Base is 3x thicker than apex.

Higher order veins are not preserved. Second degree veins are very distinct with a sharp increase toward the base of the leaf.

MQI:

LEAF ATTACHMENT, ORGANIZATION, SIZE, SHAPE AND PETIOLE FEATURES

LEAF ATTACHMENT:	Petiolate	LEAFLET ORGANIZATION:	Not Preserved
LEAF ARRANGEMENT:	Not Preserved	LEAFLET ATTACHMENT:	np
LEAF ORGANIZATION:	Not Preserved	PETIOLE ATTACHMENT.:	Not Preserved
BLADE SIZE:	Nanophyll-Notophyll	PETIOLE BASE:	Not Preserved
BLADE SHAPE:	Oblong	PETIOLE GLANDS:.	NP
BLADE RATIO L:W:	1.5:1	PETIOLE X-SECTION:	NP
LOBATION:	Unlobed	MARGIN TYPE	Serrate
BASE ASYMMETRY:	Symmetrical	MEDIAL SYMMETRY:	Symmetrical

BASE SHAPE:	Cordate	APEX SHAPE:	Not preserved	Special Margin Features:	Not Preserved
BASE ANGLE:	Wide obtuse	APEX ANGLE:	Not preserved	Terminal apex features:	Not Preserved

PRIMARY & SECONDARY VEIN FEATURES

		NAKED BASAL VEINS:	Absent
PRIMARY VENATION:	Pinnate	N° BASAL VEINS:	1 — Interior 2°: Absent
MAJOR 2° Framework:	Semicraspedodromous	MINOR-2° Course:	
MAJOR 2° Attachment:	Decurrent	MAJOR 2° VEIN ANGLE:	Abruptly increasing toward base
MAJOR 2° SPACING:	Regular	AGROPHIC VEINS:	Absent
INTER-2° proximal course:		INTER-2° VEIN LENGTH:	
INTER-2° distal course:		INTER-2° FREQUENCY:	

TERTIARY VEIN FEATURES

INTERCOSTAL 3°:	Opposite percurrent - straight	PERIMARGINAL VEINS:	None
EPIMEDIAL 3°:	Opposite percurrent	INTER-3° ANGLE TO 1°:	Acute to midvein
ADMEDIAL COURSE:	Acute to midvein	EXTERIOR 3° COURSE:	Absent
EXMEDIAL COURSE:	Basiflexed	INTERCOSTAL 3° VARIABILITY:	Increasing exmedially

FOURTH & HIGHER ORDER VEIN FEATURES

4° VEIN FABRIC:	Not Preserved	VEINLETS -F/E/V/s:	Not Preserved
5° VEIN FABRIC:	Not Preserved	TYPE OF F.E.V. BRANCHING:	n.p
MARGINAL VENATION:	Not Preserved	F.E.V.s TERMINATIONS:	Not Preserved
AREOLATION:	Not Preserved	LEAF RANK:	

TOOTH FEATURES

ORDERS OF TEETH:	1	No TEETH/cm:	1	TEETH GLANDULARITY:	Not Preserved
TOOTH SHAPE:	cv/cc	TOOTH SPACING:	Not Preserved		
PRINCIPAL VEIN:	Present	SINUS SHAPE:	Angular		
ACCESSORY VEIN COURSE:	Not Preserved	PRINCIPAL VEIN TERM.:	Marginal, on proximal flank		

NOTES

Morphotype sheet filled out using specimens: 1706-4 and 1701-1

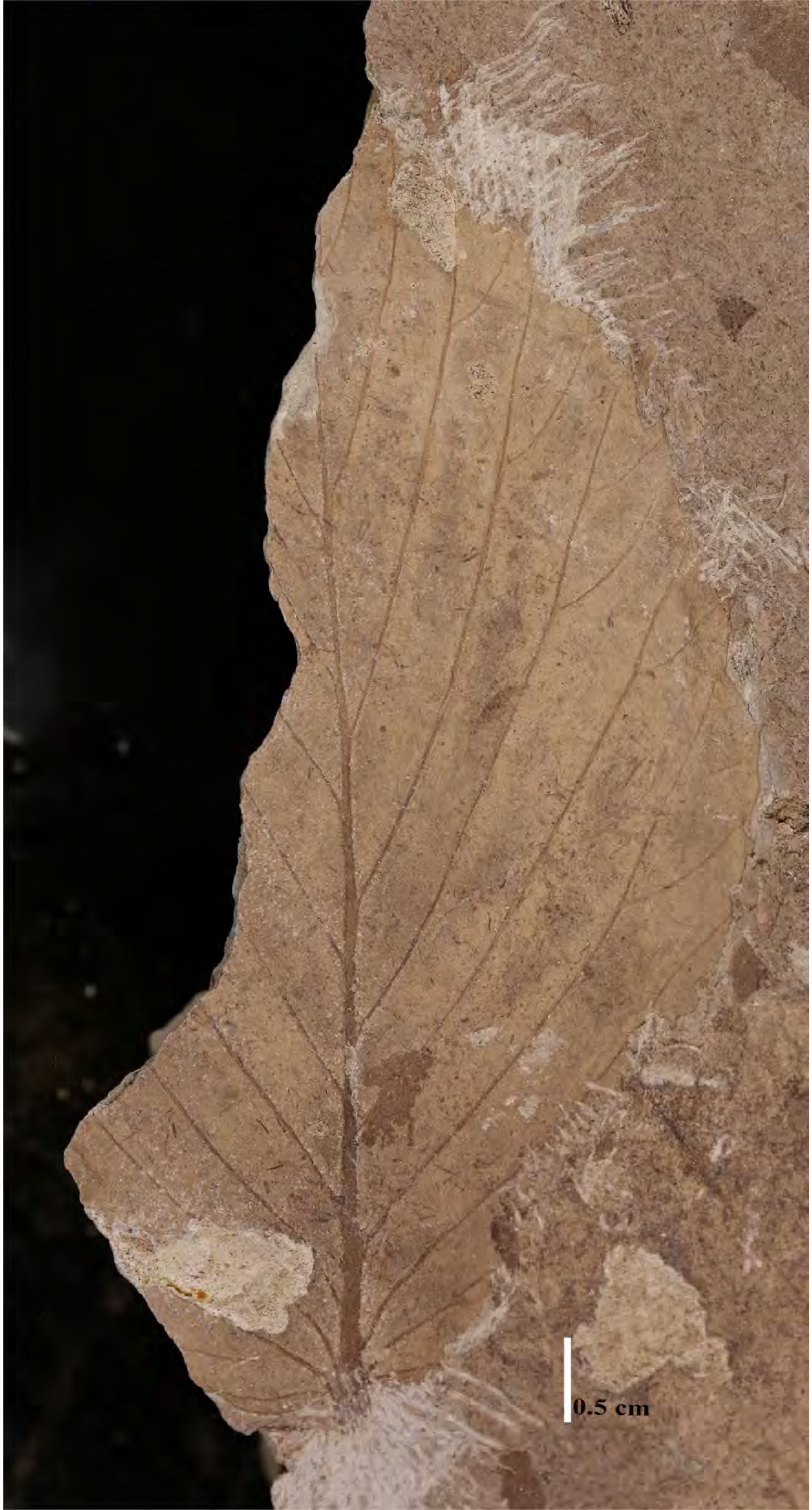
0.5 cm

MORPHOTYPE	HB173	NAME	

GENERAL

MAJOR GROUP: DIC **INFERED FAMILY:** **ORGAN TYPE:** Leaf

EXEMPLAR LOC.: Hanna Basin, WY **EXEMPLAR:** 1712-1 **OTHERS LOC.:**

DIAGNOSTIC FEATURES OF MORPHOTYPE: 3° veins perpendicular to midvein at the base but becomes acute to midvein towards the apex. Beautifully looped agrophic veins and very consistant, closely spaced 3° veins at the base. Lastly this morphotype has an asymmetrical basal insertion.

MQI:

LEAF ATTACHMENT, ORGANIZATION, SIZE, SHAPE AND PETIOLE FEATURES

LEAF ATTACHMENT:	Petiolate	LEAFLET ORGANIZATION:	Not Preserved
LEAF ARRANGEMENT:	Not Preserved	LEAFLET ATTACHMENT:	np
LEAF ORGANIZATION:	Not Preserved	PETIOLE ATTACHMENT.:	Not Preserved
BLADE SIZE:	Microphyll-Mesophyll	PETIOLE BASE:	Not Preserved
BLADE SHAPE:	Elliptic	PETIOLE GLANDS:.	Not Preserved
BLADE RATIO L:W:	1.5:1	PETIOLE X-SECTION:	Not Preserved
LOBATION:	Unlobed	MARGIN TYPE	Untoothed
BASE ASYMMETRY:	Basal insertion asymmetrical	MEDIAL SYMMETRY:	Symmetrical

BASE SHAPE:	Rounded	APEX SHAPE:	Not preserved	Special Margin Features:	Not Preserved
BASE ANGLE:	Obtuse	APEX ANGLE:	Not preserved	Terminal apex features:	Not Preserved

PRIMARY & SECONDARY VEIN FEATURES

NAKED BASAL VEINS: Absent

PRIMARY VENATION:	Basal actinodromous	N° BASAL VEINS:	3 (Interior 2°: Absent)
MAJOR 2° Framework:	Eucamptodromous	MINOR-2° Course:	
MAJOR 2° Attachment:	Decurrent	MAJOR 2° VEIN ANGLE:	Smoothly increasing toward base
MAJOR 2° SPACING:	Decreasing proximally	AGROPHIC VEINS:	Simple
INTER-2° proximal course:		INTER-2° VEIN LENGTH:	
INTER-2° distal course:		INTER-2° FREQUENCY:	

TERTIARY VEIN FEATURES

INTERCOSTAL 3°:	Mixed opp/alt percurrent	PERIMARGINAL VEINS:	None
EPIMEDIAL 3°:	Opposite percurrent	INTER-3° ANGLE TO 1°:	Perpendicular to midvein
ADMEDIAL COURSE:	Perpendicular to midvein	EXTERIOR 3° COURSE:	Looped
EXMEDIAL COURSE:	Basiflexed	INTERCOSTAL 3° VARIABILITY:	Inconsistent

FOURTH & HIGHER ORDER VEIN FEATURES

4° VEIN FABRIC:	Not Preserved	VEINLETS -F/E/V/s:	Not Preserved
5° VEIN FABRIC:	Not Preserved	TYPE OF F.E.V. BRANCHING:	n.p
MARGINAL VENATION:	Not Preserved	F.E.V.s TERMINATIONS:	Not Preserved
AREOLATION:	Not Preserved	LEAF RANK:	

TOOTH FEATURES

ORDERS OF TEETH:		No TEETH/cm:		TEETH GLANDULARITY:	
TOOTH SHAPE:		TOOTH SPACING:			
PRINCIPAL VEIN:		SINUS SHAPE:			
ACCESSORY VEIN COURSE:		PRINCIPAL VEIN TERM.:			

NOTES

Morphotype sheet completed with specimens: 17012-1, 1704-80 and 1710-214

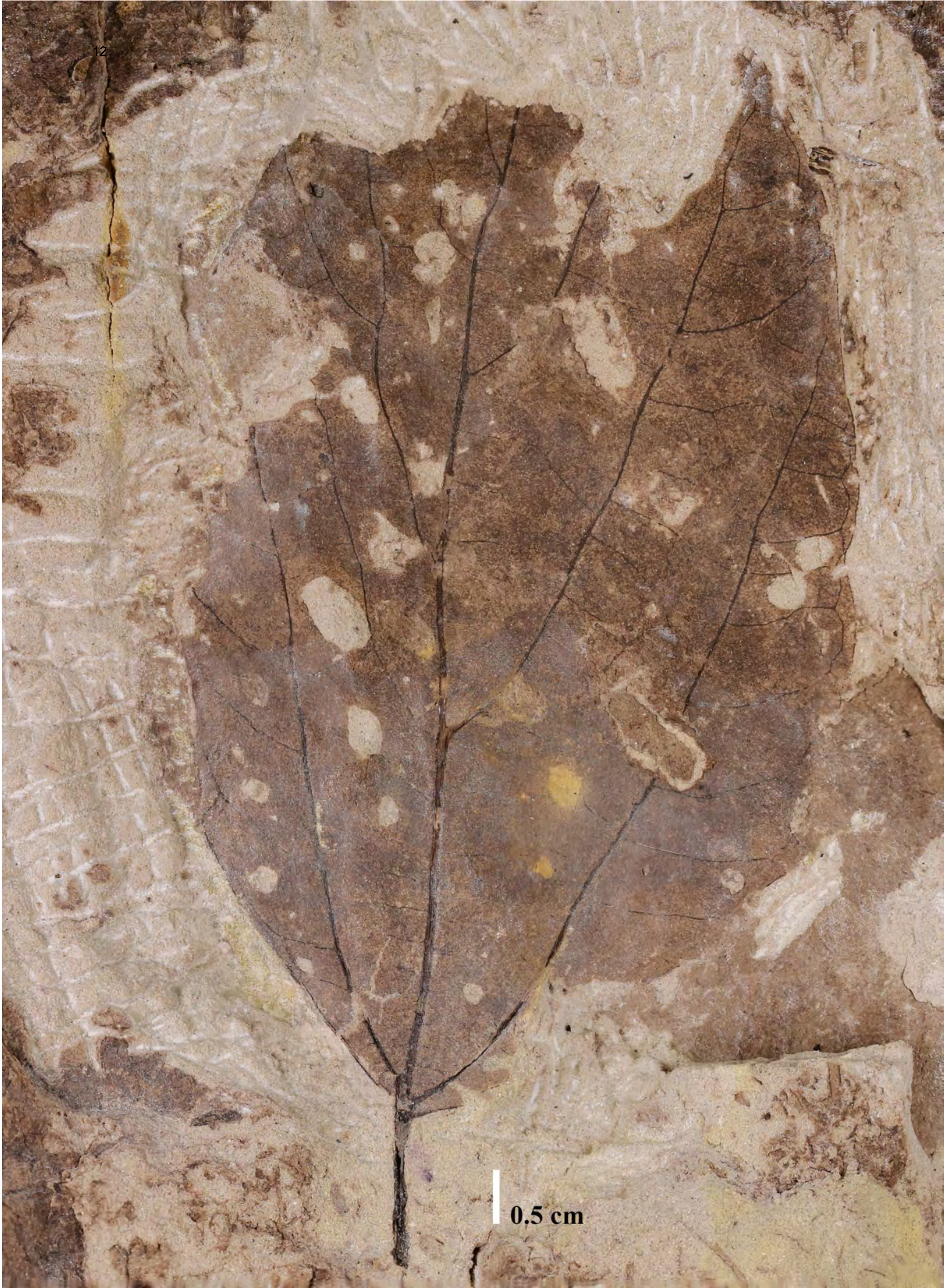
0.5 cm

MORPHOTYPE	HB174	NAME	

GENERAL

MAJOR GROUP	DIC	INFERED FAMILY		ORGAN TYPE	Leaf
EXEMPLAR LOC.	Hanna Basin, WY	EXEMPLAR	1710-6A, 1710-13B	OTHERS LOC.	

DIAGNOSTIC FEATURES OF MORPHOTYPE: Crenate teeth with only one order of teeth and 5 teeth/cm. This is different from Fagopsophyllum which only has 1 tooth/cm. 2° veins spacing is regular howe\ the veins are more compressed at the base and apex. This is a morphological feature that is unlike any other morphotype within the basin. The base is also cordate.

MQI:

LEAF ATTACHMENT, ORGANIZATION, SIZE, SHAPE AND PETIOLE FEATURES

LEAF ATTACHMENT:	Not Preserved	LEAFLET ORGANIZATION:	Not Preserved
LEAF ARRANGEMENT:	Not Preserved	LEAFLET ATTACHMENT:	np
LEAF ORGANIZATION:	Not Preserved	PETIOLE ATTACHMENT.:	Not Preserved
BLADE SIZE:	Nanophyll-Mesophyll	PETIOLE BASE:	Not Preserved
BLADE SHAPE:	Ovate	PETIOLE GLANDS:.	Not Preserved
BLADE RATIO L:W:	2:1	PETIOLE X-SECTION:	Not Preserved
LOBATION:	Unlobed	MARGIN TYPE	Crenate
BASE ASYMMETRY:	Symmetrical	MEDIAL SYMMETRY:	Symmetrical

BASE SHAPE:	Cordate	APEX SHAPE:	Not preserved	Special Margin Features:	Not Preserved
BASE ANGLE:	Obtuse	APEX ANGLE:	Not preserved	Terminal apex features:	Not Preserved

PRIMARY & SECONDARY VEIN FEATURES

		NAKED BASAL VEINS:	Absent
PRIMARY VENATION:	Pinnate	N° BASAL VEINS:	1 — Interior 2°: Absent
MAJOR 2° Framework:	Eucamptodromous	MINOR-2° Course:	
MAJOR 2° Attachment:	Decurrent	MAJOR 2° VEIN ANGLE:	Smoothly increasing toward base
MAJOR 2° SPACING:	Regular	AGROPHIC VEINS:	Absent
INTER-2° proximal course:		INTER-2° VEIN LENGTH:	
INTER-2° distal course:		INTER-2° FREQUENCY:	

TERTIARY VEIN FEATURES

INTERCOSTAL 3°:	Opposite percurrent - straight	PERIMARGINAL VEINS:	None
EPIMEDIAL 3°:	Opposite percurrent	INTER-3° ANGLE TO 1°:	Acute to midvein
ADMEDIAL COURSE:	Perpendicular to midvein	EXTERIOR 3° COURSE:	Looped
EXMEDIAL COURSE:	Basiflexed	INTERCOSTAL 3° VARIABILITY:	Consistent

FOURTH & HIGHER ORDER VEIN FEATURES

4° VEIN FABRIC:	Not Preserved	VEINLETS -F/E/V/s:	Not Preserved
5° VEIN FABRIC:	Not Preserved	TYPE OF F.E.V. BRANCHING:	n.p
MARGINAL VENATION:	Not Preserved	F.E.V.s TERMINATIONS:	Not Preserved
AREOLATION:	Not Preserved	LEAF RANK:	

TOOTH FEATURES

ORDERS OF TEETH:	1	No TEETH/cm:	5	TEETH GLANDULARITY:	Not Preserved
TOOTH SHAPE:	cv/rt	TOOTH SPACING:	Regular		
PRINCIPAL VEIN:	Absent	SINUS SHAPE:	Rounded		
ACCESSORY VEIN COURSE:	Looped	PRINCIPAL VEIN TERM.:	Not Preserved		

NOTES

Morphosheet completed with spimens: 1710-6A and 1710-13B

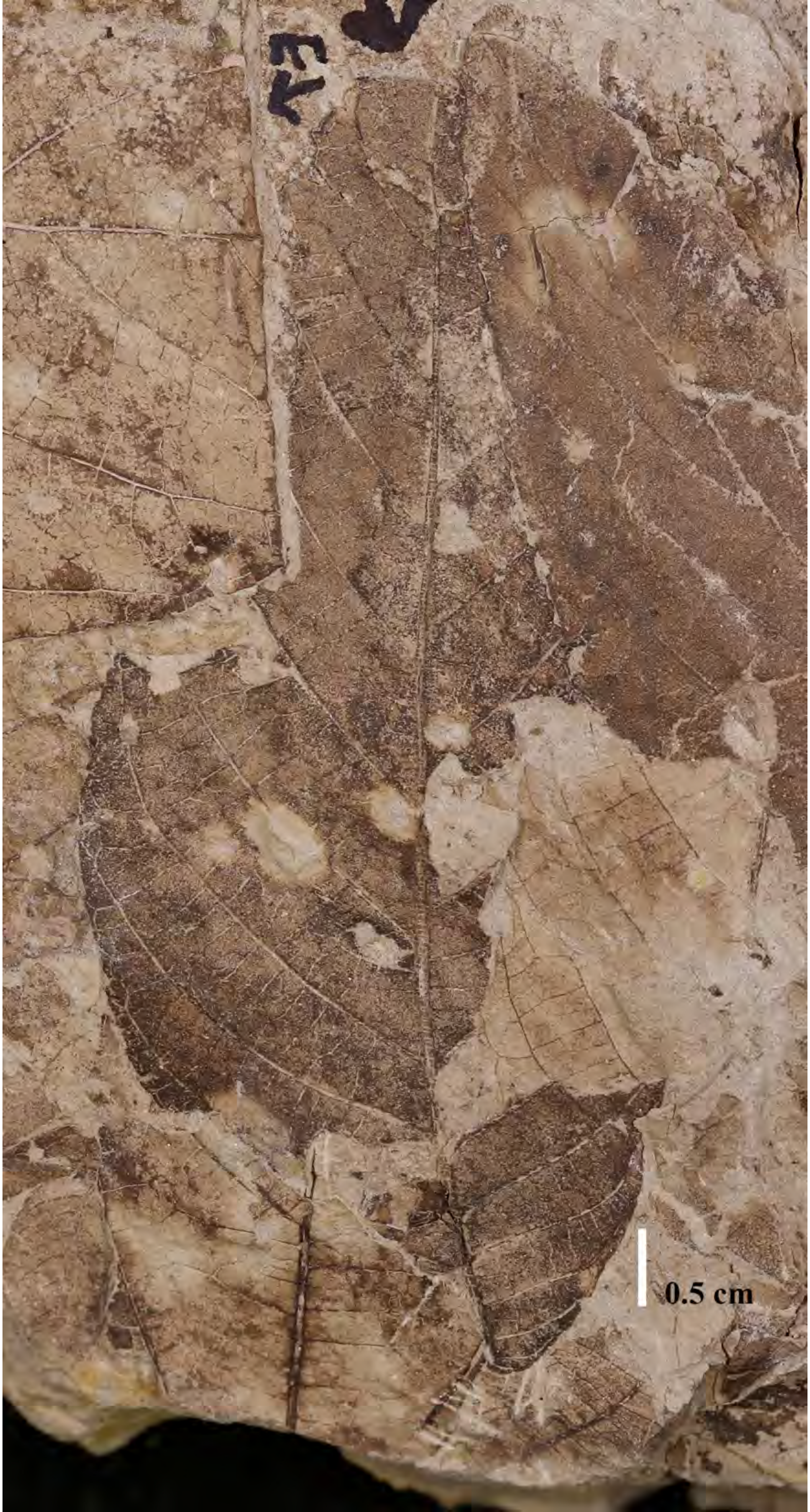
0.5 cm

MORPHOTYPE	HB175	NAME	

GENERAL

MAJOR GROUP: DIC
INFERED FAMILY:
ORGAN TYPE: Leaf
EXEMPLAR LOC.: LAS1703
EXEMPLAR: 1703-2, 1704-111A
OTHERS LOC.: LAS 1704
DIAGNOSTIC FEATURES OF MORPHOTYPE: 2° veins are regularly spaced throughout the leaf, while the higher order veins at the apex and base are rarely preserved. Unlike Fagopsophyllum, this species has serrate teeth with 2 orders and averages 4 teeth/cm.
MQI:

LEAF ATTACHMENT, ORGANIZATION, SIZE, SHAPE AND PETIOLE FEATURES

LEAF ATTACHMENT: Petiolate
LEAFLET ORGANIZATION: Not Preserved
LEAF ARRANGEMENT: Not Preserved
LEAFLET ATTACHMENT: np
LEAF ORGANIZATION: Not Preserved
PETIOLE ATTACHMENT.: Not Preserved
BLADE SIZE: Microphyll-Notophyll
PETIOLE BASE: Not Preserved
BLADE SHAPE: Not Preserved
PETIOLE GLANDS:. NP
BLADE RATIO L:W: 2:1
PETIOLE X-SECTION: NP
LOBATION: Unlobed
MARGIN TYPE: Serrate
BASE ASYMMETRY: Not Preserved
MEDIAL SYMMETRY: Not Preserved
BASE SHAPE: Rounded
APEX SHAPE: Acuminate
Special Margin Features: Not Preserved
BASE ANGLE: Obtuse
APEX ANGLE: Acute
Terminal apex features: Not Preserved

PRIMARY & SECONDARY VEIN FEATURES

NAKED BASAL VEINS: Not Preserved
PRIMARY VENATION: Pinnate
N° BASAL VEINS: 1
Interior 2°: Absent
MAJOR 2° Framework: Craspedodromous
MINOR-2° Course:
MAJOR 2° Attachment: Excurrent
MAJOR 2° VEIN ANGLE: Uniform
MAJOR 2° SPACING: Regular
AGROPHIC VEINS: Absent
INTER-2° proximal course:
INTER-2° VEIN LENGTH:
INTER-2° distal course:
INTER-2° FREQUENCY:

TERTIARY VEIN FEATURES

INTERCOSTAL 3°: Opposite percurrent - convex
PERIMARGINAL VEINS: None
EPIMEDIAL 3°: Not preserved
INTER-3° ANGLE TO 1°: Acute to midvein
ADMEDIAL COURSE: Obtuse to midvein
EXTERIOR 3° COURSE: Absent
EXMEDIAL COURSE: Basiflexed
INTERCOSTAL 3° VARIABILITY: Increasing proximally

FOURTH & HIGHER ORDER VEIN FEATURES

4° VEIN FABRIC: Opposite percurrent
VEINLETS -F/E/V/s: Not Preserved
5° VEIN FABRIC: Regular reticulate
TYPE OF F.E.V. BRANCHING: n.p
MARGINAL VENATION: Not Preserved
F.E.V.s TERMINATIONS: Not Preserved
AREOLATION: Moderately developed
LEAF RANK:

TOOTH FEATURES

ORDERS OF TEETH: 2
No TEETH/cm: 4
TEETH GLANDULARITY: None
TOOTH SHAPE: cv/rt
TOOTH SPACING: Regular
PRINCIPAL VEIN: Present
SINUS SHAPE: Angular
ACCESSORY VEIN COURSE: Absent
PRINCIPAL VEIN TERM.: Marginal, at apex

NOTES

Specimens used to complete morphosheet: 1703-2 and 1704-111A

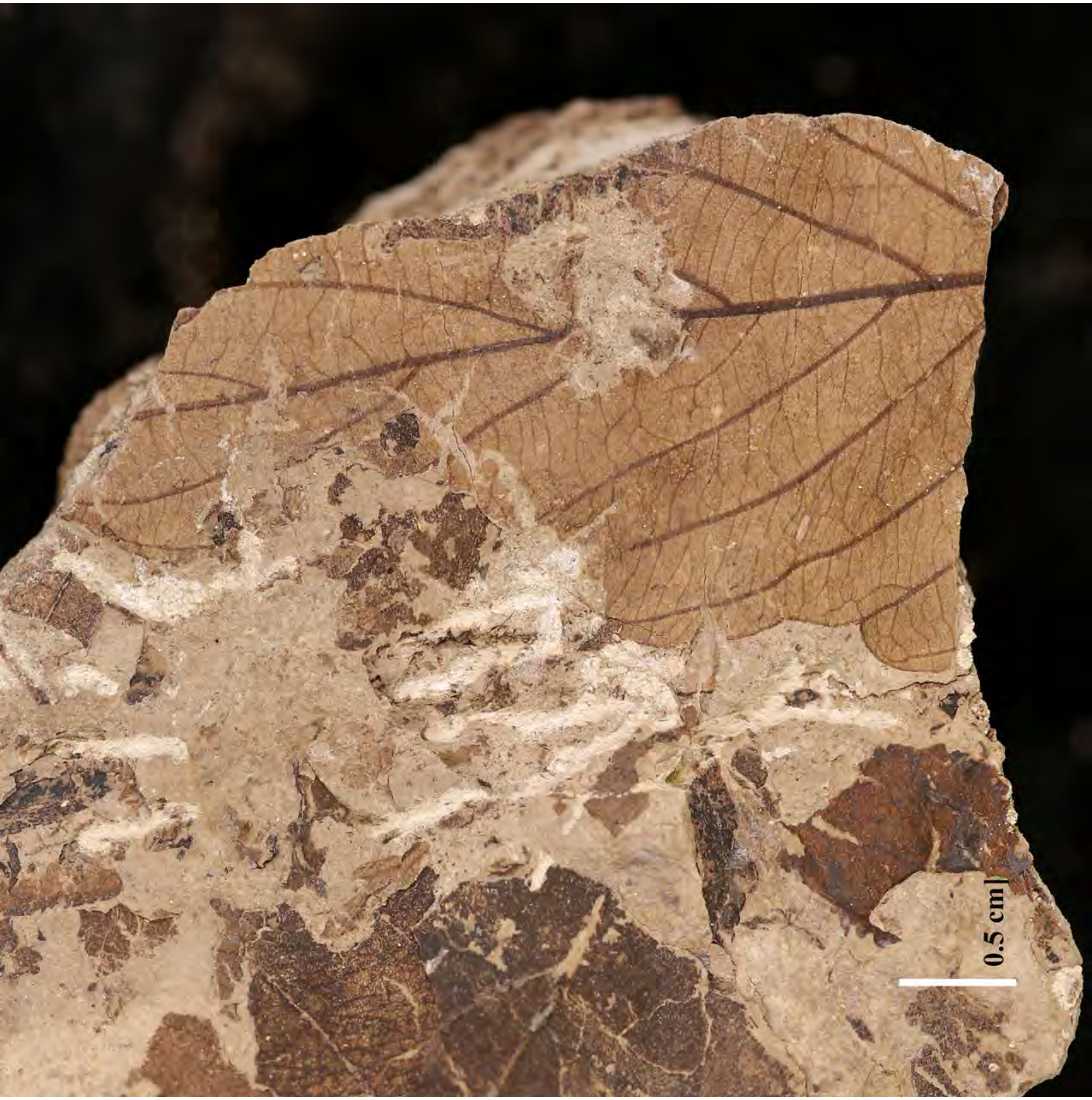
0.5 cm

MORPHOTYPE	HB176	NAME	

GENERAL

MAJOR GROUP	DIC	INFERED FAMILY		ORGAN TYPE	Leaf
EXEMPLAR LOC.	Hanna Basin, WY	EXEMPLAR	1704-1, 1704-76	OTHERS LOC.	

DIAGNOSTIC FEATURES OF MORPHOTYPE: 2° veination is mixed! The framework is both eucamptodromous and simply brochidodromous as you move from the base of the leaf to the apex. When preserved, apex has a very dramatic/extended drip tip.

MQI:

LEAF ATTACHMENT, ORGANIZATION, SIZE, SHAPE AND PETIOLE FEATURES

LEAF ATTACHMENT:	Not Preserved	LEAFLET ORGANIZATION:	Not Preserved
LEAF ARRANGEMENT:	Not Preserved	LEAFLET ATTACHMENT:	np
LEAF ORGANIZATION:	Not Preserved	PETIOLE ATTACHMENT.:	Not Preserved
BLADE SIZE:	Microphyll-Mesohyll	PETIOLE BASE:	Not Preserved
BLADE SHAPE:	Not Preserved	PETIOLE GLANDS:.	NP
BLADE RATIO L:W:	1.5:1	PETIOLE X-SECTION:	NP
LOBATION:	Unlobed	MARGIN TYPE	Untoothed
BASE ASYMMETRY:	Not Preserved	MEDIAL SYMMETRY:	Symmetrical

BASE SHAPE:	Convex	APEX SHAPE:	Acuminate	Special Margin Features:	Not Preserved
BASE ANGLE:	Obtuse	APEX ANGLE:	Acute	Terminal apex features:	

PRIMARY & SECONDARY VEIN FEATURES

		NAKED BASAL VEINS:	Not Preserved
PRIMARY VENATION:	Pinnate	N° BASAL VEINS:	1
		Interior 2°:	Absent
MAJOR 2° Framework:	Mixed framework	MINOR-2° Course:	
MAJOR 2° Attachment:	Decurrent	MAJOR 2° VEIN ANGLE:	Smoothly increasing toward base
MAJOR 2° SPACING:	Regular	AGROPHIC VEINS:	Absent
INTER-2° proximal course:		INTER-2° VEIN LENGTH:	
INTER-2° distal course:		INTER-2° FREQUENCY:	

TERTIARY VEIN FEATURES

INTERCOSTAL 3°:	Mixed opp/alt percurrent	PERIMARGINAL VEINS:	None
EPIMEDIAL 3°:	Mixed opp/alt percurrent	INTER-3° ANGLE TO 1°:	Obtuse or perpendicular
ADMEDIAL COURSE:	Acute to midvein	EXTERIOR 3° COURSE:	Looped
EXMEDIAL COURSE:	Basiflexed	INTERCOSTAL 3° VARIABILITY:	Decreasing exmedially

FOURTH & HIGHER ORDER VEIN FEATURES

4° VEIN FABRIC:	Alternate percurrent	VEINLETS -F/E/V/s:	Not Preserved
5° VEIN FABRIC:	Regular reticulate	TYPE OF F.E.V. BRANCHING:	n.p
MARGINAL VENATION:	Not Preserved	F.E.V.s TERMINATIONS:	Not Preserved
AREOLATION:	Well developed	LEAF RANK:	

TOOTH FEATURES

ORDERS OF TEETH:		No TEETH/cm:		TEETH GLANDULARITY:	
TOOTH SHAPE:		TOOTH SPACING:			
PRINCIPAL VEIN:		SINUS SHAPE:			
ACCESSORY VEIN COURSE:		PRINCIPAL VEIN TERM.:			

NOTES

Specimens used for morphosheet: 1704-1, 1704-76

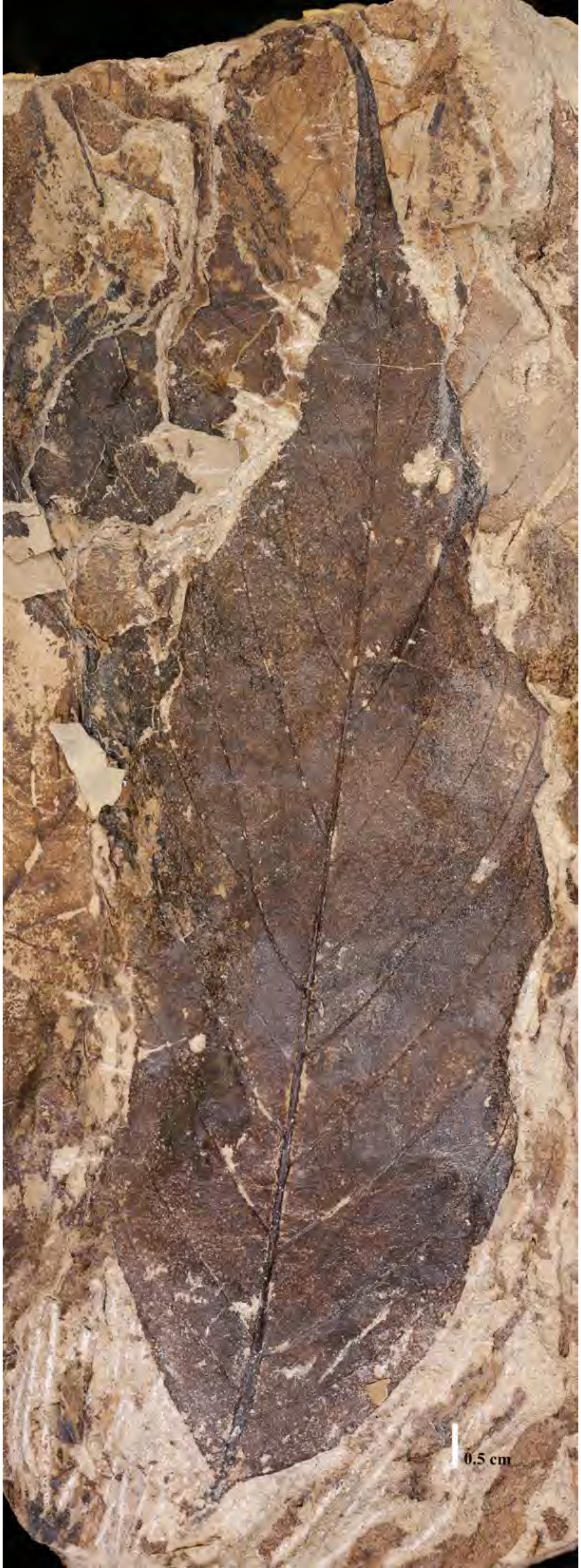
0.5 cm

MORPHOTYPE	HB177	NAME	

GENERAL

MAJOR GROUP	DIC	INFERED FAMILY		ORGAN TYPE	Leaf
EXEMPLAR LOC.	Hanna Basin, WY	EXEMPLAR	1704-9A	OTHERS LOC.	

DIAGNOSTIC FEATURES OF MORPHOTYPE: 2° veins have a mixed framework which is unlike any other pinnate leaf found at these sites. The framework is both brochidodromous and eucamptodromous.

MQI:

LEAF ATTACHMENT, ORGANIZATION, SIZE, SHAPE AND PETIOLE FEATURES

LEAF ATTACHMENT:	Petiolate	LEAFLET ORGANIZATION:	Not Preserved
LEAF ARRANGEMENT:	Not Preserved	LEAFLET ATTACHMENT:	np
LEAF ORGANIZATION:	Not Preserved	PETIOLE ATTACHMENT.:	Not Preserved
BLADE SIZE:	Notophyll	PETIOLE BASE:	Not Preserved
BLADE SHAPE:	Obovate	PETIOLE GLANDS:.	NP
BLADE RATIO L:W:	2:1	PETIOLE X-SECTION:	NP
LOBATION:	Unlobed	MARGIN TYPE	Untoothed
BASE ASYMMETRY:	Symmetrical	MEDIAL SYMMETRY:	Symmetrical

BASE SHAPE:	Convex	APEX SHAPE:	Straight	Special Margin Features:	Not Preserved
BASE ANGLE:	Acute	APEX ANGLE:	Acute	Terminal apex features:	Not Preserved

PRIMARY & SECONDARY VEIN FEATURES

		NAKED BASAL VEINS:	Not Preserved
PRIMARY VENATION:	Pinnate	N° BASAL VEINS:	1 — Interior 2°: Absent
MAJOR 2° Framework:	Mixed framework	MINOR-2° Course:	
MAJOR 2° Attachment:	Decurrent	MAJOR 2° VEIN ANGLE:	Abruptly increasing toward base
MAJOR 2° SPACING:	Regular	AGROPHIC VEINS:	Absent
INTER-2° proximal course:		INTER-2° VEIN LENGTH:	
INTER-2° distal course:		INTER-2° FREQUENCY:	

TERTIARY VEIN FEATURES

INTERCOSTAL 3°:	Opposite percurrent - straight	PERIMARGINAL VEINS:	None
EPIMEDIAL 3°:	Opposite percurrent	INTER-3° ANGLE TO 1°:	Obtuse to midvein
ADMEDIAL COURSE:	Acute to midvein	EXTERIOR 3° COURSE:	Absent
EXMEDIAL COURSE:	Basiflexed	INTERCOSTAL 3° VARIABILITY:	Consistent

FOURTH & HIGHER ORDER VEIN FEATURES

4° VEIN FABRIC:	Not Preserved	VEINLETS -F/E/V/s:	Not Preserved
5° VEIN FABRIC:	Not Preserved	TYPE OF F.E.V. BRANCHING:	n.p
MARGINAL VENATION:	Not Preserved	F.E.V.s TERMINATIONS:	Not Preserved
AREOLATION:	Not Preserved	LEAF RANK:	

TOOTH FEATURES

ORDERS OF TEETH:		No TEETH/cm:		TEETH GLANDULARITY:	
TOOTH SHAPE:		TOOTH SPACING:			
PRINCIPAL VEIN:		SINUS SHAPE:			
ACCESSORY VEIN COURSE:		PRINCIPAL VEIN TERM.:			

NOTES

Morphosheet completed using specimens: 1704-46, 1704-212 and 1704-9A

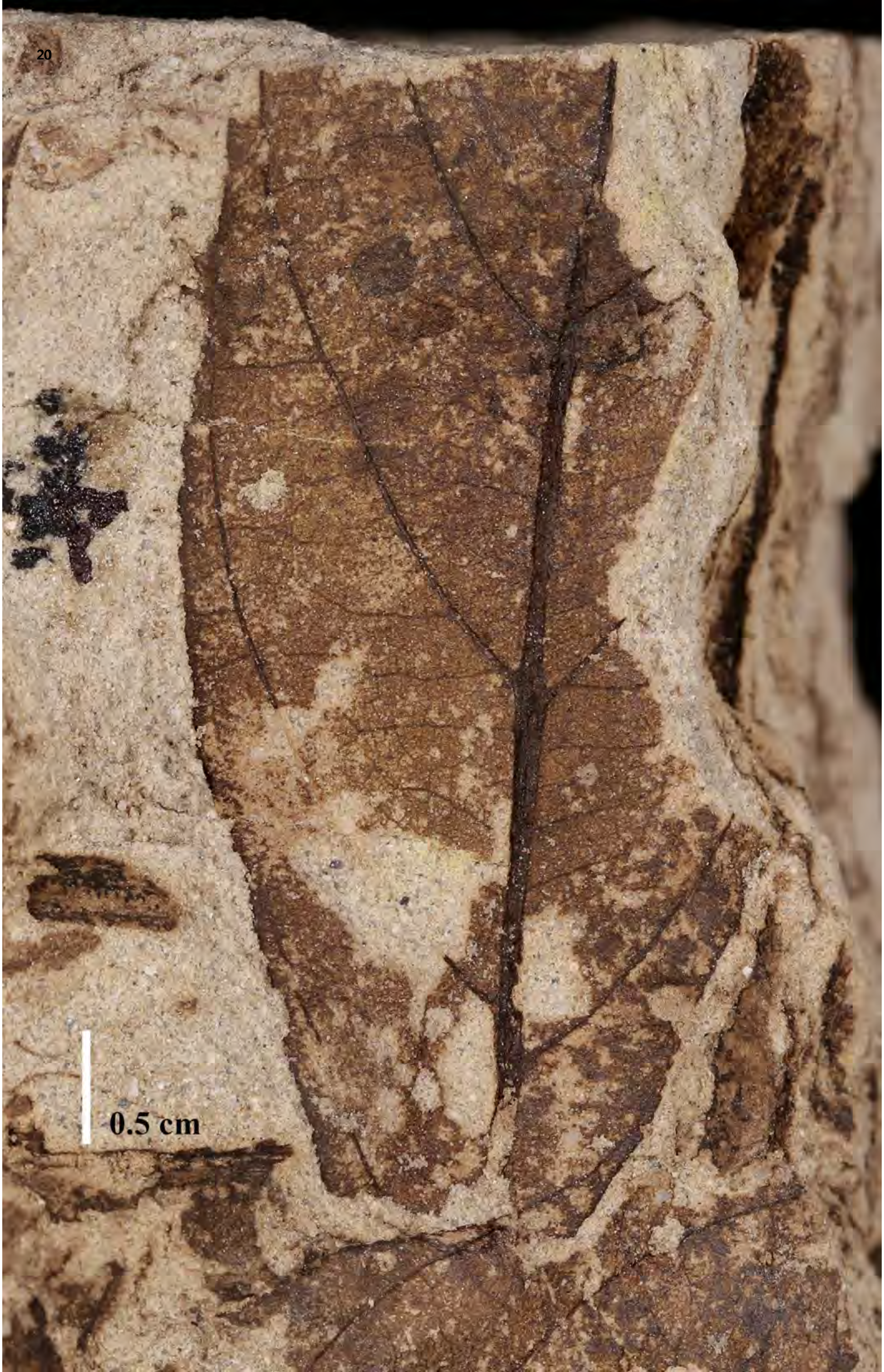
0.5 cm

MORPHOTYPE	HB178	NAME	

GENERAL

MAJOR GROUP: DIC — INFERED FAMILY: Moraceae — ORGAN TYPE: Leaf

EXEMPLAR LOC.: Hanna Basin, WY — EXEMPLAR: EC1508_1, 1701_17B — OTHERS LOC.:

DIAGNOSTIC FEATURES OF MORPHOTYPE: 3° veins are closely spaced and alternate between mixed opp/alt percurrent. The veins located in the middle of the leaf are dominated by opposite purcurrent and become alternate toward the exterior of the leaf. 2° veins are eucamptoromous which is unlike other morphotypes with mixed fameworks and the base is symmetrical.

MQI:

LEAF ATTACHMENT, ORGANIZATION, SIZE, SHAPE AND PETIOLE FEATURES

LEAF ATTACHMENT:	Petiolate	LEAFLET ORGANIZATION:	Not Preserved
LEAF ARRANGEMENT:	Not Preserved	LEAFLET ATTACHMENT:	np
LEAF ORGANIZATION:	Not Preserved	PETIOLE ATTACHMENT.:	Not Preserved
BLADE SIZE:	Nanophyll-Mesophyll	PETIOLE BASE:	Not Preserved
BLADE SHAPE:	Ovate	PETIOLE GLANDS:.	NP
BLADE RATIO L:W:	1:1	PETIOLE X-SECTION:	NP
LOBATION:	Unlobed	MARGIN TYPE	Untoothed
BASE ASYMMETRY:	Symmetrical	MEDIAL SYMMETRY:	Symmetrical

BASE SHAPE:	Concave	APEX SHAPE:	Not preserved	Special Margin Features:	Not Preserved
BASE ANGLE:	Acute	APEX ANGLE:	Not preserved	Terminal apex features:	Not Preserved

PRIMARY & SECONDARY VEIN FEATURES

		NAKED BASAL VEINS:	Absent
PRIMARY VENATION:	Basal actinodromous	N° BASAL VEINS:	3 — Interior 2°: Absent
MAJOR 2° Framework:	Eucamptodromous	MINOR-2° Course:	Semicraspedodromous
MAJOR 2° Attachment:	Decurrent	MAJOR 2° VEIN ANGLE:	Uniform
MAJOR 2° SPACING:	Decreasing proximally	AGROPHIC VEINS:	Simple
INTER-2° proximal course:		INTER-2° VEIN LENGTH:	
INTER-2° distal course:		INTER-2° FREQUENCY:	

TERTIARY VEIN FEATURES

INTERCOSTAL 3°:	Mixed opp/alt percurrent	PERIMARGINAL VEINS:	None
EPIMEDIAL 3°:	Opposite percurrent	INTER-3° ANGLE TO 1°:	Perpendicular to midvein
ADMEDIAL COURSE:	Perpendicular to midvein	EXTERIOR 3° COURSE:	Terminating at the margin
EXMEDIAL COURSE:	Basiflexed	INTERCOSTAL 3° VARIABILITY:	Decreasing exmedially

FOURTH & HIGHER ORDER VEIN FEATURES

4° VEIN FABRIC:	Alternate percurrent	VEINLETS -F/E/V/s:	Not Preserved
5° VEIN FABRIC:	Irregular reticulate	TYPE OF F.E.V. BRANCHING:	n.p
MARGINAL VENATION:	Not Preserved	F.E.V.s TERMINATIONS:	Not Preserved
AREOLATION:	Poorly developed	LEAF RANK:	

TOOTH FEATURES

ORDERS OF TEETH:		No TEETH/cm:		TEETH GLANDULARITY:	
TOOTH SHAPE:		TOOTH SPACING:			
PRINCIPAL VEIN:		SINUS SHAPE:			
ACCESSORY VEIN COURSE:		PRINCIPAL VEIN TERM.:			

NOTES

Morphotype sheet completed using specimens: EC1508-1, 1704-44 and 1704-14C

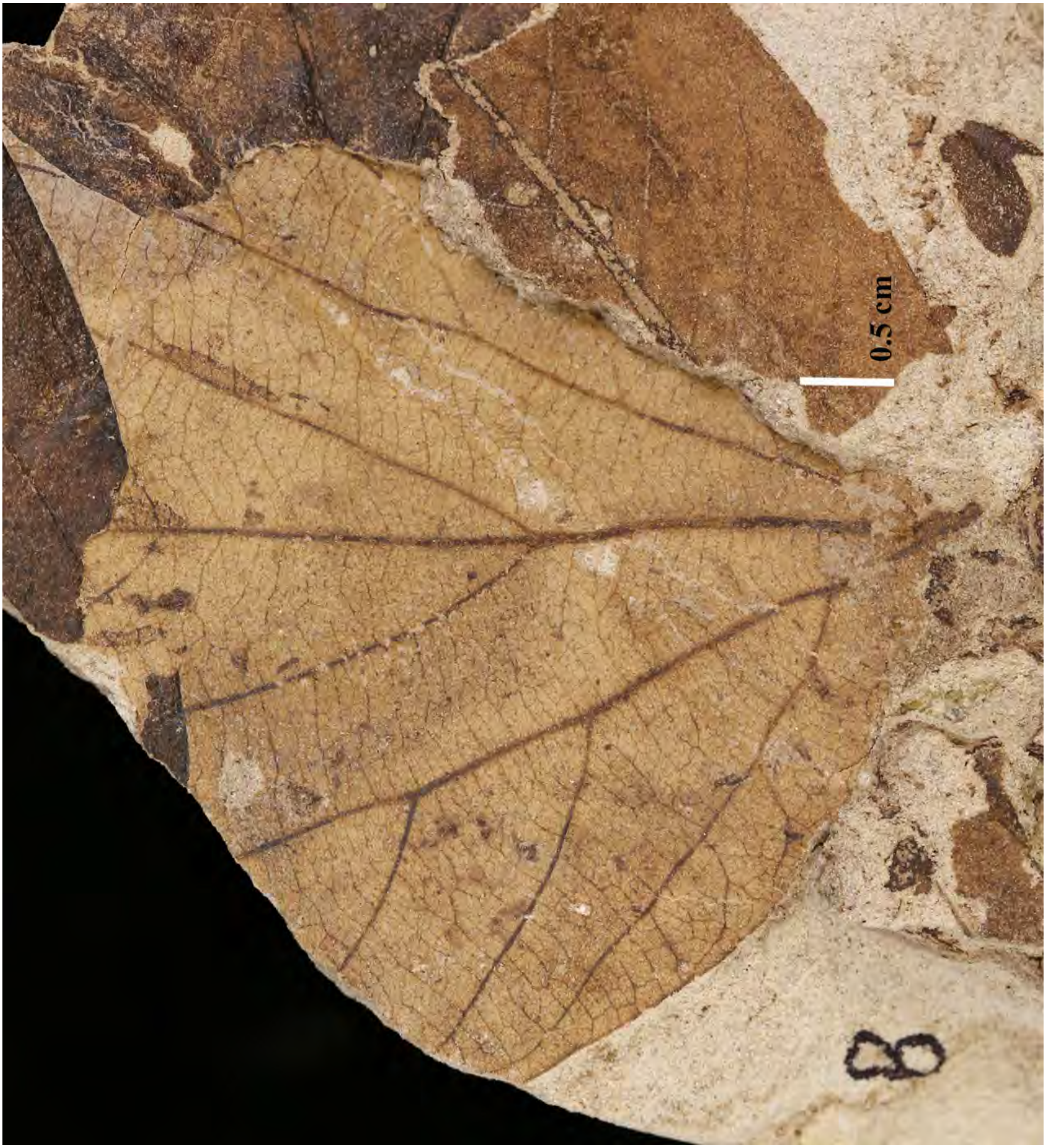
0.5 cm

MORPHOTYPE	HB179	NAME	

GENERAL

MAJOR GROUP	DIC	INFERED FAMILY		ORGAN TYPE	Leaf
EXEMPLAR LOC.	LAS 1714	EXEMPLAR	1713-14	OTHERS LOC.	LAS 1713, LAS 1715

DIAGNOSTIC FEATURES OF MORPHOTYPE: 2° framework is strongly brochidodromous and the major vein angle decreases towards the base. This is unlike any other morphotype at these sites. Lastly, the base is asymetrical and the 5° vein fabric is freely ramifying .

MQI:

LEAF ATTACHMENT, ORGANIZATION, SIZE, SHAPE AND PETIOLE FEATURES

LEAF ATTACHMENT:	Petiolate	LEAFLET ORGANIZATION:	Not Preserved
LEAF ARRANGEMENT:	Not Preserved	LEAFLET ATTACHMENT:	np
LEAF ORGANIZATION:	Not Preserved	PETIOLE ATTACHMENT.:	Not Preserved
BLADE SIZE:	Microphyll-Notophyll	PETIOLE BASE:	Not Preserved
BLADE SHAPE:	Ovate	PETIOLE GLANDS:.	Not Preserved
BLADE RATIO L:W:	2:1	PETIOLE X-SECTION:	Not Preserved
LOBATION:	Unlobed	MARGIN TYPE	Untoothed
BASE ASYMMETRY:	Basal width asymmetrical	MEDIAL SYMMETRY:	Asymetrical

BASE SHAPE:	Concave	APEX SHAPE:	Acuminate	Special Margin Features:	Sinuous
BASE ANGLE:	Not Preserved	APEX ANGLE:	Acute	Terminal apex features:	Not Preserved

PRIMARY & SECONDARY VEIN FEATURES

		NAKED BASAL VEINS:	Absent
PRIMARY VENATION:	Pinnate	N° BASAL VEINS:	1 (Interior 2°: Absent)
MAJOR 2° Framework:	Simple brochidodromous	MINOR-2° Course:	
MAJOR 2° Attachment:	Decurrent	MAJOR 2° VEIN ANGLE:	Smoothly decreasing toward base
MAJOR 2° SPACING:	Gradually increasing proximally	AGROPHIC VEINS:	Absent
INTER-2° proximal course:		INTER-2° VEIN LENGTH:	
INTER-2° distal course:		INTER-2° FREQUENCY:	

TERTIARY VEIN FEATURES

INTERCOSTAL 3°:	Opposite percurrent - sinuous	PERIMARGINAL VEINS:	Not Preserved
EPIMEDIAL 3°:	Opposite percurrent	INTER-3° ANGLE TO 1°:	Acute to midvein
ADMEDIAL COURSE:	Not Preserved	EXTERIOR 3° COURSE:	Not Preserved
EXMEDIAL COURSE:	Basiflexed	INTERCOSTAL 3° VARIABILITY:	Not Preserved

FOURTH & HIGHER ORDER VEIN FEATURES

4° VEIN FABRIC:	Opposite percurrent	VEINLETS -F/E/V/s:	Not Preserved
5° VEIN FABRIC:	Freely ramifying	TYPE OF F.E.V. BRANCHING:	n.p
MARGINAL VENATION:	Not Preserved	F.E.V.s TERMINATIONS:	Not Preserved
AREOLATION:	Poorly developed	LEAF RANK:	

TOOTH FEATURES

ORDERS OF TEETH:		No TEETH/cm:		TEETH GLANDULARITY:	
TOOTH SHAPE:		TOOTH SPACING:			
PRINCIPAL VEIN:		SINUS SHAPE:			
ACCESSORY VEIN COURSE:		PRINCIPAL VEIN TERM.:			

NOTES

Specimens used to complete morphosheet: 1701-97, 1713-1000 and 1713-14

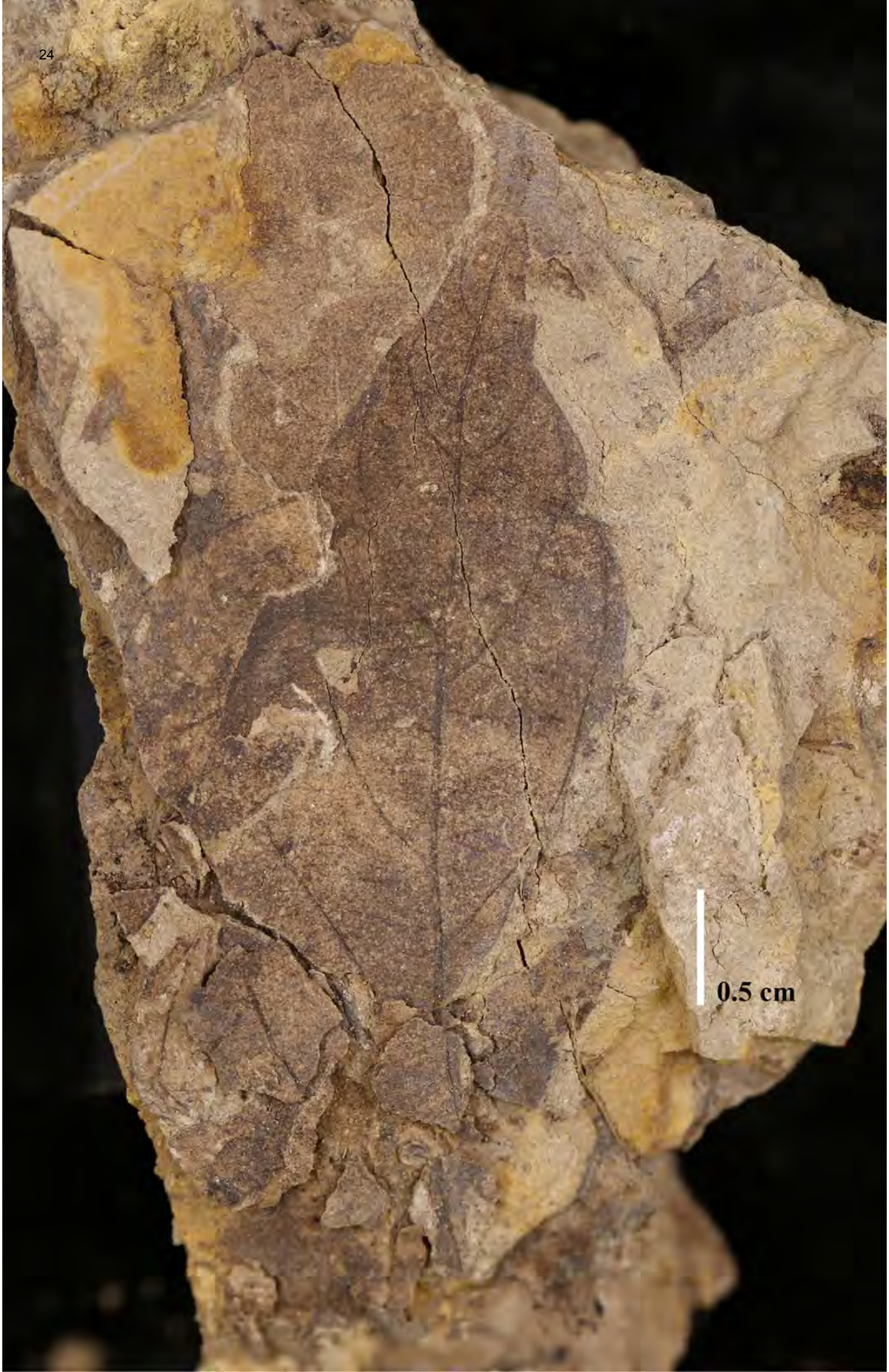
0.5 cm

MORPHOTYPE HB180 | NAME

GENERAL

MAJOR GROUP: DIC
INFERED FAMILY:
ORGAN TYPE: Leaf
EXEMPLAR LOC.: LAS 1701
EXEMPLAR: 1701-45
OTHERS LOC.: LAS 1713
DIAGNOSTIC FEATURES OF MORPHOTYPE: Weakly brochidodromous and eucamptodromous however, the 3° are opposite percurrent-straight and obtuse to midvein. Also the 2° spacing gradually increases proximally and is acute compared to HB176.
MQI:

LEAF ATTACHMENT, ORGANIZATION, SIZE, SHAPE AND PETIOLE FEATURES

LEAF ATTACHMENT: Petiolate
LEAFLET ORGANIZATION: Not Preserved
LEAF ARRANGEMENT: Not Preserved
LEAFLET ATTACHMENT: np
LEAF ORGANIZATION: Not Preserved
PETIOLE ATTACHMENT.: Not Preserved
BLADE SIZE: Microphyll-Mesophyll
PETIOLE BASE: Not Preserved
BLADE SHAPE: Ovate
PETIOLE GLANDS:. Not Preserved
BLADE RATIO L:W: 2:1
PETIOLE X-SECTION: Not Preserved
LOBATION: Unlobed
MARGIN TYPE: Untoothed
BASE ASYMMETRY: Symmetrical
MEDIAL SYMMETRY: Symmetrical
BASE SHAPE: Convex
APEX SHAPE: Not preserved
Special Margin Features: Not Preserved
BASE ANGLE: Acute
APEX ANGLE: Not preserved
Terminal apex features: Not Preserved

PRIMARY & SECONDARY VEIN FEATURES

NAKED BASAL VEINS: Not Preserved
PRIMARY VENATION: Pinnate
N° BASAL VEINS: 1
Interior 2°: Absent
MAJOR 2° Framework: Mixed framework
MINOR-2° Course:
MAJOR 2° Attachment: Decurrent
MAJOR 2° VEIN ANGLE: Smoothly increasing toward base
MAJOR 2° SPACING: Gradually increasing proximally
AGROPHIC VEINS: Absent
INTER-2° proximal course:
INTER-2° VEIN LENGTH:
INTER-2° distal course:
INTER-2° FREQUENCY:

TERTIARY VEIN FEATURES

INTERCOSTAL 3°: Opposite percurrent - straight
PERIMARGINAL VEINS: Not Preserved
EPIMEDIAL 3°: Opposite percurrent
INTER-3° ANGLE TO 1°: Obtuse to midvein
ADMEDIAL COURSE: Not Preserved
EXTERIOR 3° COURSE: Not Preserved
EXMEDIAL COURSE: Not Preserved
INTERCOSTAL 3° VARIABILITY: Consistent

FOURTH & HIGHER ORDER VEIN FEATURES

4° VEIN FABRIC: Not Preserved
VEINLETS -F/E/V/s: Not Preserved
5° VEIN FABRIC: Not Preserved
TYPE OF F.E.V. BRANCHING: n.p
MARGINAL VENATION: Not Preserved
F.E.V.s TERMINATIONS: Not Preserved
AREOLATION: Not Preserved
LEAF RANK:

TOOTH FEATURES

ORDERS OF TEETH:
No TEETH/cm:
TEETH GLANDULARITY:
TOOTH SHAPE:
TOOTH SPACING:
PRINCIPAL VEIN:
SINUS SHAPE:
ACCESSORY VEIN COURSE:
PRINCIPAL VEIN TERM.:

NOTES

Specimen used to complete morphosheet is: 1701-45

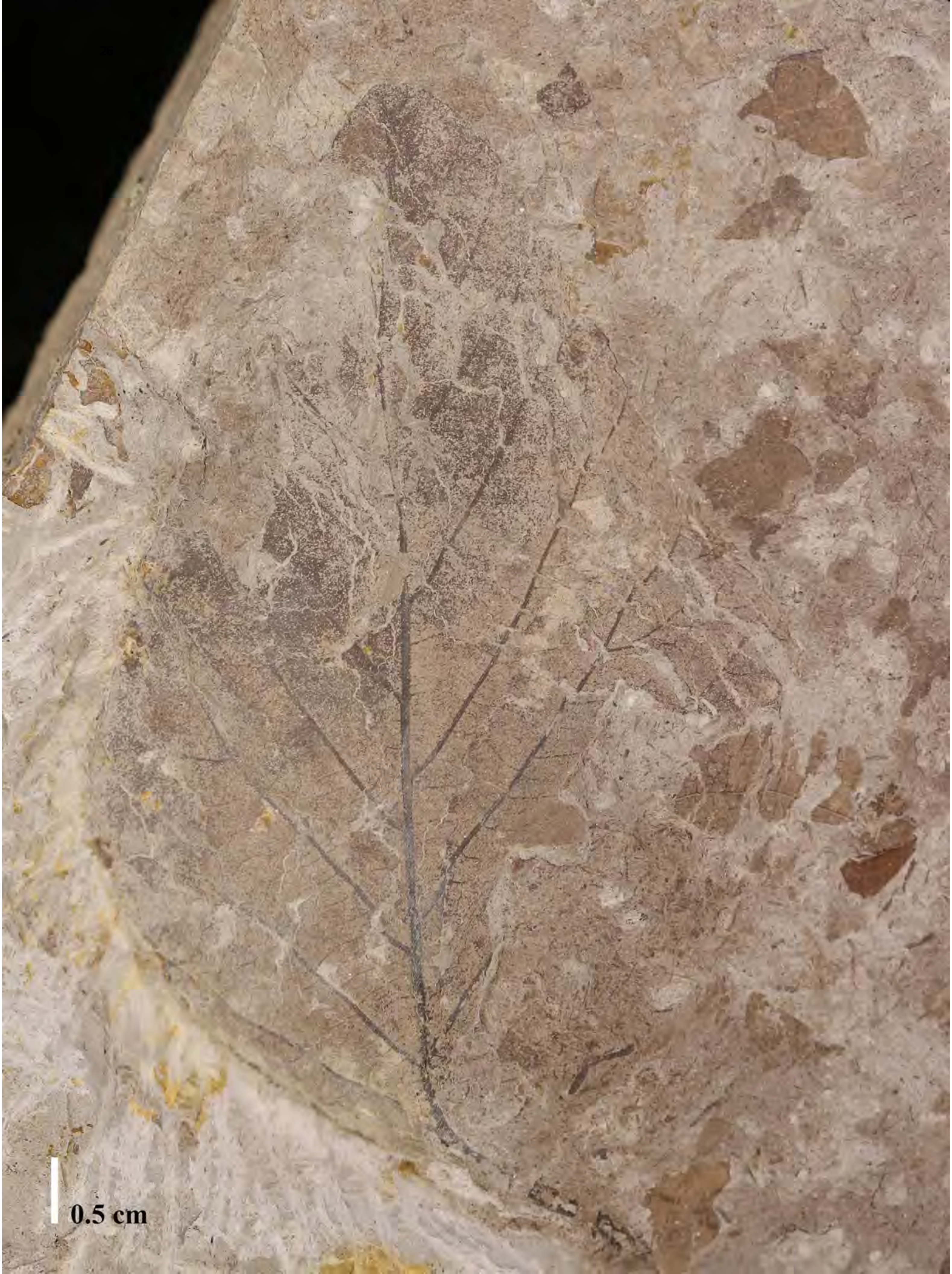
0.5 cm

MORPHOTYPE	HB181	NAME	

GENERAL

MAJOR GROUP	DIC	INFERED FAMILY		ORGAN TYPE	Leaf
EXEMPLAR LOC.	1704	EXEMPLAR	1704-13C	OTHERS LOC.	

DIAGNOSTIC FEATURES OF MORPHOTYPE: Very poorly preserved however, interesting morphology!
4 primary veins preserved and agrophic veins however no other features are preserved.
Due to the primary veins, this leaf can not be put in any other morphospecies.

MQI:

LEAF ATTACHMENT, ORGANIZATION, SIZE, SHAPE AND PETIOLE FEATURES

LEAF ATTACHMENT:	Not Preserved	LEAFLET ORGANIZATION:	Not Preserved
LEAF ARRANGEMENT:	Not Preserved	LEAFLET ATTACHMENT:	np
LEAF ORGANIZATION:	Not Preserved	PETIOLE ATTACHMENT.:	Not Preserved
BLADE SIZE:	Notophyll	PETIOLE BASE:	Not Preserved
BLADE SHAPE:	Not Preserved	PETIOLE GLANDS:.	Not Preserved
BLADE RATIO L:W:	Not Preserved	PETIOLE X-SECTION:	Not Preserved
LOBATION:	Not Preserved	MARGIN TYPE	Untoothed
BASE ASYMMETRY:	Not Preserved	MEDIAL SYMMETRY:	Not Preserved

BASE SHAPE:	Not Preserved	APEX SHAPE:	Not preserved	Special Margin Features:	Not Preserved
BASE ANGLE:	Not Preserved	APEX ANGLE:	Not preserved	Terminal apex features:	Not Preserved

PRIMARY & SECONDARY VEIN FEATURES

		NAKED BASAL VEINS:	Not Preserved
PRIMARY VENATION:	Some kind of Palmate	N° BASAL VEINS:	4 — Interior 2°: NP
MAJOR 2° Framework:	Semicraspedodromous	MINOR-2° Course:	Not Preserved
MAJOR 2° Attachment:	Decurrent	MAJOR 2° VEIN ANGLE:	Not Preserved
MAJOR 2° SPACING:	Not Preserved	AGROPHIC VEINS:	Simple
INTER-2° proximal course:		INTER-2° VEIN LENGTH:	
INTER-2° distal course:		INTER-2° FREQUENCY:	

TERTIARY VEIN FEATURES

INTERCOSTAL 3°:	Not Preserved	PERIMARGINAL VEINS:	Not Preserved
EPIMEDIAL 3°:	Not preserved	INTER-3° ANGLE TO 1°:	Not Preserved
ADMEDIAL COURSE:	Not Preserved	EXTERIOR 3° COURSE:	Not Preserved
EXMEDIAL COURSE:	Not Preserved	INTERCOSTAL 3° VARIABILITY:	Not Preserved

FOURTH & HIGHER ORDER VEIN FEATURES

4° VEIN FABRIC:	Not Preserved	VEINLETS -F/E/V/s:	Not Preserved
5° VEIN FABRIC:	Not Preserved	TYPE OF F.E.V. BRANCHING:	n.p
MARGINAL VENATION:	Not Preserved	F.E.V.s TERMINATIONS:	Not Preserved
AREOLATION:	Not Preserved	LEAF RANK:	

TOOTH FEATURES

ORDERS OF TEETH:		No TEETH/cm:		TEETH GLANDULARITY:	
TOOTH SHAPE:		TOOTH SPACING:			
PRINCIPAL VEIN:		SINUS SHAPE:			
ACCESSORY VEIN COURSE:		PRINCIPAL VEIN TERM.:			

NOTES

Specimen used to complete morphosheet: 1704-13C

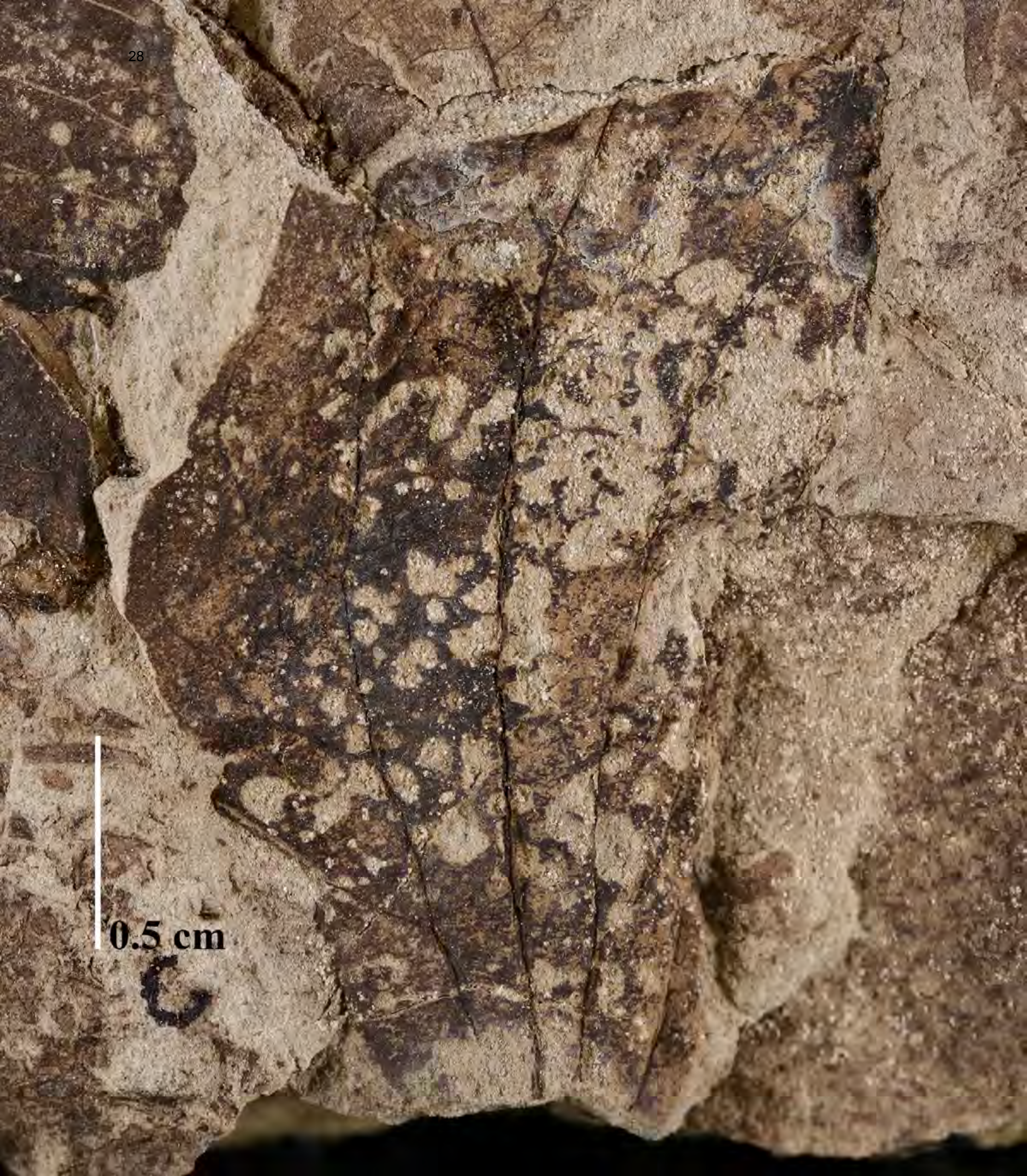
0.5 cm
C

MORPHOTYPE	HB182	NAME	

GENERAL

MAJOR GROUP	DIC	INFERED FAMILY		ORGAN TYPE	Leaf
EXEMPLAR LOC.	1710	EXEMPLAR	1710-26	OTHERS LOC.	

DIAGNOSTIC FEATURES OF MORPHOTYPE: Very regularly spaced 2° veins with consistent acute angels and the framework is eucamptodromous. The acute angel measures at 30°.

MQI:

LEAF ATTACHMENT, ORGANIZATION, SIZE, SHAPE AND PETIOLE FEATURES

LEAF ATTACHMENT:	Not Preserved	LEAFLET ORGANIZATION:	Not Preserved
LEAF ARRANGEMENT:	Not Preserved	LEAFLET ATTACHMENT:	np
LEAF ORGANIZATION:	Not Preserved	PETIOLE ATTACHMENT.:	Not Preserved
BLADE SIZE:	Microphyll-Mesophyll	PETIOLE BASE:	Not Preserved
BLADE SHAPE:	Ovate	PETIOLE GLANDS:.	Not Preserved
BLADE RATIO L:W:	2:1	PETIOLE X-SECTION:	Not Preserved
LOBATION:	Unlobed	MARGIN TYPE	Untoothed
BASE ASYMMETRY:	Not Preserved	MEDIAL SYMMETRY:	Symmetrical

BASE SHAPE:	Not Preserved	APEX SHAPE:	Acuminate	Special Margin Features:	Not Preserved
BASE ANGLE:	Not Preserved	APEX ANGLE:	Acute	Terminal apex features:	Not Preserved

PRIMARY & SECONDARY VEIN FEATURES

		NAKED BASAL VEINS:	Not Preserved
PRIMARY VENATION:	Pinnate	N° BASAL VEINS:	1 — Interior 2°: Absent
MAJOR 2° Framework:	Eucamptodromous	MINOR-2° Course:	
MAJOR 2° Attachment:	Decurrent	MAJOR 2° VEIN ANGLE:	Smoothly increasing toward base
MAJOR 2° SPACING:	Regular	AGROPHIC VEINS:	Absent
INTER-2° proximal course:		INTER-2° VEIN LENGTH:	
INTER-2° distal course:		INTER-2° FREQUENCY:	

TERTIARY VEIN FEATURES

INTERCOSTAL 3°:	Mixed opp/alt percurrent	PERIMARGINAL VEINS:	Not Preserved
EPIMEDIAL 3°:	Mixed opp/alt percurrent	INTER-3° ANGLE TO 1°:	Acute to midvein
ADMEDIAL COURSE:	Perpendicular to midvein	EXTERIOR 3° COURSE:	Not Preserved
EXMEDIAL COURSE:	Basiflexed	INTERCOSTAL 3° VARIABILITY:	Consistent

FOURTH & HIGHER ORDER VEIN FEATURES

4° VEIN FABRIC:	Mixed percurrent	VEINLETS -F/E/V/s:	Not Preserved
5° VEIN FABRIC:	Regular reticulate	TYPE OF F.E.V. BRANCHING:	n.p
MARGINAL VENATION:	Not Preserved	F.E.V.s TERMINATIONS:	Not Preserved
AREOLATION:	Well developed	LEAF RANK:	

TOOTH FEATURES

ORDERS OF TEETH:		No TEETH/cm:		TEETH GLANDULARITY:	
TOOTH SHAPE:		TOOTH SPACING:			
PRINCIPAL VEIN:		SINUS SHAPE:			
ACCESSORY VEIN COURSE:		PRINCIPAL VEIN TERM.:			

NOTES

Specimens used to complete morphotype sheet: 1710-6B and 1710-207

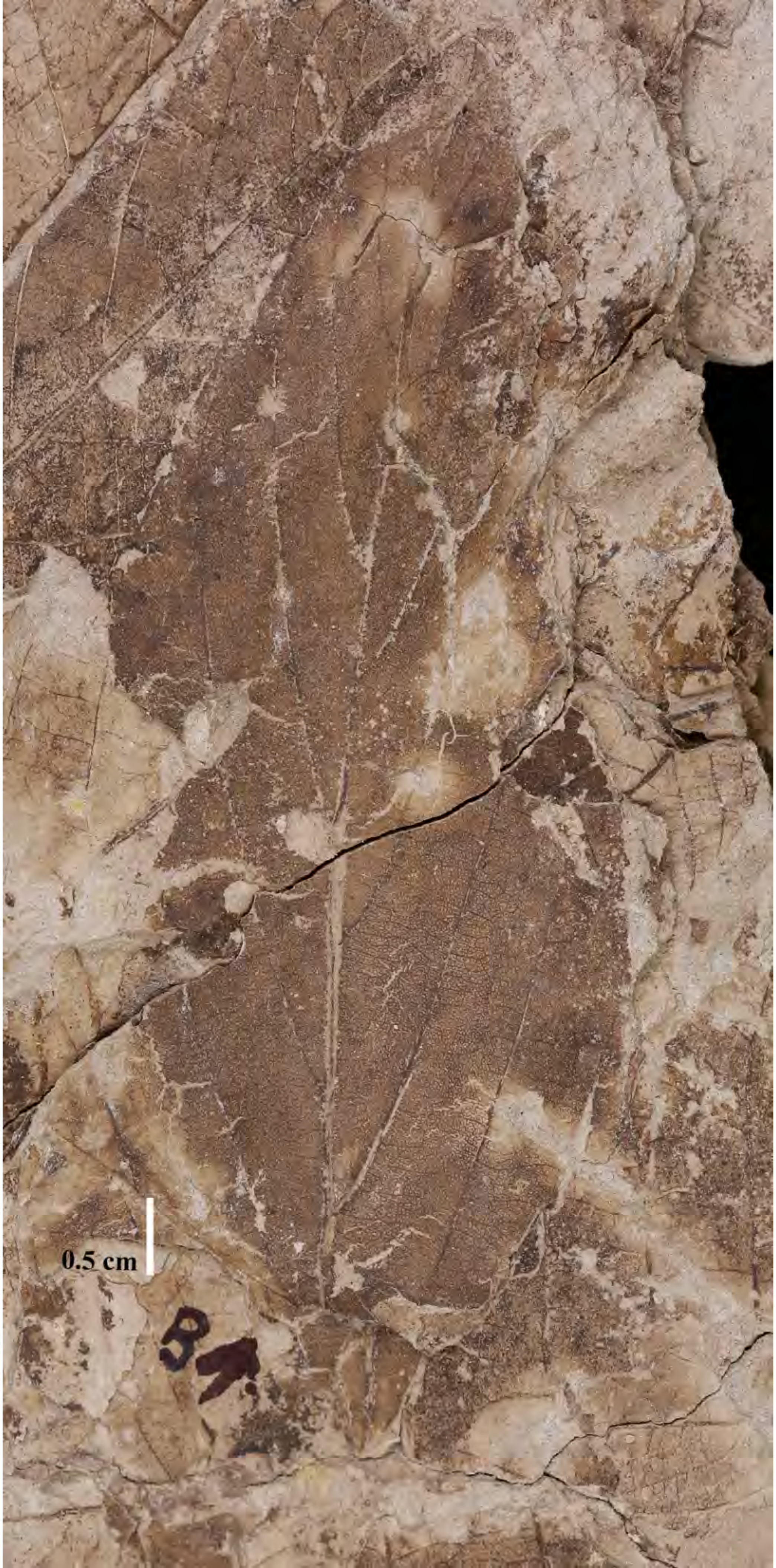
0.5 cm
B1

MORPHOTYPE	HB183	NAME	

GENERAL

MAJOR GROUP	DIC	INFERED FAMILY		ORGAN TYPE	Leaf
EXEMPLAR LOC.	LAS 1701	EXEMPLAR	1704-47A	OTHERS LOC.	LAS 1706

DIAGNOSTIC FEATURES OF MORPHOTYPE: Thick petiole and midvein that decreases in width apically. Midevein at base is 2x thicker than at apex. Unlike Averrhoites, HB183 doesn't have intersec 2° veins and the preservation on this morphotype is superior to Averrhoites. The 2° framewo is weakly brochidodromous with irregular spacing throughout the leaf.

MQI:

LEAF ATTACHMENT, ORGANIZATION, SIZE, SHAPE AND PETIOLE FEATURES

LEAF ATTACHMENT:	Petiolate	LEAFLET ORGANIZATION:	Not Preserved
LEAF ARRANGEMENT:	Not Preserved	LEAFLET ATTACHMENT:	np
LEAF ORGANIZATION:	Not Preserved	PETIOLE ATTACHMENT.:	Not Preserved
BLADE SIZE:	Microphyll-Mesophyll	PETIOLE BASE:	Not Preserved
BLADE SHAPE:	Elliptic	PETIOLE GLANDS:.	Not Preserved
BLADE RATIO L:W:	2:1	PETIOLE X-SECTION:	Not Preserved
LOBATION:	Unlobed	MARGIN TYPE	Untoothed
BASE ASYMMETRY:	Basal width asymmetrical	MEDIAL SYMMETRY:	Symmetrical

BASE SHAPE:	Concave	APEX SHAPE:	Not preserved	Special Margin Features:	Not Preserved
BASE ANGLE:	Acute	APEX ANGLE:	Not preserved	Terminal apex features:	Not Preserved

PRIMARY & SECONDARY VEIN FEATURES

		NAKED BASAL VEINS:	Not Preserved
PRIMARY VENATION:	Pinnate	N° BASAL VEINS:	1
		Interior 2°:	Absent
MAJOR 2° Framework:	Simple brochidodromous	MINOR-2° Course:	
MAJOR 2° Attachment:	Decurrent	MAJOR 2° VEIN ANGLE:	Inconsistent
MAJOR 2° SPACING:	Irregular	AGROPHIC VEINS:	Absent
INTER-2° proximal course:		INTER-2° VEIN LENGTH:	
INTER-2° distal course:		INTER-2° FREQUENCY:	

TERTIARY VEIN FEATURES

INTERCOSTAL 3°:	Opposite percurrent - convex	PERIMARGINAL VEINS:	None
EPIMEDIAL 3°:	Opposite percurrent	INTER-3° ANGLE TO 1°:	Acute to midvein
ADMEDIAL COURSE:	Acute to midvein	EXTERIOR 3° COURSE:	Terminating at the margin
EXMEDIAL COURSE:	Basiflexed	INTERCOSTAL 3° VARIABILITY:	Inconsistent

FOURTH & HIGHER ORDER VEIN FEATURES

4° VEIN FABRIC:	Not Preserved	VEINLETS -F/E/V/s:	Not Preserved
5° VEIN FABRIC:	Not Preserved	TYPE OF F.E.V. BRANCHING:	n.p
MARGINAL VENATION:	Not Preserved	F.E.V.s TERMINATIONS:	Not Preserved
AREOLATION:	Not Preserved	LEAF RANK:	

TOOTH FEATURES

ORDERS OF TEETH:		No TEETH/cm:		TEETH GLANDULARITY:	
TOOTH SHAPE:		TOOTH SPACING:			
PRINCIPAL VEIN:		SINUS SHAPE:			
ACCESSORY VEIN COURSE:		PRINCIPAL VEIN TERM.:			

NOTES

Specimens used to complete morphosheet: 1704-47A and 1704-47C

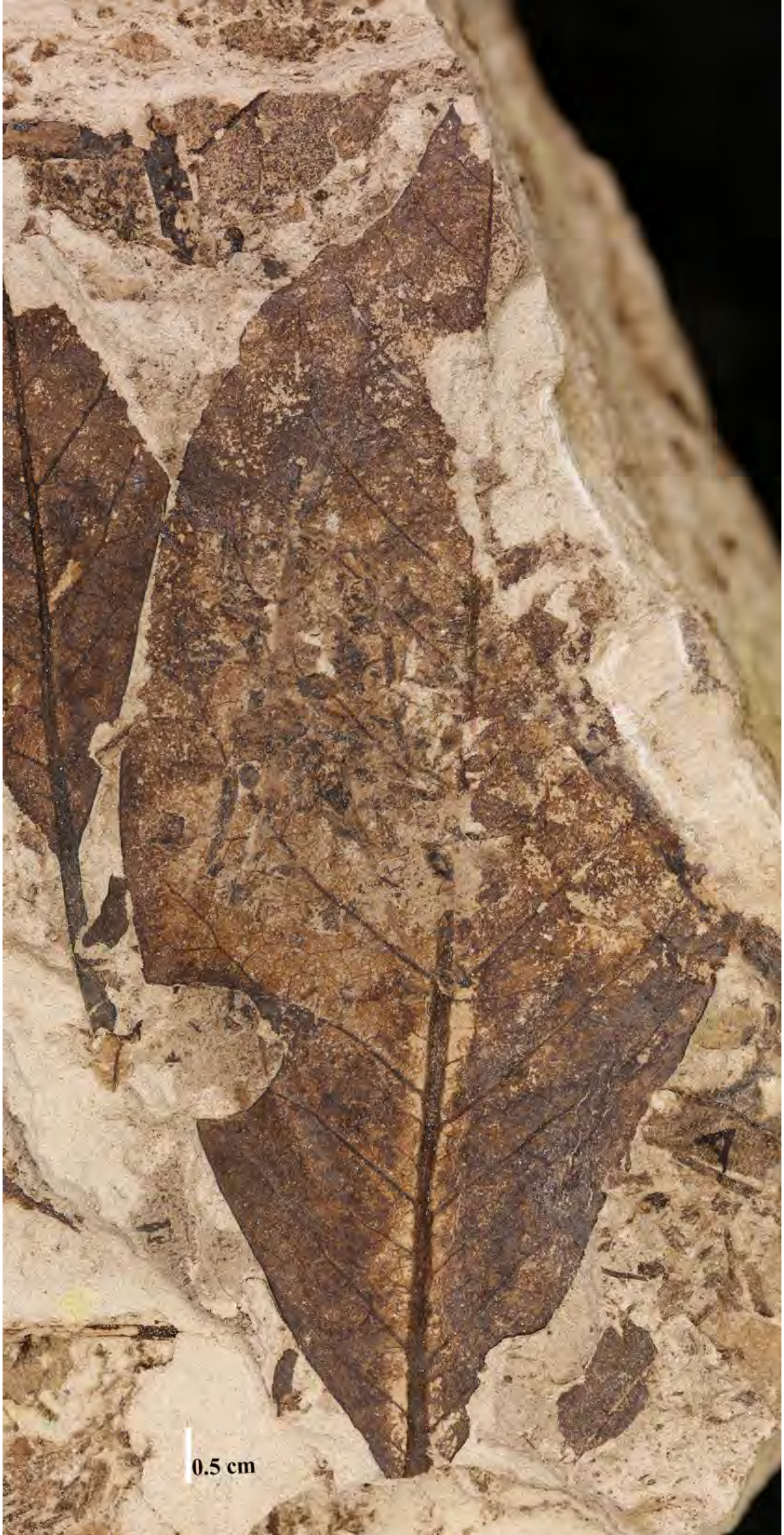
0.5 cm

MORPHOTYPE		NAME	*Macginitiea gracilis*

GENERAL

MAJOR GROUP	DIC	INFERED FAMILY	Platanaceae	ORGAN TYPE	Leaf
EXEMPLAR LOC.	1703	EXEMPLAR	1703-9A, 1703-1	OTHERS LOC.	

DIAGNOSTIC FEATURES OF MORPHOTYPE

Untoothed, palmate lobed leaf with symetrical base. This is the only palmate lobed leaf we have across stratigraphic levels. Agrophic veins are absentand the secondary vein spacing decreases proximally.

MQI:

LEAF ATTACHMENT, ORGANIZATION, SIZE, SHAPE AND PETIOLE FEATURES

LEAF ATTACHMENT:	Petiolate	LEAFLET ORGANIZATION:	Not Preserved
LEAF ARRANGEMENT:	Not Preserved	LEAFLET ATTACHMENT:	np
LEAF ORGANIZATION:	Not Preserved	PETIOLE ATTACHMENT.:	Not Preserved
BLADE SIZE:	Notophyll-Macrophyll	PETIOLE BASE:	Not Preserved
BLADE SHAPE:	Wide elliptic	PETIOLE GLANDS:.	NP
BLADE RATIO L:W:	1:5-1	PETIOLE X-SECTION:	NP
LOBATION:	Palmately lobed	MARGIN TYPE	Untoothed
BASE ASYMMETRY:	Symmetrical	MEDIAL SYMMETRY:	Symmetrical

BASE SHAPE:	Concave	APEX SHAPE:	Not preserved	Special Margin Features:	Not Preserved
BASE ANGLE:	Obtuse	APEX ANGLE:	Not preserved	Terminal apex features:	Not Preserved

PRIMARY & SECONDARY VEIN FEATURES

		NAKED BASAL VEINS:	Absent
PRIMARY VENATION:	Basal palinactinodromous	N° BASAL VEINS:	3 (Interior 2°s: Present)
MAJOR 2° Framework:	Semicraspedodromous	MINOR-2° Course:	
MAJOR 2° Attachment:	Decurrent	MAJOR 2° VEIN ANGLE:	Smoothly decreasing toward base
MAJOR 2° SPACING:	Gradually increasing proximally	AGROPHIC VEINS:	Absent
INTER-2° proximal course:	Perpendicular to midvein	INTER-2° VEIN LENGTH:	<50% of subjacent 2°
INTER-2° distal course:	Perpendicular to major 2°	INTER-2° FREQUENCY:	>1 per intercostal area

TERTIARY VEIN FEATURES

INTERCOSTAL 3°:	Mixed opp/alt percurrent	PERIMARGINAL VEINS:	None
EPIMEDIAL 3°:	Opposite percurrent	INTER-3° ANGLE TO 1°:	Obtuse to midvein
ADMEDIAL COURSE:	Obtuse to midvein	EXTERIOR 3° COURSE:	Not Preserved
EXMEDIAL COURSE:	Basiflexed	INTERCOSTAL 3° VARIABILITY:	Consistent

FOURTH & HIGHER ORDER VEIN FEATURES

4° VEIN FABRIC:	Alternate percurrent	VEINLETS -F/E/V/s:	Not Preserved
5° VEIN FABRIC:	Regular reticulate	TYPE OF F.E.V. BRANCHING:	n.p
MARGINAL VENATION:	Not Preserved	F.E.V.s TERMINATIONS:	Not Preserved
AREOLATION:	Moderately developed	LEAF RANK:	

TOOTH FEATURES

ORDERS OF TEETH:		No TEETH/cm:		TEETH GLANDULARITY:	
TOOTH SHAPE:		TOOTH SPACING:			
PRINCIPAL VEIN:		SINUS SHAPE:			
ACCESSORY VEIN COURSE:		PRINCIPAL VEIN TERM.:			

NOTES

Specimens used to complete sheet are: 1703-9A and 1703-1

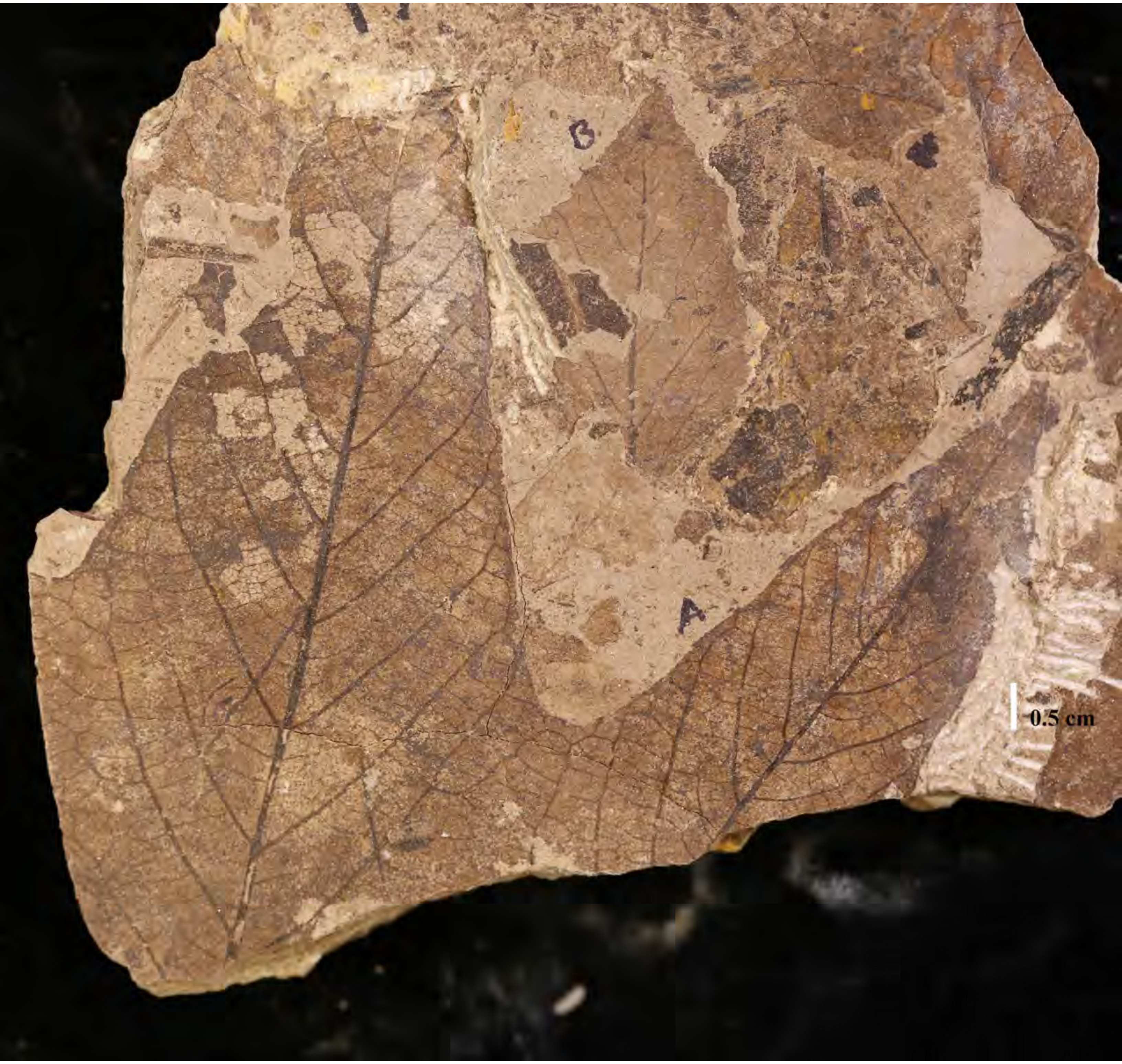
B
A
0.5 cm

MORPHOTYPE		NAME	*Platanites raynoldsii* (Palmate)

GENERAL

MAJOR GROUP	DIC	INFERED FAMILY	Platanaceae	ORGAN TYPE	Leaf
EXEMPLAR LOC.	LAS1701	EXEMPLAR	1701-62B	OTHERS LOC.	

DIAGNOSTIC FEATURES OF MORPHOTYPE: Serrate teeth that resemble hooks with non-specific glands. Teeth only have one order and approximately 2 teeth/cm. When preserved, base is concave-convex. Palmate version of Platanites is suprabasal actinodromous

MQI:

LEAF ATTACHMENT, ORGANIZATION, SIZE, SHAPE AND PETIOLE FEATURES

LEAF ATTACHMENT:	Petiolate	LEAFLET ORGANIZATION:	Not Preserved
LEAF ARRANGEMENT:	Not Preserved	LEAFLET ATTACHMENT:	np
LEAF ORGANIZATION:	Not Preserved	PETIOLE ATTACHMENT.:	Not Preserved
BLADE SIZE:	Microphyll-Mesophyll	PETIOLE BASE:	Not Preserved
BLADE SHAPE:	Ovate	PETIOLE GLANDS:.	Not Preserved
BLADE RATIO L:W:	2:1	PETIOLE X-SECTION:	Not Preserved
LOBATION:	Unlobed	MARGIN TYPE	Serrate
BASE ASYMMETRY:	Symmetrical	MEDIAL SYMMETRY:	Symmetrical

BASE SHAPE:	Concave-convex	APEX SHAPE:	Acuminate	Special Margin Features:	Not Preserved
BASE ANGLE:	Acute	APEX ANGLE:	Acute	Terminal apex features:	Not Preserved

PRIMARY & SECONDARY VEIN FEATURES

		NAKED BASAL VEINS:	Absent
PRIMARY VENATION:	Suprabasal actinodromous	N° BASAL VEINS:	1
		Interior 2°:	Absent
MAJOR 2° Framework:	Craspedodromous	MINOR-2° Course:	Craspedodromous
MAJOR 2° Attachment:	Decurrent	MAJOR 2° VEIN ANGLE:	Inconsistent
MAJOR 2° SPACING:	Regular	AGROPHIC VEINS:	Simple
INTER-2° proximal course:		INTER-2° VEIN LENGTH:	
INTER-2° distal course:		INTER-2° FREQUENCY:	

TERTIARY VEIN FEATURES

INTERCOSTAL 3°:	Opposite percurrent - convex	PERIMARGINAL VEINS:	None
EPIMEDIAL 3°:	Opposite percurrent	INTER-3° ANGLE TO 1°:	Obtuse to midvein
ADMEDIAL COURSE:	Perpendicular to midvein	EXTERIOR 3° COURSE:	Looped
EXMEDIAL COURSE:	Basiflexed	INTERCOSTAL 3° VARIABILITY:	Consistent

FOURTH & HIGHER ORDER VEIN FEATURES

4° VEIN FABRIC:	Mixed percurrent	VEINLETS -F/E/V/s:	Not Preserved
5° VEIN FABRIC:	Irregular reticulate	TYPE OF F.E.V. BRANCHING:	n.p
MARGINAL VENATION:	Not Preserved	F.E.V.s TERMINATIONS:	Not Preserved
AREOLATION:	Moderately developed	LEAF RANK:	

TOOTH FEATURES

ORDERS OF TEETH:	1	No TEETH/cm:	2	TEETH GLANDULARITY:	Non-specific glandular
TOOTH SHAPE:	cc/rt	TOOTH SPACING:	Regular		
PRINCIPAL VEIN:	Present	SINUS SHAPE:	Rounded		
ACCESSORY VEIN COURSE:	Straight or concave	PRINCIPAL VEIN TERM.:	Marginal, at apex		

NOTES

Specimens used to complete morhopheet are: 1701-62B, 1701-50

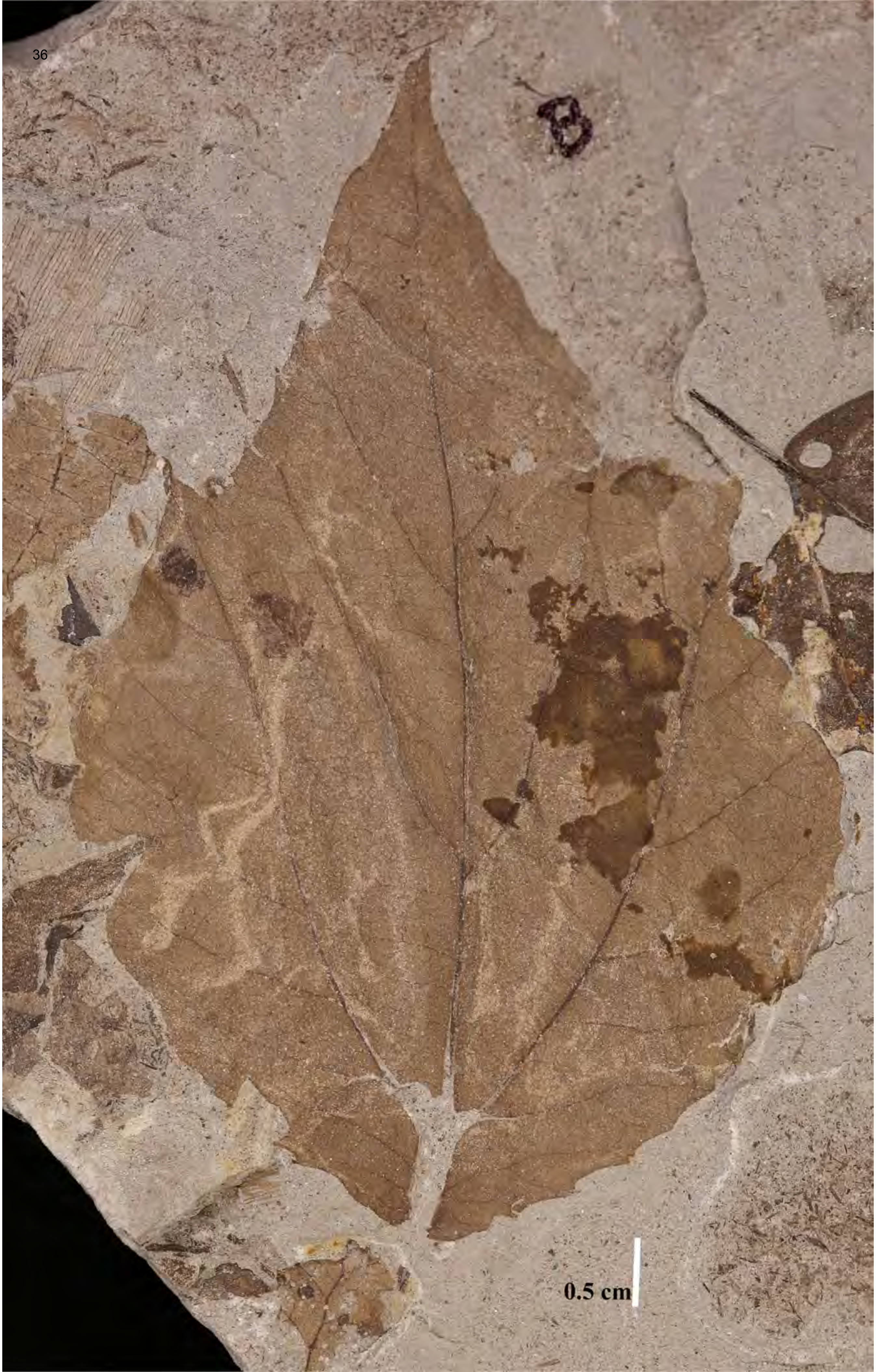
B
0.5 cm

MORPHOTYPE		NAME	*Platanites raynoldsii* (Pinnate)

GENERAL

MAJOR GROUP	DIC	INFERED FAMILY	Platanaceae	ORGAN TYPE	Leaf
EXEMPLAR LOC.	LAS1706	EXEMPLAR	1706-40C	OTHERS LOC.	LAS1701

DIAGNOSTIC FEATURES OF MORPHOTYPE: Serrate teeth that resemble hooks with non-specific glands. Teeth only have one order and approximately 2 teeth/cm. When base is preserved, it is concave-convex.

MQI:

LEAF ATTACHMENT, ORGANIZATION, SIZE, SHAPE AND PETIOLE FEATURES

LEAF ATTACHMENT:	Petiolate	LEAFLET ORGANIZATION:	Not Preserved
LEAF ARRANGEMENT:	Not Preserved	LEAFLET ATTACHMENT:	np
LEAF ORGANIZATION:	Not Preserved	PETIOLE ATTACHMENT.:	Not Preserved
BLADE SIZE:	Microphyll	PETIOLE BASE:	Not Preserved
BLADE SHAPE:	Ovate	PETIOLE GLANDS:.	Not Preserved
BLADE RATIO L:W:	2:1	PETIOLE X-SECTION:	Not Preserved
LOBATION:	Unlobed	MARGIN TYPE	Serrate
BASE ASYMMETRY:	Symmetrical	MEDIAL SYMMETRY:	Symmetrical

BASE SHAPE:	Concave-convex	APEX SHAPE:	Acuminate	Special Margin Features:	Not Preserved
BASE ANGLE:	Acute	APEX ANGLE:	Acute	Terminal apex features:	Not Preserved

PRIMARY & SECONDARY VEIN FEATURES

		NAKED BASAL VEINS:	Absent
PRIMARY VENATION:	Pinnate	N° BASAL VEINS:	1 (Interior 2°s: Absent)
MAJOR 2° Framework:	Craspedodromous	MINOR-2° Course:	
MAJOR 2° Attachment:	Decurrent	MAJOR 2° VEIN ANGLE:	Inconsistent
MAJOR 2° SPACING:	Regular	AGROPHIC VEINS:	Absent
INTER-2° proximal course:		INTER-2° VEIN LENGTH:	
INTER-2° distal course:		INTER-2° FREQUENCY:	

TERTIARY VEIN FEATURES

INTERCOSTAL 3°:	Opposite percurrent - convex	PERIMARGINAL VEINS:	None
EPIMEDIAL 3°:	Opposite percurrent	INTER-3° ANGLE TO 1°:	Obtuse to midvein
ADMEDIAL COURSE:	Perpendicular to midvein	EXTERIOR 3° COURSE:	Looped
EXMEDIAL COURSE:	Basiflexed	INTERCOSTAL 3° VARIABILITY:	Consistent

FOURTH & HIGHER ORDER VEIN FEATURES

4° VEIN FABRIC:	Mixed percurrent	VEINLETS -F/E/V/s:	Not Preserved
5° VEIN FABRIC:	Irregular reticulate	TYPE OF F.E.V. BRANCHING:	n.p
MARGINAL VENATION:	Not Preserved	F.E.V.s TERMINATIONS:	Not Preserved
AREOLATION:	Moderately developed	LEAF RANK:	

TOOTH FEATURES

ORDERS OF TEETH:	1	No TEETH/cm:	2	TEETH GLANDULARITY:	Non-specific glandular
TOOTH SHAPE:	cc/rt	TOOTH SPACING:	Regular		
PRINCIPAL VEIN:	Present	SINUS SHAPE:	Rounded		
ACCESSORY VEIN COURSE:	Straight or concave	PRINCIPAL VEIN TERM.:	Marginal, at apex		

NOTES

Specimens used to complete morhopheet is: 1706-40C

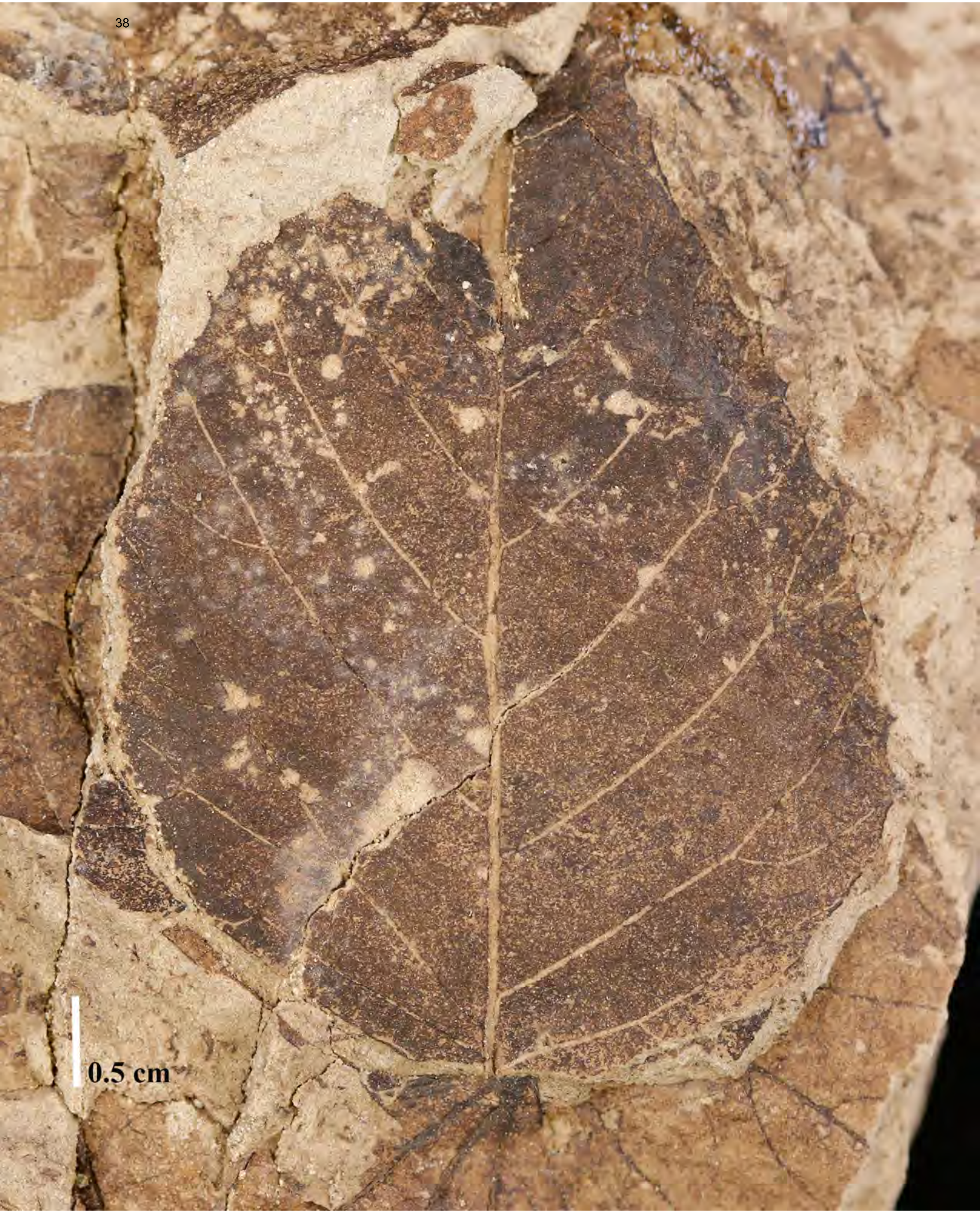
0.5 cm

MORPHOTYPE		NAME	*Trochodendroides genetrix*

GENERAL

MAJOR GROUP		INFERED FAMILY	Trochodendraceae	ORGAN TYPE	Leaf
EXEMPLAR LOC.	HB1706	EXEMPLAR	1706-10	OTHERS LOC.	

DIAGNOSTIC FEATURES OF MORPHOTYPE

Rounded teeth starting approximately half-way up the leaf margin. Teeth are regularly spaced and consistent. Shape can vary but all leaves are obovate

MQI:

LEAF ATTACHMENT, ORGANIZATION, SIZE, SHAPE AND PETIOLE FEATURES

LEAF ATTACHMENT:	Petiolate	LEAFLET ORGANIZATION:	Not Preserved
LEAF ARRANGEMENT:	Not Preserved	LEAFLET ATTACHMENT:	np
LEAF ORGANIZATION:	Not Preserved	PETIOLE ATTACHMENT.:	Not Preserved
BLADE SIZE:	Nanophyll-Notophyll	PETIOLE BASE:	Not Preserved
BLADE SHAPE:	Obovate	PETIOLE GLANDS:.	Not Preserved
BLADE RATIO L:W:	1:1	PETIOLE X-SECTION:	Not Preserved
LOBATION:	Unlobed	MARGIN TYPE	Crenate
BASE ASYMMETRY:	Symmetrical	MEDIAL SYMMETRY:	Symmetrical

BASE SHAPE:	Convex	APEX SHAPE:	Straight	Special Margin Features:	Not Preserved
BASE ANGLE:	Acute	APEX ANGLE:	Obtuse	Terminal apex features:	None

PRIMARY & SECONDARY VEIN FEATURES

		NAKED BASAL VEINS:	Absent
PRIMARY VENATION:	Basal actinodromous	N° BASAL VEINS:	3 — Interior 2°: Present
MAJOR 2° Framework:	Cladodromous	MINOR-2° Course:	Not Preserved
MAJOR 2° Attachment:	Decurrent	MAJOR 2° VEIN ANGLE:	Uniform
MAJOR 2° SPACING:	Regular	AGROPHIC VEINS:	Absent
INTER-2° proximal course:	Not Preserved	INTER-2° VEIN LENGTH:	<50% of subjacent 2°
INTER-2° distal course:	Basiflexed, not perpendicular to subjacent secondary	INTER-2° FREQUENCY:	>1 per intercostal area

TERTIARY VEIN FEATURES

INTERCOSTAL 3°:	Opposite percurrent - straight	PERIMARGINAL VEINS:	Not Preserved
EPIMEDIAL 3°:	Opposite percurrent	INTER-3° ANGLE TO 1°:	Acute to midvein
ADMEDIAL COURSE:	Acute to midvein	EXTERIOR 3° COURSE:	Variable
EXMEDIAL COURSE:	Basiflexed	INTERCOSTAL 3° VARIABILITY:	Consistent

FOURTH & HIGHER ORDER VEIN FEATURES

4° VEIN FABRIC:	Opposite percurrent	VEINLETS -F/E/V/s:	Not Preserved
5° VEIN FABRIC:	Irregular reticulate	TYPE OF F.E.V. BRANCHING:	n.p
MARGINAL VENATION:	Not Preserved	F.E.V.s TERMINATIONS:	Not Preserved
AREOLATION:	Moderately developed	LEAF RANK:	

TOOTH FEATURES

ORDERS OF TEETH:	1	No TEETH/cm:	4	TEETH GLANDULARITY:	None
TOOTH SHAPE:	rt/cv	TOOTH SPACING:	Regular		
PRINCIPAL VEIN:	Present	SINUS SHAPE:	variable		
ACCESSORY VEIN COURSE:	Looped	PRINCIPAL VEIN TERM.:	Marginal, at apex		

NOTES

Morphosheet completed using specimens: 1706-10, 1701-41

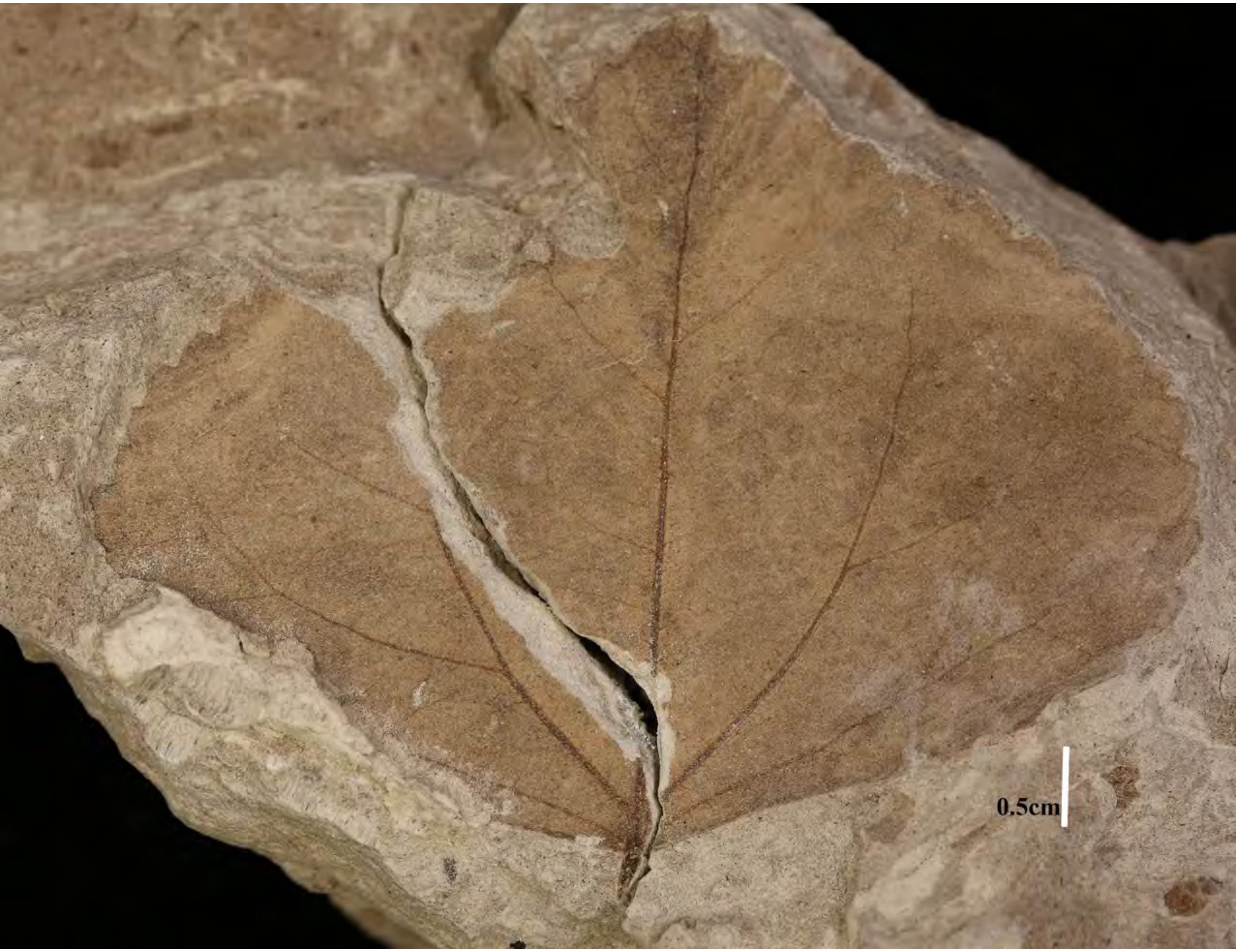
0.5cm

MORPHOTYPE		NAME	*Zizyphoides flabella*

GENERAL

MAJOR GROUP	DIC	INFERED FAMILY		ORGAN TYPE	Leaf
EXEMPLAR LOC.	1701	EXEMPLAR	1701-84, 1706-36B	OTHERS LOC.	

DIAGNOSTIC FEATURES OF MORPHOTYPE: Similar overall shape to Trochodendroides but without teeth. In lue of teeth, Zizyphoides has "lobes" within the top 1/3 of the leaf, similar to Platanties (Palmate). 2° framework is simple brochidodromous and cladodromous. Lastly, Zizyphoides has 5 basal veins.

MQI:

LEAF ATTACHMENT, ORGANIZATION, SIZE, SHAPE AND PETIOLE FEATURES

LEAF ATTACHMENT:	Petiolate	LEAFLET ORGANIZATION:	Not Preserved
LEAF ARRANGEMENT:	Not Preserved	LEAFLET ATTACHMENT:	np
LEAF ORGANIZATION:	Not Preserved	PETIOLE ATTACHMENT.:	Not Preserved
BLADE SIZE:	Microphyll	PETIOLE BASE:	Not Preserved
BLADE SHAPE:	Elliptic	PETIOLE GLANDS:.	Not Preserved
BLADE RATIO L:W:	1:1	PETIOLE X-SECTION:	Not Preserved
LOBATION:	Unlobed	MARGIN TYPE	Untoothed
BASE ASYMMETRY:	Symmetrical	MEDIAL SYMMETRY:	Symmetrical

BASE SHAPE:	Concave	APEX SHAPE:	Straight	Special Margin Features:	Not Preserved
BASE ANGLE:	Obtuse	APEX ANGLE:	Wide obtuse	Terminal apex features:	None

PRIMARY & SECONDARY VEIN FEATURES

		NAKED BASAL VEINS:	Absent
PRIMARY VENATION:	Basal actinodromous	N° BASAL VEINS:	5
		Interior 2°:	Absent
MAJOR 2° Framework:	Mixed framework	MINOR-2° Course:	
MAJOR 2° Attachment:	Decurrent	MAJOR 2° VEIN ANGLE:	Smoothly decreasing toward base
MAJOR 2° SPACING:	Decreasing proximally	AGROPHIC VEINS:	Absent
INTER-2° proximal course:		INTER-2° VEIN LENGTH:	
INTER-2° distal course:		INTER-2° FREQUENCY:	

TERTIARY VEIN FEATURES

INTERCOSTAL 3°:	Not Preserved	PERIMARGINAL VEINS:	Not Preserved
EPIMEDIAL 3°:	Not preserved	INTER-3° ANGLE TO 1°:	Not Preserved
ADMEDIAL COURSE:	Not Preserved	EXTERIOR 3° COURSE:	Not Preserved
EXMEDIAL COURSE:	Not Preserved	INTERCOSTAL 3° VARIABILITY:	Not Preserved

FOURTH & HIGHER ORDER VEIN FEATURES

4° VEIN FABRIC:	Not Preserved	VEINLETS -F/E/V/s:	Not Preserved
5° VEIN FABRIC:	Not Preserved	TYPE OF F.E.V. BRANCHING:	n.p
MARGINAL VENATION:	Not Preserved	F.E.V.s TERMINATIONS:	Not Preserved
AREOLATION:	Not Preserved	LEAF RANK:	

TOOTH FEATURES

ORDERS OF TEETH:		No TEETH/cm:		TEETH GLANDULARITY:	
TOOTH SHAPE:		TOOTH SPACING:			
PRINCIPAL VEIN:		SINUS SHAPE:			
ACCESSORY VEIN COURSE:		PRINCIPAL VEIN TERM.:			

NOTES

Specimens used to complete morphosheet: 1701-84, 1706-36B

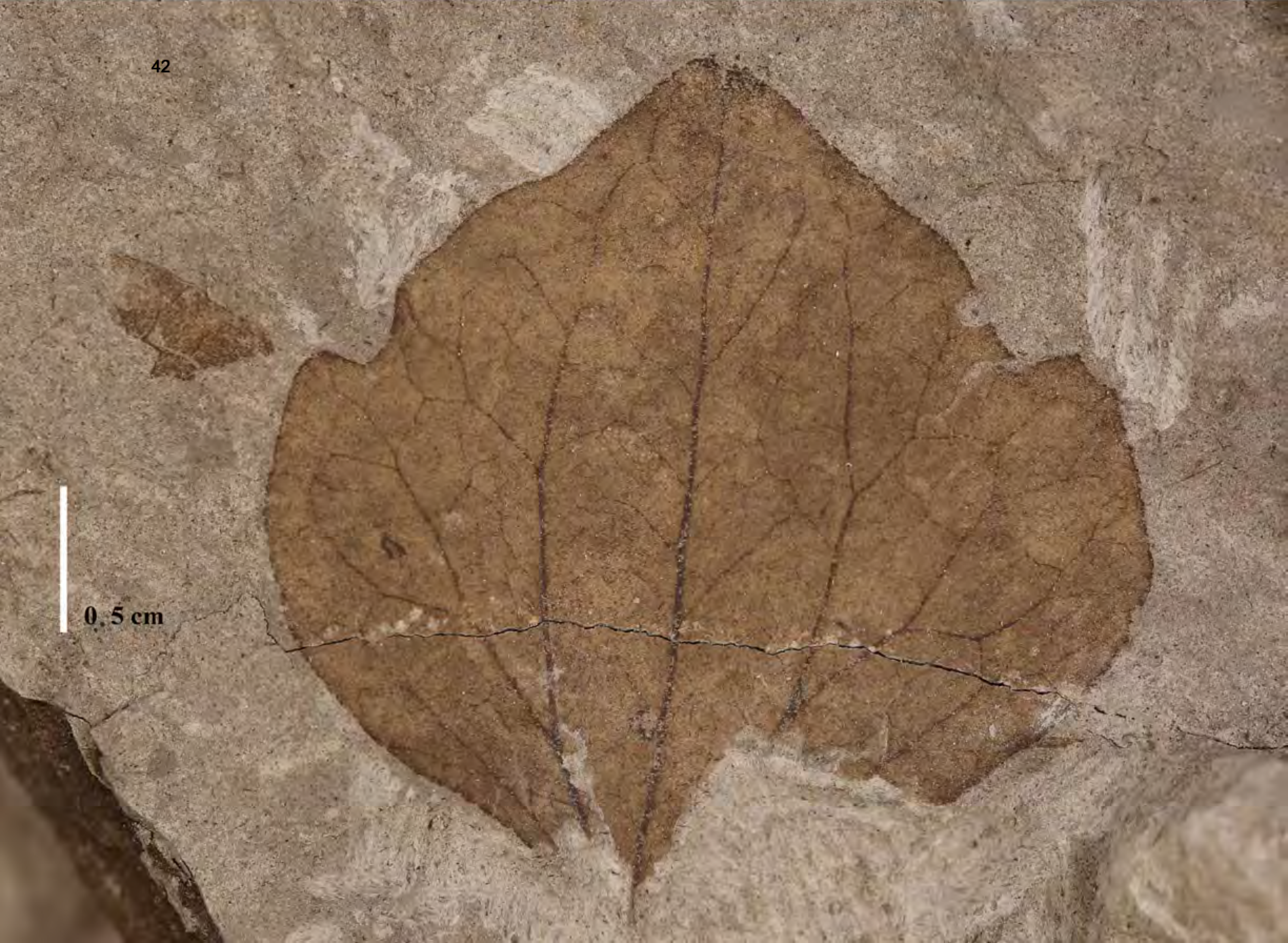
0. 5 cm
