# Supplementary material for: Plant and insect herbivore community variation across the Paleocene–Eocene boundary in the Hanna Basin, southeastern Wyoming
Source: PeerJ. 2019 Oct 15;7:e7798. doi: 10.7717/peerj.7798 (PMC6798869; doi:10.7717/peerj.7798)
Supplement: Supplemental Information 5 — Summary data tables for each quarry described in this study. Previously collected quarries, Dunn, 2003, are also summarized here. Table (K) represents the distribution of morphotypes across all stratigraphic levels. [file peerj-07-7798-s005.pdf]

Table A1. SUMMARY DATA FOR A.1 JINGO- PALEOCENE

Morphotype Number	Morphospecies	**Total**	Relative Abundance %	%Mi	%No	%Me	%Ma	%Na	Margin
HB022		**11**	6.7	0	50	50	0	0	T
HB025		**1**	0.6	0	100	0	0	0	U
HB028		**12**	7.3	33.33	33.33	33.33	0	0	U
HB032		**2**	1.2	0	100	0	0	0	U
HB038		**1**	0.6	0	100	0	0	0	U
HB046		**1**	0.6	0	100	0	0	0	T
HB077		**48**	29.1	33.33	33.33	33.33	0	0	T
HB104		**46**	27.9	0	50	50	0	0	U
HB121		**1**	0.6	0	100	0	0	0	U
HB122		**13**	7.9	0	100	0	0	0	U
HB134		**4**	2.4	0	100	0	0	0	T
HB144		**3**	1.8	0	100	0	0	0	T
HB161		**5**	3.0	0	50	50	0	0	T
HB164		**12**	7.3	0	0	100	0	0	U
HB166		**1**	0.6	0	100	0	0	0	U
HB167		**2**	1.2	0	100	0	0	0	U
HB168		**2**	1.2	0	100	0	0	0	T
Total		**165**							

Table A2. SUMMARY DATA FOR A.2-PALEOCENE [LAT. Cont. of Jingo]

Morphotype Number	Morphospecies	**Total**	Relative Abundance %	%Mi	%No	%Me	%Ma	%Na	Margin
	Averrhoites affinis	**1**	1.1	100	0	0	0	0	U
	Fagopsiphyllum groenlandicum	**2**	2.2	100	0	0	0	0	T
HB172		**4**	4.3	33.33	33.33	0	0	33.33	T
HB173		**7**	7.6	50	50	0	0	0	U
HB174		**25**	27.2	25	25	25	0	25	T
HB176		**18**	19.6	33.33	33.33	33.3	0	0	U
HB177		**5**	5.4	50	0	0	0	50	U
HB178		**6**	6.5	50	50	0	0	0	U
HB182		**19**	20.7	33.33	33.33	33.3	0	0	U
HB183		**2**	2.2	0	100	0	0	0	U
	Platanites raynoldsii	**1**	1.1	0	100	0	0	0	T
	Trochodendroides genetrix	**1**	1.1	0	100	0	0	0	T
	Zizyphoides flabella	**1**	1.1	100	0	0	0	0	T
Total		**92**							

Table A3. SUMMARY DATA FOR B- PALEOCENE

Morphotype Number	Morphospecies	**Total**	Relative Abundance %	%Mi	%No	%Me	%Ma	%Na	Margin
	Averrhoites affinis	**158**	36.4	50	50	0	0	0	U
HB019		**1**	0.2	0	100	0	0	0	T
HB022		**17**	3.9	33.33	33.33	33.33	0	0	T
HB023		**2**	0.5	0	100	0	0	0	U
HB025		**13**	3.0	0	50	50	0	0	U
HB026		**36**	8.3	50	50	0	0	0	T
HB028		**11**	2.5	0	50	50	0	0	U
HB031		**2**	0.5	0	100	0	0	0	U
HB032		**9**	2.1	50	50	0	0	0	U
HB042		**2**	0.5	0	0	0	0	0	U
HB046		**3**	0.7	100	0	0	0	0	T
HB050		**3**	0.7	0	100	0	0	0	U
HB076		**2**	0.5	50	0	50	0	0	U
HB077		**80**	18.4	33.33	33.33	33.33	0	0	T
HB104		**30**	6.9	33.33	33.33	33.33	0	0	U
HB121		**19**	4.4	50	50	0	0	0	U
HB127		**3**	0.7	0	50	50	0	0	U
HB134		**2**	0.5	50	50	0	0	0	T
HB136		**2**	0.5	0	50	50	0	0	T
HB138		**2**	0.5	0	50	50	0	0	T
HB144		**4**	0.9	50	50	0	0	0	T
HB145		**1**	0.2	0	100	0	0	0	U
HB161		**2**	0.5	0	0	100	0	0	T
HB168		**2**	0.5	0	100	0	0	0	T
HB174		**20**	4.6	100	0	0	0	0	T
HB175		**1**	0.2	50	50	0	0	0	T
HB176		**1**	0.2	25	25	25	25	0	U
HB177		**6**	1.4	50	50	0	0	0	U
Total		**434**							

Table A4. SUMMARY DATA FOR C.1- PALEOCENE (COAL 80)

Morphotype Number	Morphospecies	**Total**	Relative Abundance %	%Mi	%No	%Me	%Ma	%Na	Margin
	Averrhoites affinis	**1**	0.4	100	0	0	0	0	U
HB172		**3**	1.3	0	100	0	0	0	T
HB173		**1**	0.4	0	100	0	0	0	U
HB174		**3**	1.3	50	50	0	0	0	T
HB176		**12**	5.2	33.3	33.3	33.3	0	0	U
HB177		**3**	1.3	100	0	0	0	0	U
HB178		**1**	0.4	0	100	0	0	0	U
HB179		**1**	0.4	100	0	0	0	0	U
HB183		**1**	0.4	0	0	0	0	100	U
	Platanites raynoldsii	**136**	59.4	25	25	25	0	25	T
	Trochodendroides genetrix	**53**	23.1	25	25	25	0	25	T
	Zizyphoides flabella	**14**	6.1	50	0	0	0	50	U
Total		**229**							

Table A5. SUMMARY DATA FOR C.2-PALEOCENE (coal 80)

Morphotype Number	Morphospecies	**Total**	Relative Abundance %	%Mi	%No	%Me	%Ma	%Na	Margin
	Averrhoites affinis	**2**	2.7	100	0	0	0	0	U
HB171		**1**	1.3	0	100	0	0	0	U
HB172		**1**	1.3	100	0	0	0	0	T
HB174		**1**	1.3	100	0	0	0	0	T
HB176		**1**	1.3	100	0	0	0	0	U
HB177		**1**	1.3	0	100	0	0	0	U
HB178		**1**	1.3	100	0	0	0	0	U
	Platanites raynoldsii	**32**	42.7	25	25	25	0	25	T
	Trochodendroides genetrix	**33**	44.0	25	25	25	0	25	T
	Zizyphoides flabella	**2**	2.7	100	0	0	0	0	U
Total		**75**							

Table A6. SUMMARY DATA FOR D.1-EOCENE [PETM rebound]

Morphotype Number	Morphospecies	**Total**	Relative Abundance %	%Mi	%No	%Me	%Ma	%Na	Margin
HB173		**1**	20	100	0	0	0	0	U
HB174		**1**	20	0	0	0	0	100	T
HB175		**1**	20	100	0	0	0	0	T
HB176		**2**	40	0	100	0	0	0	U
Total		**5**							

Table A7. SUMMARY DATA FOR D.2-EOCENE [PETM rebound]

Morphotype Number	Morphospecies	**Total**	Relative Abundance %	%Mi	%No	%Me	%Ma	%Na	Margin
	Averrhoites affinis	**1**	2.5	100	0	0	0	0	U
HB172		**1**	2.5	100	0	0	0	0	T
HB173		**3**	7.5	50	50	0	0	0	U
HB174		**2**	5	50	50	0	0	0	U
HB176		**9**	22.5	33.3	33.3	33.3	0	0	U
HB177		**1**	2.5	100	0	0	0	0	U
HB178		**7**	17.5	50	50	0	0	0	U
HB179		**4**	10	50	50	0	0	0	U
HB180		**11**	27.5	33.3	33.3	33.3	0	0	U
HB182		**1**	2.5	100	0	0	0	0	U
Total		**40**							

Table A8. SUMMARY DATA FOR D.3-EOCENE [PETM rebound]

Morphotype Number	Morphospecies	**Total**	Relative Abundance %	%Mi	%No	%Me	%Ma	%Na	Margin
HB173		**1**	9.1	100	0	0	0	0	U
HB176		**5**	45.5	33.3	33.3	33.3	0	0	U
HB177		**1**	9.1	100	0	0	0	0	U
HB178		**2**	18.2	50	50	0	0	0	U
	Platanites raynoldsii	**1**	9.1	0	0	100	0	0	T
	Trochodendroides genetrix	**1**	9.1	100	0	0	0	0	T
Total		**11**							

Table A9. SUMMARY DATA FOR E.1-EOCENE

Morphotype Number	Morphospecies	**Total**	Relative Abundance %	%Mi	%No	%Me	%Ma	%Na	Margin
	Averrhoites affinis	**1**	1.8	0	0	100	0	0	U
	Fagopsiphyllum groenlandicum	**3**	5.5	50	50	0	0	0	T
HB172		**1**	1.8	100	0	0	0	0	T
HB175		**8**	14.5	50	50	0	0	0	T
HB176		**19**	34.5	33.3	33.3	33.3	0	0	U
HB177		**2**	3.6	100	0	0	0	0	U
HB178		**1**	1.8	50	50	0	0	0	U
HB179		**1**	1.8	100	0	0	0	0	U
HB183		**1**	1.8	0	100	0	0	0	U
	Macginitiea gracilis	**4**	7.3	0	33.3	33.3	33.3	0	U
	Platanites raynoldsii	**12**	21.8	25	25	25	25	0	T
	Trochodendroides genetrix	**2**	3.6	100	0	0	0	0	T
TOTAL		**55**							

Table A10. SUMMARY DATA FOR E.2- EOCENE

Morphotype Number	Morphospecies	**Total**	Relative Abundance %	%Mi	%No	%Me	%Ma	%Na	Margin
	Alnus sp.	**1**	0.4	0	100	0	0	0	T
	Averrhoites affinis	**3**	1.1	50	50	0	0	0	U
HB172		**1**	0.4	100	0	0	0	0	T
HB173		**10**	3.7	50	50	0	0	0	U
HB174		**1**	0.4	100	0	0	0	0	T
HB175		**4**	1.5	50	50	0	0	0	T
HB176		**155**	58.1	25	25	25	25	0	U
HB177		**25**	9.4	50	50	0	0	0	U
HB178		**21**	7.9	33.3	33.3	33.3	0	0	U
HB179		**1**	0.4	100	0	0	0	0	U
HB181		**1**	0.4	0	100	0	0	0	U
HB182		**3**	1.1	50	50	0	0	0	U
HB183		**30**	11.2	33.3	33.3	33.3	0	0	U
	Macginitiea gracilis	**1**	0.4	0	0	100	0	0	U
	Platanites raynoldsii	**9**	3.4	33.3	33.3	33.3	0	0	T
	Trochodendroides genetrix	**1**	0.4	0	0	100	0	0	T
Total		**267**							

TABLE A11: DISTRIBUTION OF MORPHOTYPES ACROSS STRATIGRAPHIC LEVELS

Morphotype	A	B	C	D	E
Alnus sp.					X
Averrhoites affinis	X	X	X	X	X
Fagopsiphyllum groenlandicum	X				X
HB022	X	X			
HB023		X			
HB025	X	X			
HB026	X	X			
HB028	X	X			
HB030		X			
HB031		X			
HB032	X	X			
HB038	X				
HB042		X			
HB046	X	X			
HB050		X			
HB065		X			
HB076		X			
HB077	X	X			
HB104	X	X			
HB121	X	X			
HB122	X	X			
HB127		X			
HB134	X	X			
HB136		X			
HB138		X			
HB144	X	X			
HB145		X			
HB161	X	X			
HB164	X				
HB166	X				
HB167	X				
**Morphotype**	**A**	**B**	**C**	**D**	**E**
HB168	X	X			
HB171			X		
HB172	X		X	X	X
HB173	X		X	X	
HB174	X		X	X	
HB175		X		X	X
HB176	X	X	X	X	X
HB177	X	X	X	X	X
HB178	X		X	X	X
HB179			X	X	X
HB180				X	

HB181				
HB182	X		X	
HB183	X	X		X
Macginitiea gracilis				X
Platanites raynoldsii	X	X	X	X
Trochodendroides genetrix	X	X	X	X
Zizyphoides flabella	X	X		
